# Biological Activity of Recently Discovered Halogenated Marine Natural Products

**DOI:** 10.3390/md13074044

**Published:** 2015-06-30

**Authors:** Gordon W. Gribble

**Affiliations:** Department of Chemistry, Dartmouth College, Hanover, NH 03755, USA; E-Mail: gordon.w.gribble@dartmouth.edu; Tel.: +1-603-646-3118; Fax: +1-603-646-3946

**Keywords:** organohalogen, antibacterial, antiparasitic, antiviral, antitumor, antiinflammatory, antioxidant, natural products, organochlorine, organobromine

## Abstract

This review presents the biological activity—antibacterial, antifungal, anti-parasitic, antiviral, antitumor, antiinflammatory, antioxidant, and enzymatic activity—of halogenated marine natural products discovered in the past five years. Newly discovered examples that do not report biological activity are not included.

## 1. Introduction

From fewer than 50 examples of halogenated natural products that were known in 1968 [1], the number today is more than 5000 and steadily increasing [2,3,4,5,6]. A majority of these compounds are found in marine organisms and several recent reviews are available of marine natural products in general [7,8,9,10], in algae [11,12,13], in sponges [14,15,16], in invertebrates [17,18,19], in gorgonians [20], in bryophytes [21], in fungi [22], in cyanobacteria [23], in marine bacteria [24], and those cyano-containing marine triterpenoids [25]. Given the enormous advance in ocean exploration, including retrieving samples at depths reaching 11,000 m [26], it is inevitable that novel marine natural products are awaiting discovery.

In the present review I have chosen to focus on halogenated marine natural products possessing demonstrated biological activity that were reported during the period 2011–2015. My organization is according to the type of observed activity, and many of these marine metabolites have multiple activities and therefore appear in more than one section.

## 2. Antibacterial Activity

Natural products represent an important potential source of new antibacterial drugs [27], particularly those that prevent biofouling by barnacles, tubeworms, mussels, and other “smothering” marine organisms [28,29].

**Figure 1:**
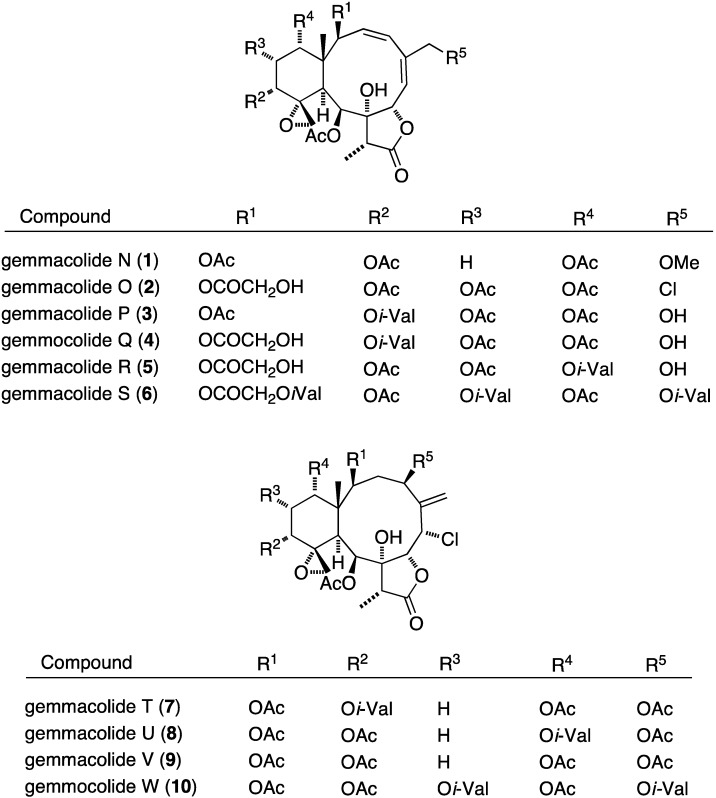

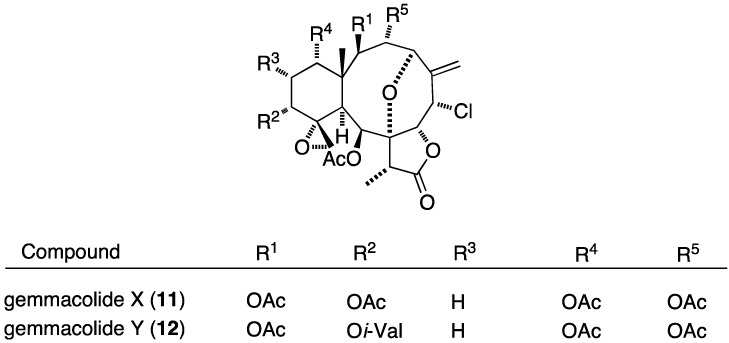
Structures of gemmacolide briarane diterpenoids from *Dichotella gemmacea* [30,31].

Many gorgonian soft coral metabolites are biofilm inhibitors. For example, the South China Sea gorgonian *Dichotella gemmacea* has yielded several antibacterial briarane diterpenoids **1**–**12**, several of which contain chlorine, as summarized in Figure 1 [30,31].

Of these gemmacolides, N (**1**), O (**2**), and Q (**4**) show antibacterial activity against the Gram-negative bacterium *E. coli* in the agar diffusion assay, with the chlorinated gemmacolide O being the most active [30]. Antitumor and antifungal activities are discussed in the appropriate sections to follow.

**Figure 2:**
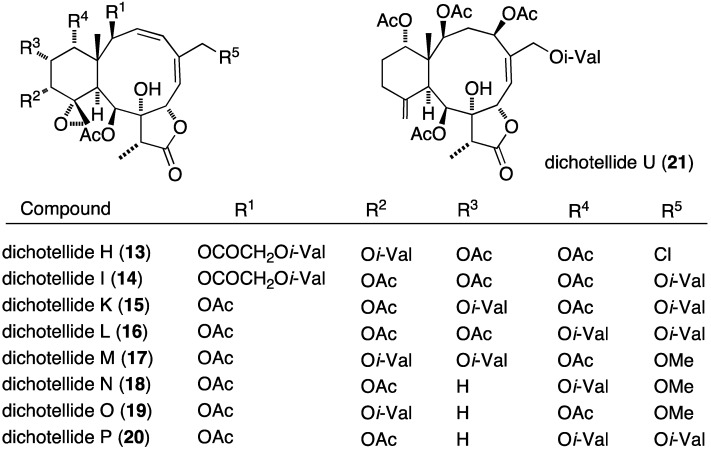
Structures of selected dichotellide briarane diterpenoids from *Dichotella gemmacea* [34].

The prolific gorgonian *Dichotella gemmacea* is also the source of numerous new briarane diterpenoids, the dichotellides, many of which contain chlorine or iodine or both [32,33,34]. In particular, of the 16 novel briarane diterpenoids, dichotellides F–U, found in *Dichotella gemmacea*, H (**13**), I (**14**), K (**15**), L (**16**), M (**17**), N (**18**), O (**19**), P (**20**), and U (**21**) exhibit potent antifouling activities (Figure 2; Table 1) [34].

**Table 1:** Biofouling activity of dichotellides (**13**–**21**) against the larval settlement of the barnacle *Balanus amphitrite* [34].

Compound	EC_50_ (μg/mL) ^a^	LC_50_/EC_50_^b^
H (**13**)	4.1	>24
I (**14**)	1.82	>54.9
K (**15**)	6.3	>16
L (**16**)	7.6	>13
M (**17**)	4.6	>11
N (**18**)	1.2	>88
O (**19**)	5.6	>18
P (**20**)	0.79	>126.6
U (**21**)	2.0	>48

^a^ Effective concentration for 50% inhibition; ^b^ Lethal concentration/effective concentration.

**Figure 3:**
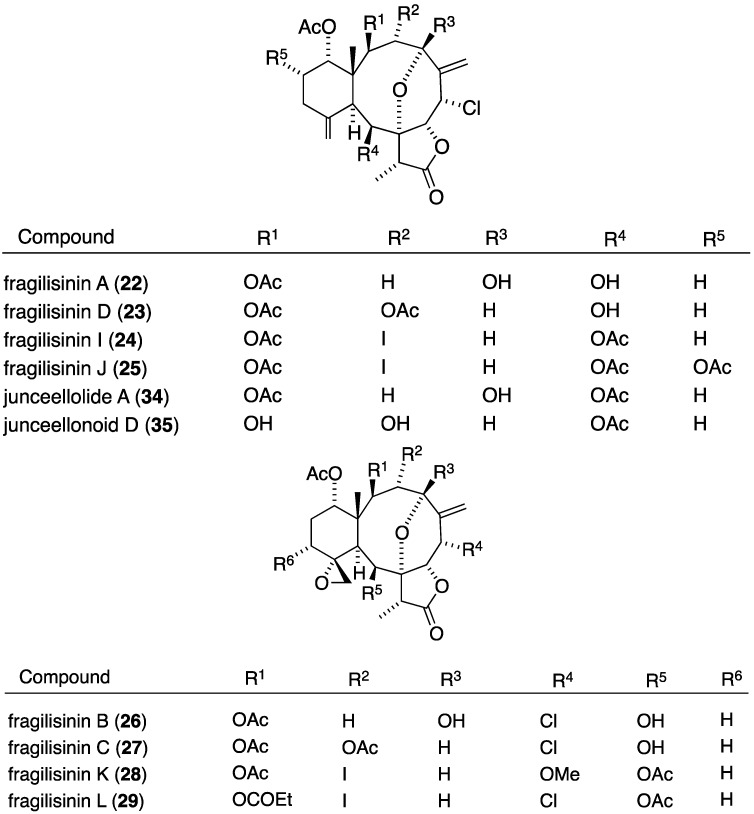

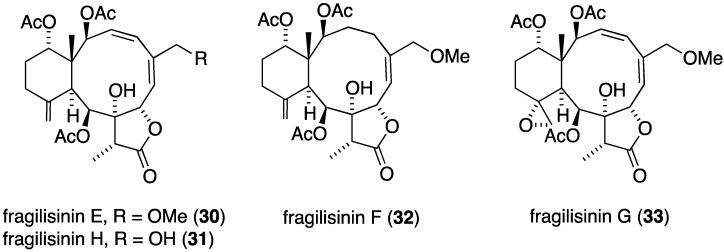
Structures of fragilisinins A–L briarane diterpenoids from *Junceella fragilis* [35].

The South China Sea gorgonian *Junceella fragilis* has also yielded 12 new briarane diterpenoids, fragilisinins A–L (**22**–**33**) (Figure 3), several of which have potent antifouling activity, but are not superior to the previously known junceelloide A and junceellonoid D (Table 2) [35].

**Table 2:** Biofouling activity of fragilisinins against the larval settlement of the barnacle *Balanus amphitrite* [35].

Compound	EC_50_ (μM)	LC_50_/EC_50_
fragilisinin E (**30**)	14.0	>13
fragilisinin F (**32**)	12.6	>14.5
fragilisinin J (**25**)	11.9	>11.5
junceellolide A (**34**)	5.6	>33.3
junceellonoid D (**35**)	10.0	>20
positive control ^a^	2.5	–

^a^ 5-octylfuran-2(5*H*)-one.

**Figure 4:**
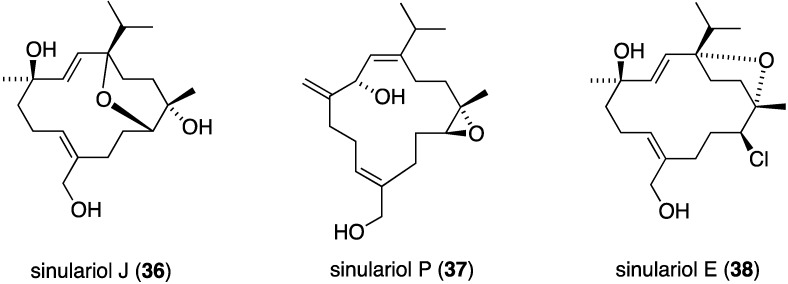
Structures of selected sinulariol cembrane diterpenoids from *Sinularia rigida* [36].

An examination of the Chinese soft coral *Sinularia rigida* has yielded 19 new cembrane diterpenoids, the sinulariols, of which J (**36**) and P (**37**) display antifouling activity against *B. amphitrite* (5.65 μg/mL) and *B. neritina* (14.03 µg/mL), respectively (Figure 4). The one chlorine-containing example, sinulariol E (**38**) is less active [36].

**Figure 5:**
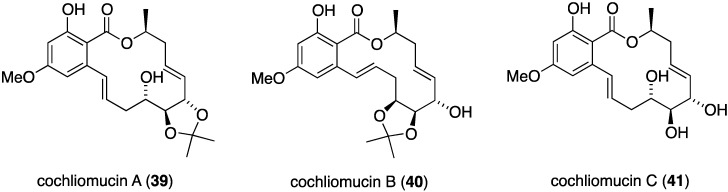
Structures of cochliomycins A–C (**39**–**41**) from *Cochliobolus lunatus* [37,38].

Potent antifouling activity is observed in some newly isolated resorcylic acid lactones found in the fungus *Cochlionbolus lunatus* derived from the gorgonian *Dichotella gemmacea*. Thus obtained were cochliomycins A–C (**39**–**41**) (Figure 5) [37,38]. Only cochliomycin A (**39**) shows potent activity against *Balanus amphitrite* (EC_50_ 1.2 μg/mL; LC_50_/EC_50_ > 16.7), which was superior to the known analogues zeaenol, LL-Z1640-1, and LL-Z1640-2. Insufficient material of cochliomycins B and C was available for testing.

**Figure 6:**
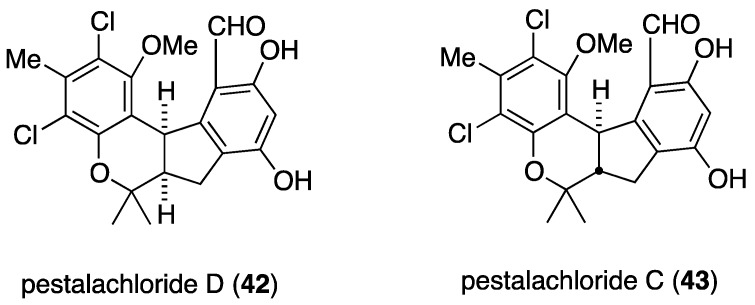
Structures of (±)-pestalachlorides D (**42**) and C (**43**) from *Pestalotiopsis* sp. [39,40].

Another soft-coral derived fungus, *Pestalotiopsis* sp. from the South China Sea *Sarcophyton* sp., contains the novel (±)-pestalachloride D (**42**) and the known analogue (±)-pestalachloride C (**43**) (Figure 6) [39,40]. Both compounds are active against the bacteria *E. coli*, *Vibrio anguillarum*, and *Vibrio parahaemolyticus* with MIC values of 5.0, 10.0, and 20.0 µM, respectively [39].

**Figure 7:**
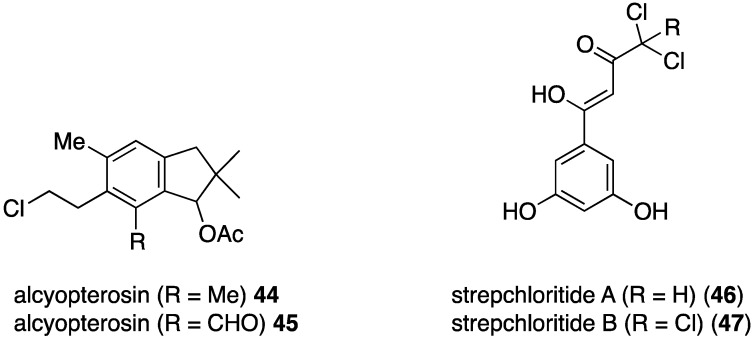
Structures of alcyopterosins **44** and **45** from *Alcyonium roseum* [41], and strepchloritides A (**46**) and B (**47**) from *Streptomyces* sp. OUCMDZ-1703 [42].

The Antarctic soft coral *Alcyonium roseum* has yielded the two new illudalanes, alcyopterosins **44** and **45** (Figure 7) [41]. Although insufficient material was available for antibacterial testing, the authors believe that these metabolites may be feeding deterrents for the predatory sea star *Odontaster validus* and have antifouling activity, based on similar properties of related alcyopterosins. The soft-coral associated actinomycetes strain, *Streptomyces* sp. OUCMDZ-1703 has yielded the novel strepchloritides A (**46**) and B (**47**), which exhibit modest activity against *E. coli*, *Pseudomonas aeruginosa*, and *S. aureus* (Figure 7).

**Figure 8:**
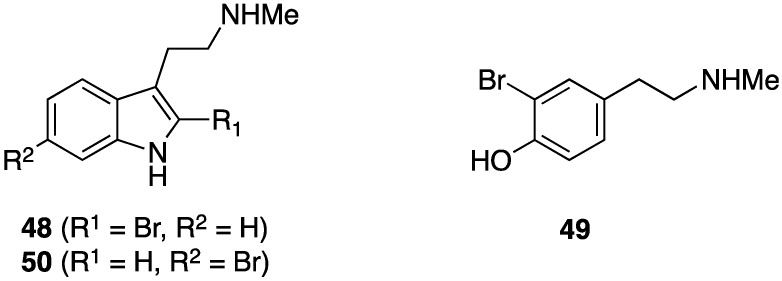
Brominated compounds **48**–**50** from *Paramuricea clavata* [43].

The Mediterranean gorgonian *Paramuricea clavata* is reported to contain three new brominated metabolites, 2-bromo-*N*-methyltryptamine (**48**), 3-bromo-*N*-methyltyramine (**49**), and 6-bromo-*N*-methyltryptamine (**50**) (Figure 8) in addition to several known analogues [43]. Compound **50** was previously known from synthesis. Of the ten compounds tested, **48** shows the highest activity in preventing adhesion of three bacterial strains (*Pseudoalteromonas* sp. D41 and TC8, and *Paracoccus* sp. 4M6). However, insufficient material was available for toxicity screening.

**Figure 9:**
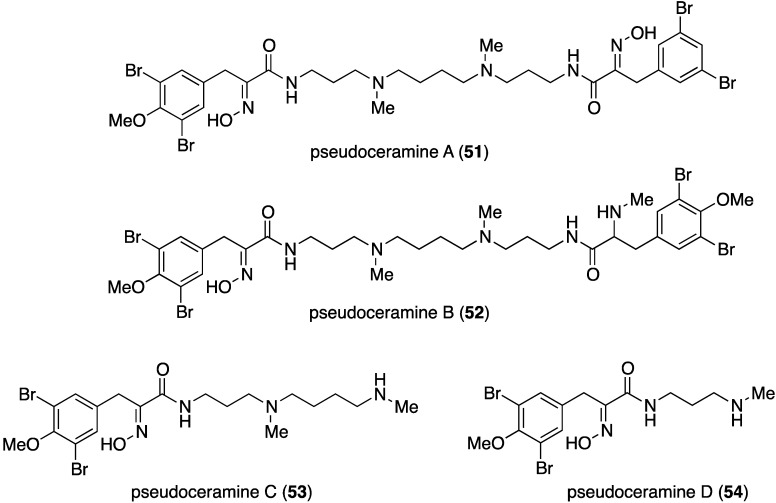
Structures of pseudoceramines A–D (**51**–**54**) from the sponge *Pseudoceratina* sp. [44].

The marine sponge *Pseudoceratina* sp. has yielded numerous brominated alkaloids with biological activity [2,3,4], including the four new pseudoceramines A–D (**51**–**54**) collected from this sponge in the Great Barrier Reef, Queensland, Australia (Figure 9) [44]. Pseudoceramine B (**52**) inhibits bacterial growth with IC_50_ 40 µM.

**Figure 10:**
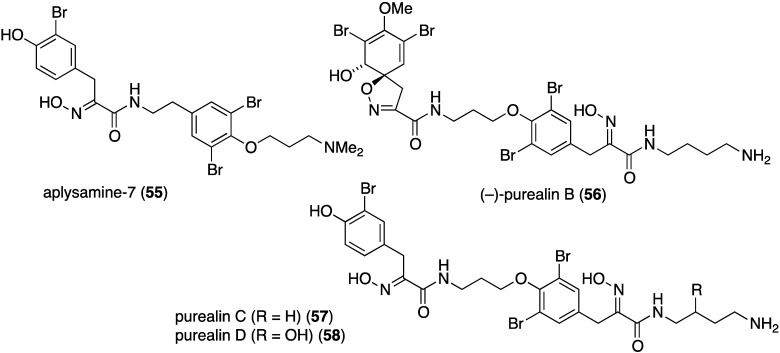
Structures of bromotyrosines **55**–**58** from the sponge *Pseudoceratina* sp. [45].

Of the 12 bromotyrosines isolated from the southern Australian sponge *Pseudoceratina* sp., four were new metabolites: aplysamine-7 (**55**), (–)-purealin B (**56**), purealin C (**57**), and purealin D (**58**) (Figure 10) [45]. Purealin C shows a broad spectrum of activity against two strains each of the Gram-positive *S. aureus* (IC_50_ 2.6 and 6.2 µM) and *B. subtilis* (IC_50_ 2.6 and 2.8 µM), while (–)-purealin B is only active against *B. subtilis* (IC_50_ 3.4 and 3.8 µM).

**Figure 11:**
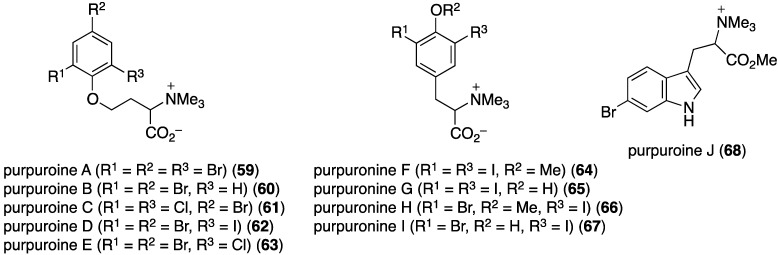
Structures of purpuroines A–J (**59**–**68**) from the sponge *Iotrochota purpurea* [46].

A collection of the sponge *Iotrochota purpurea* from Hainan Island, China, has yielded the ten new halogenated purpuroines A–J (**59**–**68**), five of which contain iodine (Figure 11) [46]. In addition to antifungal activity to be discussed in the next section, purpuroine I (**67**) shows selective inhibition of the human pathogen *Streptococcus pneumonia* (IC_50_ 18.06 ± 0.76 µg/mL; ampicillin, IC_50_ 0.38 ± 0.029 µg/mL).

**Figure 12:**
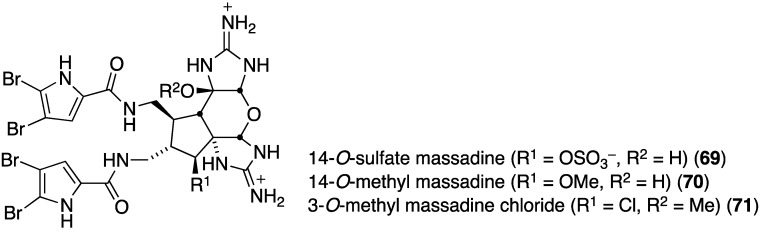
Structures of massadines **69**–**71** from the sponge *Axinella* sp. [47].

The deep-sea Great Australian Bight sponge, *Axinella* sp., contains the three new brominated imidazoles, 14-*O*-sulfate massadine (**69**), 14-*O*-methyl massadine (**70**), and 3-*O*-methyl massadine chloride (**71**) (Figure 12) [47]. The latter chlorine-containing metabolite (**71**) exhibits antibacterial activity against the Gram-positive bacteria *Staphylococcus aureus* (ATCC 9144 and 25923; IC_50_ 3.7 and 4.2 µM, respectively) and *B. subtilis* (ATCC 6051 and 6633; IC_50_ 2.6 and 2.2 µM, respectively), and the Gram-negative bacteria *E. coli* (ATCC 11775; IC_50_ 4.4 µM) and *P. aeruginosa* (ATCC 10145; IC_50_ 4.9 µM). The effect of the chlorine atom is noteworthy.

**Figure 13:**
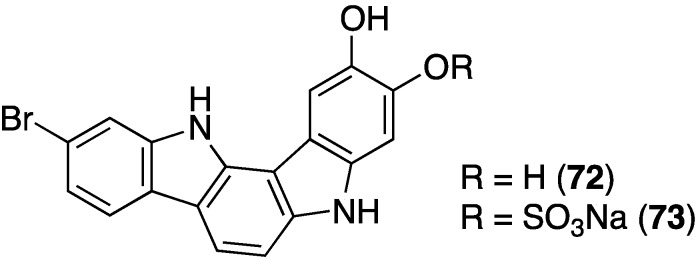
Structures of indolo[3,2-*a*]carbazoles **72** and **73** from the sponge *Asteropus* sp. [48].

A deep-water *Asteropus* sponge from the Bahamas contains the novel indolo[3,2-*a*]carbazoles **72** and **73** (Figure 13); **72** shows some activity against methicillin-resistant *S. aureus* (MRSA; minimum inhibitory concentration (MIC) of 50 µg/mL [48].

**Figure 14:**
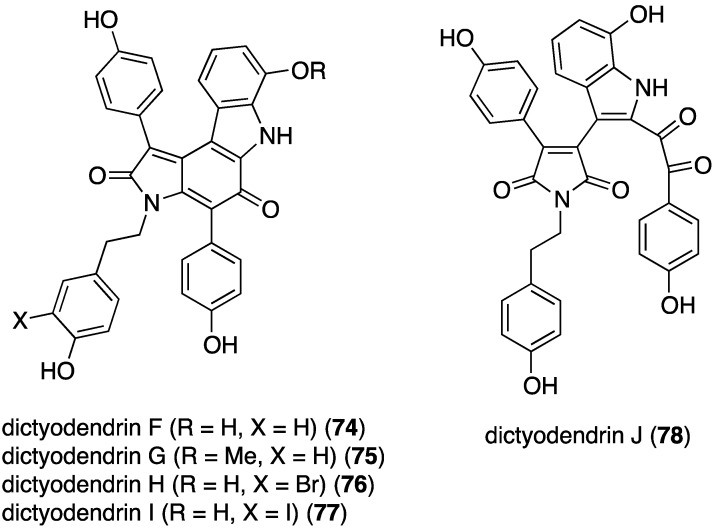
Structures of dictyodendrins F–J (**74**–**78**) from the sponge *Ianthella* sp. [49].

Examination of the southern Australian sponge *Ianthella* sp. has revealed the presence of dictyodendrins F–J (**74**–**78**) (Figure 14) [49]. Antibacterial activity is limited to the Gram-positive *B. subtilis* (ATCC 6051 and 6633): **74** (IC_50_ 2.7 and 2.3 µM), **76** (IC_50_ 1.2 and 3.1 µM), and **77** (IC_50_ 2.5 and 2.8 µM).

**Figure 15:**
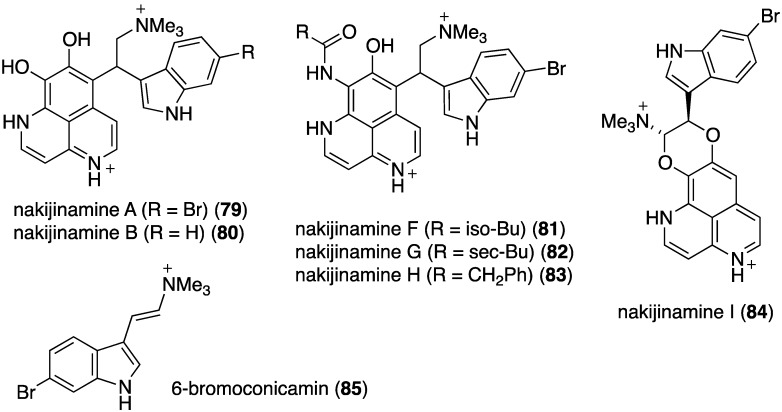
Structures of nakijinamines **79**–**84** and 6-bromoconicamin (**85**) from the sponge *Suberites* sp. [50].

A series of structurally novel indole alkaloids was isolated from the Okinawan sponge *Suberites* sp., including nakijinamines A (**79**), B (**80**), F (**81**), G (**82**), H (**83**), I (**84**), and 6-bromoconicamin (**85**) (Figure 15) [50]. An earlier study by this same research team identified the related nakijinamines C–E (not shown) [51]. Of these alkaloids, only nakijinamine A (**79**) is active against *S. aureus* (MIC 16 µg/mL), *B. subtilis* (MIC 16 µg/mL), and *Micrococcus luteus* (MIC 2 µg/mL). Nakijinamine I (**84**) is the first aaptamine-type alkaloid to have a 1,4-dioxane unit.

**Figure 16:**
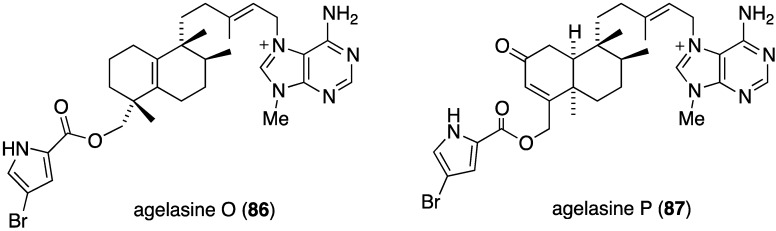

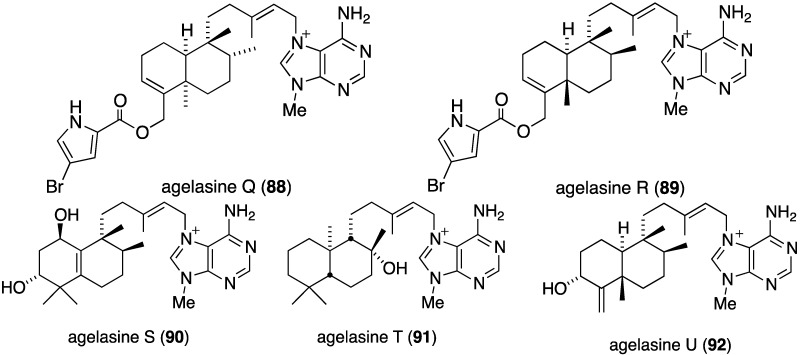
Structures of agelasines O–U (**86**–**92**) from the sponge *Agelas* sp. [52].

The Okinawan sponge *Agelas* sp. is a rich source of brominated pyrrole alkaloids and several recent studies have added to this collection. The agelasines O–U (**86**–**92**) from *Agelas* sp. (NSS-19) are novel diterpene alkaloids tethered to a 9-*N*-methyladenine unit (Figure 16) [52]. Of these alkaloids, only agelasines O–R (**86**–**89**) and T (**91**) show activity against *S. aureus* and *B. subtilis* (MIC 8.0–32.0 µg/mL), but not against *E. coli* (MIC ≥ 32.0 µg/mL). For both strains the activity decreases: Q (**88**) ~ R (**89**) > O (**86**) ~ T (**91**) > P (**87**).

**Figure 17:**
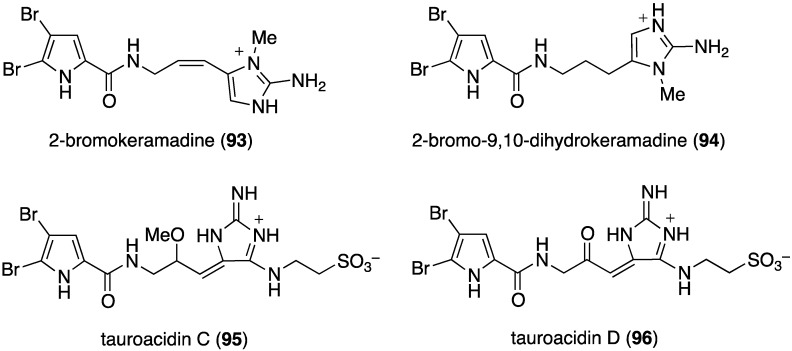

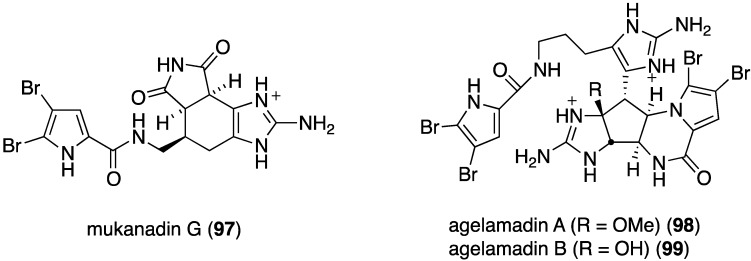
Structures of bromopyrroles **93**–**99** from the sponge *Agelas* sp. (SS-162) [53,54].

**Figure 18:**
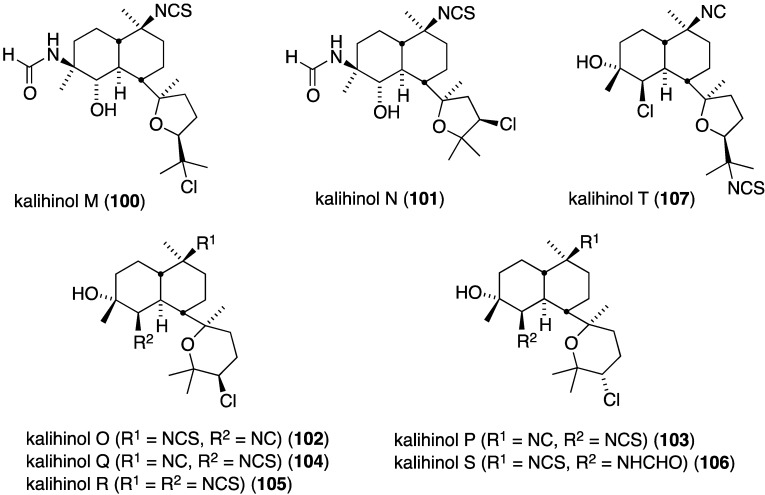
Structures of kalihinols M–T (**100**–**107**) from the sponge *Acanthella caverenosa* [55].

Another examination of the sponge *Agelas* sp. (SS-162) from the Kerama Islands, Okinawa, has led to the isolation of the new bromopyrrole alkaloids, 2-bromokeramadine (**93**), 2-bromo-9,10-dihydrokeramadine (**94**), tauroacidins C (**95**) and D (**96**), and mukanadin G (**97**) (Figure 17) [53]. Of these bromopyrroles, only 2-bromokeramadine (**93**) shows (weak) activity against *E. coli*, although mukanadin G (**97**) has moderate antifungal activity (next section). The highly complex agelamadins A (**98**) and B (**99**) were also characterized in the Okinawan sponge *Agelas* sp. (SS-162) (Figure 18) [54]. Both bromopyrroles are active against *B. subtilis* (MIC, 16 µg/mL each) and *Micrococcus luteus* (MIC, 4.0 and 8.0 µg/mL, respectively). The related agelamadins C–E exhibit only antifungal activity as shown in the next section.

The South China Sea sponge *Acanthella cavernosa* contains eight new chlorinated diterpenoids, kalihinols M–T (**100**–**107**) (Figure 18). In addition, seven previously isolated analogues were isolated [55]. Kalihinols O (**102**), P (**103**), Q (**104**), R (**105**), S (**106**), and T (**107**) exhibit significant antifouling activity against *Balanus amphitrite* larvae: EC_50_ 1.43, 0.72, 1.48, 1.16, 0.53, and 0.74 µM, respectively.

While no new marinopyrroles were reported in the time frame for this review, it is important to cite an excellent survey of these antibacterial marine halogenated pyrroles [56] and an equally excellent report on their activity against methicillin-resistant *S. aureus*, including synthetic marinopyrrole analogues [57].

Like gorgonians and marine sponges, algae employ a chemical arsenal to prevent bacterial smothering (biofouling), and several examples of halogenated antibacterial compounds have been isolated from algae.

**Figure 19:**
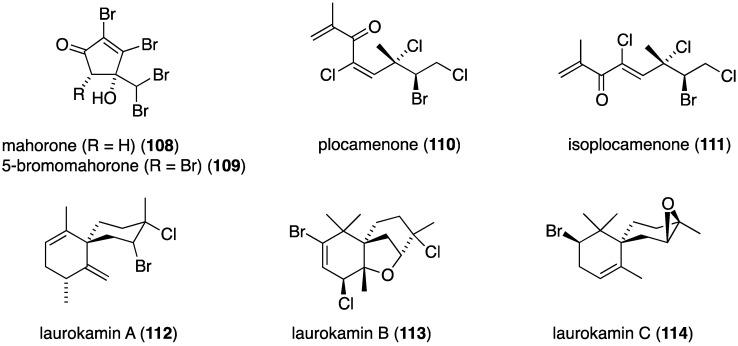
Structures of red algae metabolites **108**–**114** [58,59,60].

The prodigious organohalogen-producing red alga *Asparagopsis taxiformis* “limu kohu,” which is the favorate edible seaweed of native Hawaiians, and the source of more than 100 organohalogens [2,3], contains the unusual mahorone (**108**) and 5-bromomahorone (**109**) (Figure 19) [58]. Both compounds are highly toxic to the marine bacterium *Vibrio fisheri* (EC_50_ 0.16 µM for both), and both are most active against the Gram-negative bacterium *Acinebacter baumanni* and lesser activity towards *E. coli* and *S. aureus*. The red alga *Plocamiun angustum* metabolite plocamenone (**110**) inhibits the growth of *B. subtilis* comparable to that of chloramphenicol (inhibition zone of 10 mm *vs.* 12 mm, respectively). Species of *Laurencia* red algae continue to reveal novel halogenated natural products. A Chinese collection of *Laurencia okamurae* yielded the three new laurokamins A–C (**112**–**114**) (Figure 19) [60], but only laurokamins B (**113**) and C (**114**) show (weak) activity against *E. coli* (6 mm inhibition diameter).

**Figure 20:**
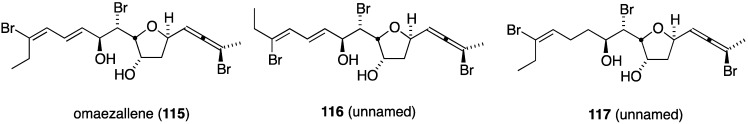
Structures of omaezallenes **115**–**117** from the red alga *Laurencia* sp. [61].

Three omaezallenes (**115**–**117**) were isolated and characterized from a collection of *Laurencia* sp. from Omaezaki, Japan (Figure 20) [61]. Of the three metabolites, omaezallene (**115**) was the most active in an antifouling assay against the larvae of the barnacle *Amphibalanus amphitrite* (EC_50_ 0.22 µg/mL), but only weakly toxic to the larvae (LC_50_ 4.8 µg/mL). The other metabolites have: **116**, EC_50_ 0.30 µg/mL, and **117**, EC_50_ 1.5 µg/mL.

**Figure 21:**
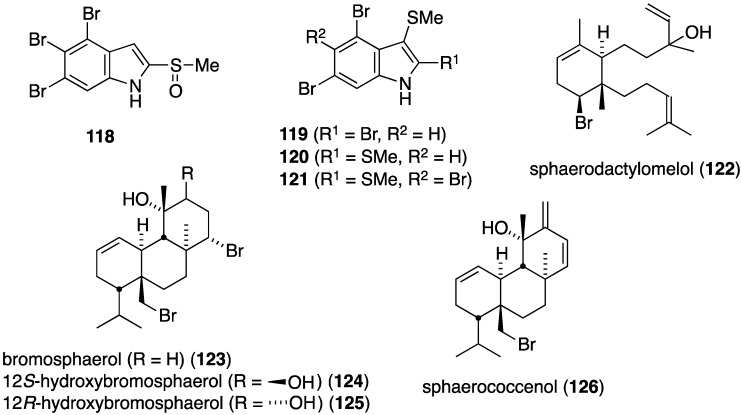
Structures of bromoindoles **118**–**121** and bromosphaerols **122**–**126** [62,63].

A collection of Formosan *Laurencia brongniarii* afforded the new polybrominated indole, 4,5,6-tribromo-2-methylsulfinylindole (**118**) in addition to 11 known brominated indoles (Figure 21) [62]. Although **118** is inactive, of the known indoles, **119**–**121** show significant antibacterial activity against *Enterobacter aerogenes* (ATCC 13048), *Salmonella enteritidis* (ATCC 13076), and *Serratia marcescens* (ATCC 25419). Several bromoditerpenes were characterized from the red alga, *Sphaerococcus coronopifolius*, living in the Berlenga Nature Reserve, Peniche, Portugal. These include the new sphaerodactylomelol (**122**) and the previous known sphaeranes **123**–**126** (Figure 21) [63]. Although no activity against *E. coli* (ATCC 25922) and *Pseudomonas aeruginosa* (ATCC 27853) is observed for **122**–**126**, sphaerodactylomelol (**122**), **123**, and **125** are active against *S. aureus* (IC_50_ 96.30, 22.42, and 6.35 µM, respectively).

**Figure 22:**
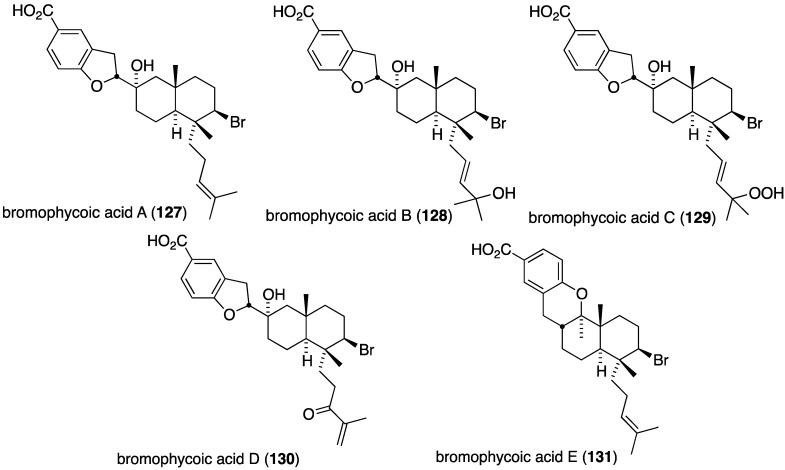
Structures of bromophycoic acids A–E (**127**–**131**) from the red alga *Callophycus* sp. [64].

The Fijian red alga *Callophycus* sp. has yielded five new bromophycoic acids A–E (**127**–**131**) (Figure 22) [64]. These new examples of diterpene-benzoate marine natural products possess a range of biological activities, including antibacterial. For example, all five compounds are active against methicillin-resistant *S. aureus* (MIC 1.6–6.3 µg/mL) with bromophycoic acid A (**127**) being comparable to vancomycin (1.6 *vs.* 2 µg/mL). Likewise, bromophycoic acids A and E are active against vancomycin-resistant *Enterococcus facium* (MIC 6.3 and 1.6 µg/mL, respectively).

**Figure 23:**
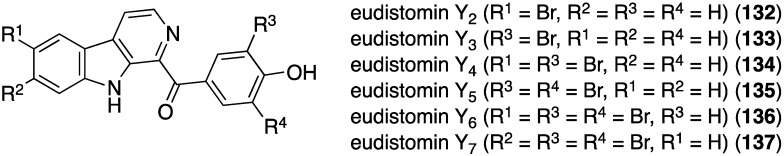
Structures of eudistomins Y_2_–Y_7_ (**132**–**137**) from the ascidian *Synoicum* sp. [65].

The ascidian *Synoicum* sp. collected from Korean waters was found to contain eudistomins Y_2_–Y_7_ (**132**–**137**) (Figure 23) [65]. These known β-carbolines display a range of activity against both Gram-positive and Gram-negative bacteria (Table 3). This study also included the synthesis of several hydroxyl analogues via sodium borohydride reduction of the carbonyl group, but no improvement in antibacterial activity is observed. Although **132**–**137** were previously described, antibacterial activity was not reported [66].

**Figure 24:**
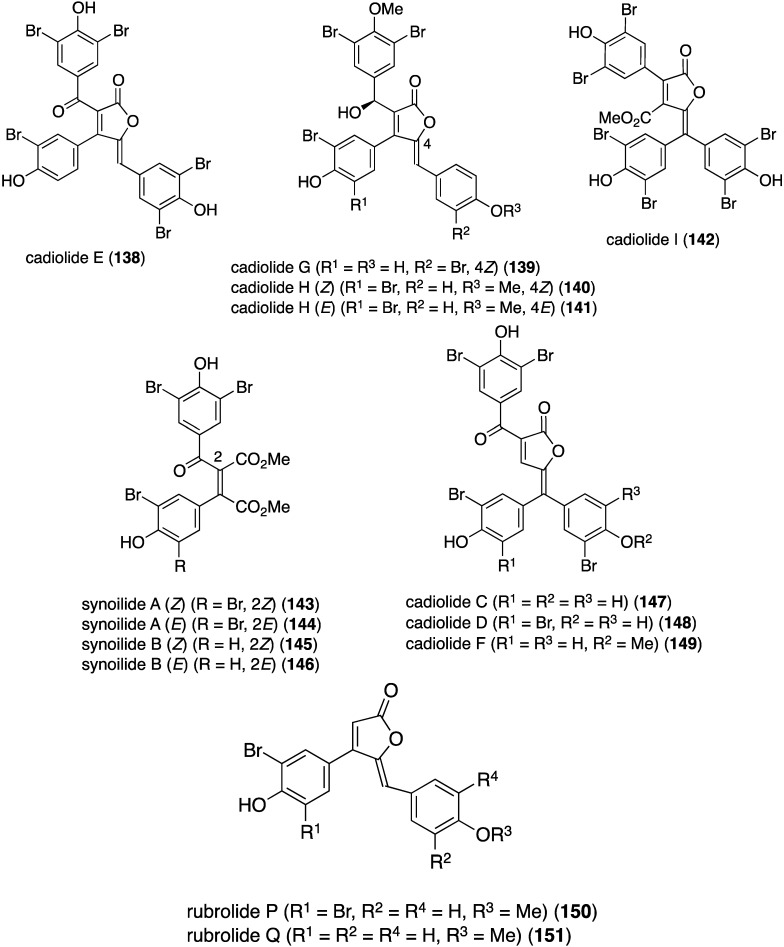
Structures of the cadiolides, synoilides, and rubrolides (**138**–**151**) from the ascidians *Synoicum* and *Pseudodistoma antinboja* [67,68].

Another examination of this ascidian from Korea has revealed the presence of nine new brominated furanones, cadiolides **138**–**142** and synoilides **143**–**146** (Figure 24) [67]. Cadiolides H and synoilides A and B are interconverting *Z* and *E* isomers. Simultaneously with this study, another group isolated cadiolide E (**138**) along with the related cadiolides C (**147**), D (**148**), and F (**149**) from the ascidian *Pseudodistoma antinboja* (Figure 24) [68]. Like the eudistomins (Table 3), the cadiolides display significant antibacterial activity against both Gram-positive and Gram-negative bacteria (Table 4). The synoilides (**143**–**146**) show much weaker or no activity against these bacteria. Cadiolide F (**149**) and rubrolides P (**150**) and Q (**151**) also exist as interconverting *Z* and *E* isomers.

**Table 3:** Antibacterial activity of eudistomins Y_2_–Y_7_ (**132**–**137**) (MIC µg/mL) [65].

Bacterium	Y_2_ (132)	Y_3_ (133)	Y_4_ (134)	Y_5_ (135)	Y_6_ (136)	Y_7_ (137)
*Staphylococcus aureus* (ATCC 6538p)	50	12.5	3.125	6.25	1.56	3.125
*Bacillus subtilis* (ATCC 6633)	25	12.5	0.78	3.125	1.56	0.78
*Micrococcus luteus* (IFO 12708)	25	12.5	1.56	3.125	1.56	1.56
*Salmonella typhimurium* (ATCC 14028)	50	6.25	0.39	0.78	0.39	0.78
*Proteus vulgaris* (ATCC 3851)	25	6.25	0.39	1.56	0.78	0.78
*Escherichia coli* (ATCC 35270)	>100	>100	50	100	50	50

**Figure 25:**
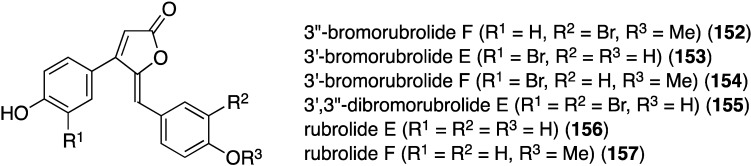
Structures of rubrolides **152**–**157** from the ascidian *Synoicum globosum* [69].

Similar to the cadiolides are the rubrolides and, in addition to rubrolides P (**150**) and Q (**151**), four new examples were found in a South African *Synoicum globosum* ascidian, 3″-bromorubrolide F (**152**), 3′-bromorubrolide E (**153**), 3′-bromorubrolide F (**154**), and 3′,3″-dibromorubrolide E (**155**) (Figure 25) [69]. The previously known non-brominated rubrolides E (**156**) and F (**157**) were also isolated from this animal, and all six rubrolides display varying degrees of antibacterial activity (Table 5). It is noted that 3′-bromorubrolide F (**154**) is identical to rubrolide Q (**151**).

**Table 4:** Antibacterial activity of cadiolides E, G, H, and I (**138**–**142**, **147**–**149**) and rubrolides P and Q (**150**, **151**) (MIC µg/mL) [67,68].

Bacterium	138	139	140/141	142	147	148	149	150	151
*Staphylococcus aureus*	3.1	3.1	6.3	0.8	0.4	6.3	12.5	50	50
*Bacillus subtilis*	1.6	12.5	1.6	0.8	3.1	6.3	12.5	50	50
*Kocuria rhizophilia*	0.8	3.1	3.1	0.8	–	–	–	–	–
*Salmonella enterica*	1.6	0.8	3.1	1.6	–	–	–	–	–
*Proteus hauseri*	3.1	3.1	3.1	6.3	–	–	–	–	–
*Escherichia coli*	>100	>100	>100	>100	–	–	–	–	–
*Staphylococcus epidermidis*	–	–			0.4	0.8	6.3	50	25
*Kocuria rhizophila*	–	–			0.2	1.6	3.1	6.3	3.1

**Table 5:** Antibacterial activity of rubrolides **152**–**157** from the ascidian *Synoicum globosum* (IC_50_ µM) [69].

Bacterium	152	153	154	155	156	157
MRSA ^a^ (ATCC BAA-1720)	256	82	360	89	105	1006
*Staphylococcus epidermidis* (ATCC 35984)	98	38	42	28	21	79
*Enterococcus faecalis* (ATCC 700802) ^b^	43	16	2	2	89	47
*Escherichia coli* (0157:H7) ^b^	22	0	14	25	16	15

^a^ Methicillin-resistant *Staphylococcus aureus*; ^b^ Gentamycin- and vancomycin-resistant; percent growth at 100 µg/mL.

**Figure 26:**
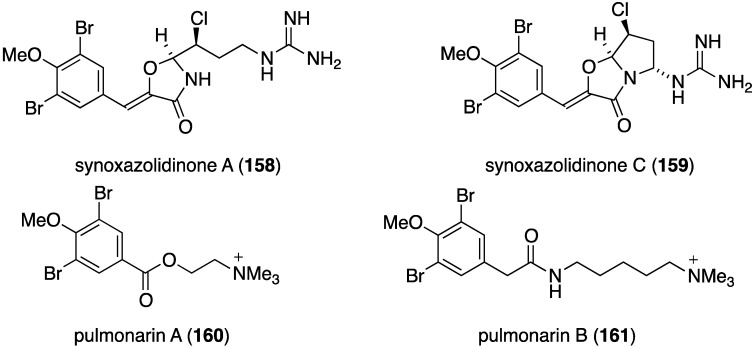
Structures of synoxazolidinones A (**158**) and C (**159**), and pulmonarians A (**160**) and B (**161**) from the ascidian *Synoicum pulmonaria* [70].

The ascidian *Synoicum pulmonaria* from the Norwegian coast contains synoxazolidinones A (**158**) and C (**159**), and pulmonarins A (**160**) and B (**161**) (Figure 26) [70]. The two synoxazolidinones display broad activity against fouling marine species and **159** is comparable to the most active commercial antifouling product, Sea-Nine-211. In contrast, the pulmonarins prevent bacterial growth but have lower activity against microalgae and no activity towards barnacles (Table 6). In addition, several analogues were synthesized, but are generally less active than their natural counterparts.

**Table 6:** Adhesion growth inhibition of synoxazolidiones A (**158**) and C (**159**), and pulmonarin A (**160**) from the ascidian *Synoicum pulmonaria* [70] ^a^.

	156		159		160		161	
	Ad ^b^	Gr ^c^	Ad	Gr	Ad	Gr	Ad	Gr
**Marine Bacteria**								
*Halomonas aquamarina*	20	–	–	2	3	–	–	–
*Polaribacter irgensii*	–	20	20	2	–	0.2	–	–
*Pseudoalteromonas elyakovii*	–	0.02	–	20	–	0.2	–	–
*Roseobacter litoralis*	–	0.02	2	0.2	0.03	–	20	–
*Shewanella putrefaciens*	–	0.2	–	20	–	–	–	–
*Vibrio aestuarians*	–	0.02	2	0.2	0.03	–	20	–
*Vibrio carchariae*	–	2	20	2	3	–	20	–
*Vibrio harveyi*	–	–	2	0.02	–	–	–	–
*Vibrio natriegens*	–	0.02	20	2	0.03	–	20	–
*Vibrio proteolylicus*	–	0.02	2	0.02	–	–	–	–
**Microalgae**								
*Cylindrotheca closterium*	20	20	2	0.2	–	–	–	–
*Exanthemachrysis gayraliae*	20	20	2	0.2	–	–	–	–
*Halamphora coffeaeformis*	20	20	2	2	30	–	–	–
*Pleurochrysis roscoffensis*	20	20	2	2	–	–	–	–
*Porphyridium purpureum*	–	20	0.2	0.02	–	0.2	–	–
**Crustacean Settlement**								
*Balanus improvisus* (IC_50_)	15		2		–		–	

^a^ Inactive at ≥10 µg/mL; ^b^ Adhesion inhibition (MIC, µM); ^c^ Growth inhibition (MIC, µM).

**Figure 27:**
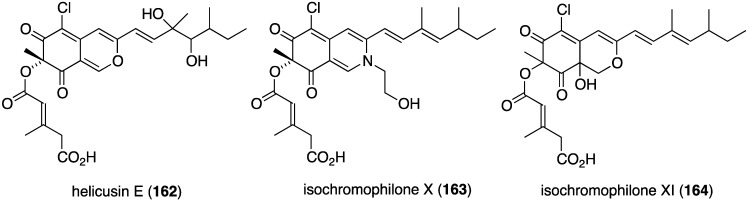
Structures of fungal metabolites **162**–**164** from the fungus *Bartalinia robillardoides* strain LF550 [71].

Several novel antibacterial organohalogen marine fungal metabolites have been discovered in recent years. The fungus *Bartalinia robillardoides* (strain LF550), which was isolated from the Mediterranean sponge *Tethya aurantium*, produces three novel chloroazaphilones, helicusin E (**162**), isochromophilone X (**163**), and isochromophilone XI (**164**) (Figure 27) [71]. Only isochromophilone XI (**164**) shows antibacterial activity against *B. subtilis* (IC_50_ 55.6 µM) and *Staphylococcus lentus* (IC_50_ 78.4 µM), which is slightly less active than the previously known deacetylsclerotiorin, also isolated from this fungus.

**Figure 28:**
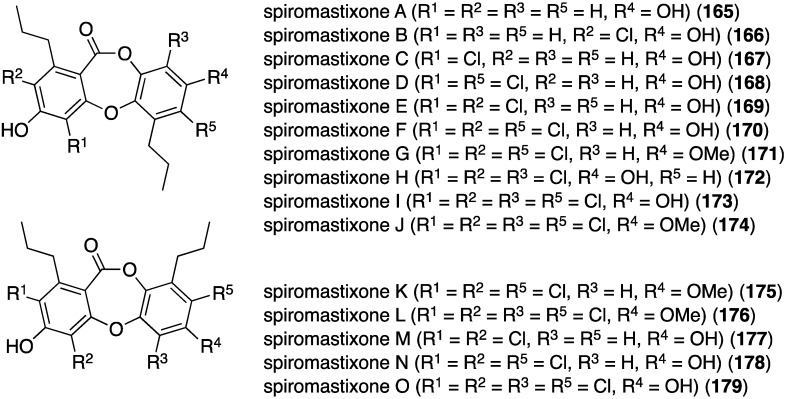
Structures of spiromastixones A–O (**165**–**179**) from the fungus *Spiromastix* sp. [72].

The deep-sea derived *Spiromastix* sp. fungus (collected at 2869 meters) has furnished 15 new spiromastixones A–O (**165**–**179**) (Figure 28) [72]. These novel chlorodepsidones display impressive antibacterial activity against the Gram-positive bacteria *S. aureus* (ATCC 29213), *Bacillus thuringensis* (SCS10 BT01), and *B. subtilis* (SCS10 BT01), but not against the Gram-negative *E. coli* (ATCC 25922). For example, spiromastixone J (**175**) has 0.125, 0.25, and 0.125 µg/mL, respectively, against the three Gram-positive bacteria. Moreover, **175** is strongly inhibitory towards MRSA, methicillin-resistant *Staphylococcus epidermidis* (MRSE), and vancomycin-resistant *Enterococcus faecalis* and *E. faecium* (VSE). Spiromastixones F–I (**171**–**174**) are also potent inhibitors of MRSA and MRSE, and are superior to levofloxacin. This activity increases with an increasing number of chlorines.

**Figure 29:**
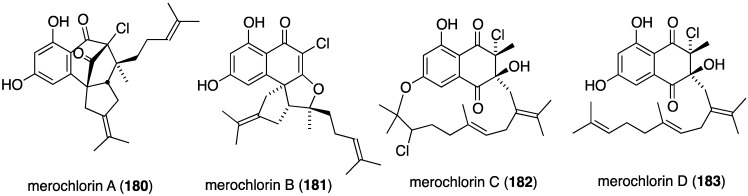
Structures of merochlorins A–D (**180**–**183**) from *Streptomyces* sp. CNH-189 [73,74].

Marine bacteria also produce antibacterial compounds, including those that contain halogen. Merochlorins A–D (**180**–**183**) are novel meroterpenoids isolated from the marine bacterium *Streptomyces* sp. strain CNH-189 from a California coastal sediment (Figure 29) [73,74]. Both merochlorins A (**180**) and B (**181**) are active against MRSA (2–4 µg/mL), and **180** is active *in vitro* against *Clostridium difficile*.

**Figure 30:**
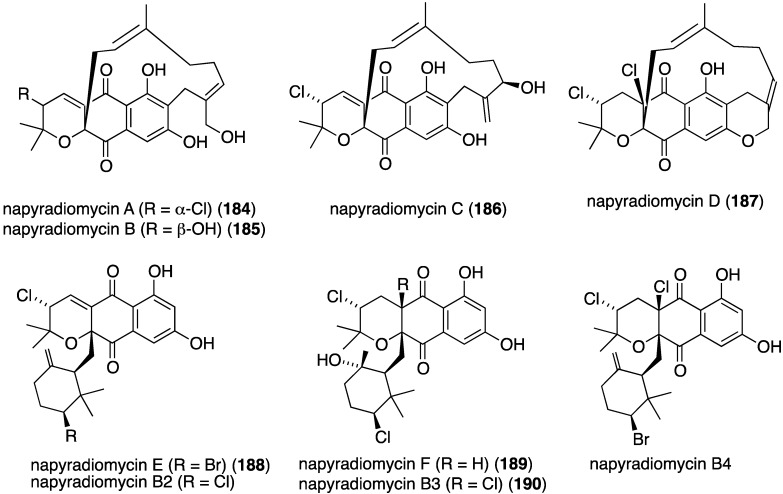
Structures of napyradiomycins A–F (**184**–**189**) from *Streptomyces* CNQ-329 and CNH-070 [75].

Another California marine sediment contains *Streptomyces* strains CNQ-329 and CNH-070, which produce the six novel napyradiomycins A–F (**184**–**189**) (Figure 30) along with three previously known napyradiomycins B2–B4 (e.g., B3 = **190**) [75]. Of these metabolites, napyradiomycins A (**184**) and B3 (**190**) are the most active against MRSA (MIC 16 and 2 µg/mL, respectively).

**Figure 31:**
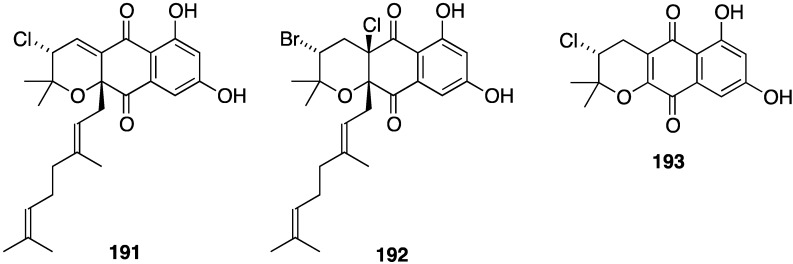
Structures of napyradiomycins **191**–**193** [76].

A Chinese collection of the marine-derived *Streptomyces* sp. SCS10 10428 has afforded the three new napyradiomycins **191**–**193**, in addition to several known analogues, including napyradiomycins B1 and B3 (**190**) (Figure 31) [76]. Metabolites **191** and **192** are strongly active against *S. aureus* ATCC 29213 (MIC 4 and 0.5 µg/mL, respectively), and all three napyradiomycins are active against *B. thuringiensis* SCS10 BT01 and *B. subtilis* SCS10 BS01 (MIC 1–6 µg/mL).

**Figure 32:**
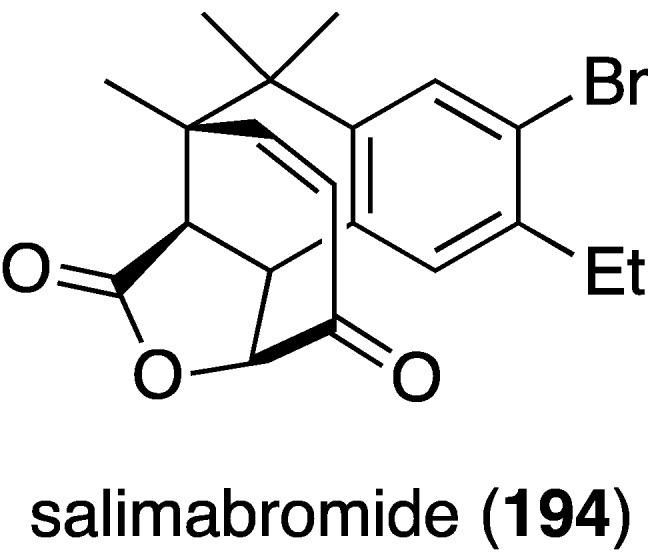
Structure of salimabromide (**194**) from the marine myxobacterium *Enhygromxya salina* [77].

From a coastal sediment in Germany there was isolated the novel salimabromide (**194**), produced by the marine myxobacterium *Enhygromxya salina* (Figure 32) [77]. This structurally unusual compound has modest activity only against *Arthrobacter cristallopoedes*.

**Figure 33:**
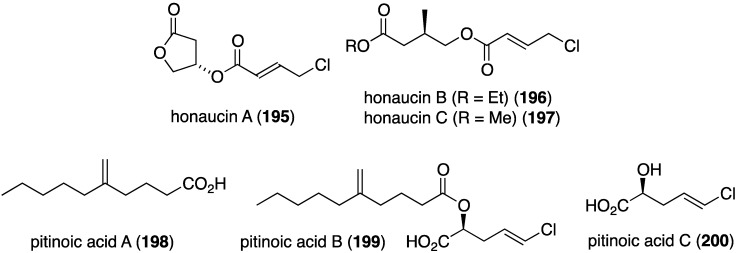
Structures of honaucins A–C (**195**–**197**) from the cyanobacterium *Leptolyngbya crossbyana* [78], and pitinoic acids A–C (**198**–**200**) from a cyanobacterium [79].

Cyanobacteria (blue-green algae) are prodigious producers of biologically active organohalogen natural products, and a collection of *Leptolyngbya crossbyana* found overgrowing on Hawaiian coral yielded the new honaucins A–C (**195**–**197**) (Figure 33) [78]. All three compounds inhibit quorum sensing against *Vibrio harveyi* BB120 (IC_50_ 5.6, 17.6, and 14.6 µM, respectively), and to a lesser extent towards *E. coli* JB525. Interestingly, the synthetic brominated and iodinated analogues of honaucin A (**195**) are more active in quorum sensing inhibition than the natural honaucin A itself. A Guamanian cyanobacterium which is very similar to *Lyngbya* produces the novel biologically active lipids pitinoic acids A–C (**198**–**200**), which inhibit quorum sensing in the Gram-negative bacterium *Pseudomonas aeruginosa* (Figure 33) [79].

## 3. Antifungal Activity

**Figure 34:**
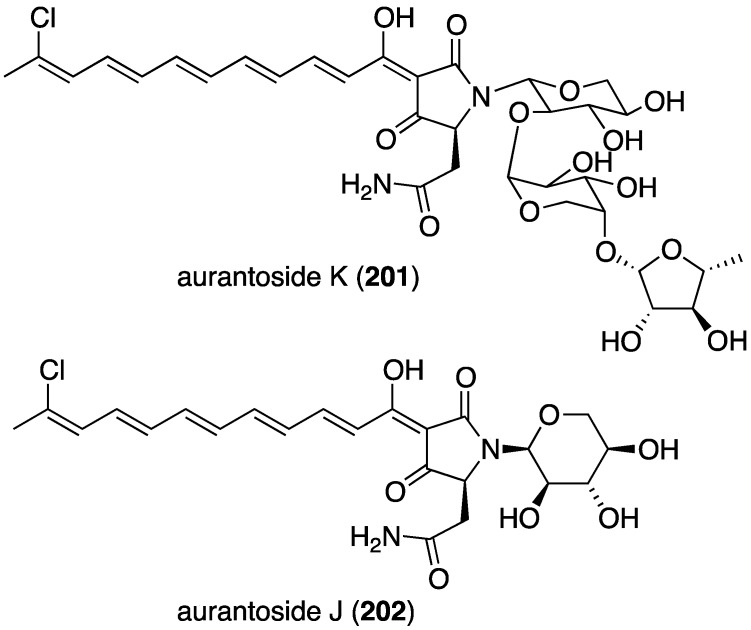
Structure of aurantoside K (**201**) from the sponge *Melophlus* sp. [80] and aurantoside J (**202**) from the sponge *Theonella swinhoei* [81].

In addition to their often potent antibacterial activity (*vide supra*), many marine sponges contain halogenated metabolites with powerful antifungal properties. The new tetramic acid glycoside, aurantoside K (**201**), was isolated from a Fijian sponge belonging to the genus *Melophlus* (Figure 34) [80]. Auranotoside K is a demethylated analogue of the previously known aurantoside I. Although devoid of antibacterial, antimalarial, and cytotoxicity in the assays examined, **201** displays broad antifungal activity towards *Candida albicans* (wild type ATCC 32354 and amphotericin-resistant ATCC 90873; MIC 31.25 and 1.95 µg/mL, respectively), *Cryptococcus neoformans*, *Aspergillus niger*, *Penicillium* sp., *Rhizopus sporangia*, and *Sordaria* sp. The Indonesian sponge *Theonella swinhoei* has yielded the new aurantoside J (**202**), which is an epimer of the previously known auranotoside G (Figure 34) [81]. The new **202** differs from aurantoside G at the anomeric center C-1′ of the xylose sugar unit. Antifungal activity of **202** is negligible compared to that of aurantosides G and I.

**Figure 35:**
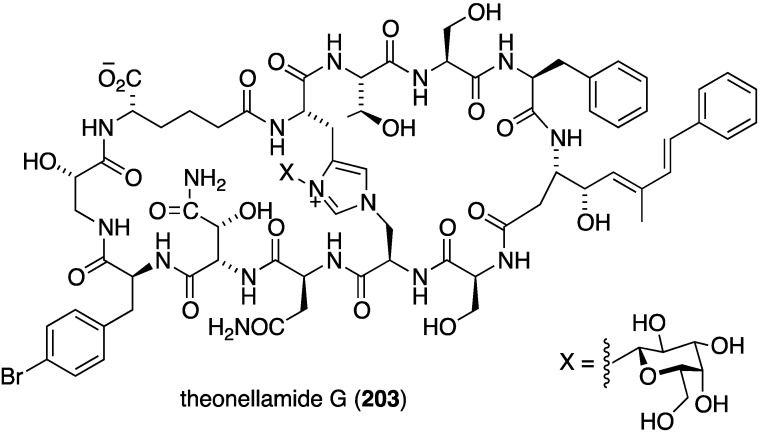
Structure of theonellamide G (**203**) from the sponge *Theonella swinhoei* [82].

A Red Sea specimen of *Theonella swinhoei* contains the antifungal glycopeptide theonellamide G (**203**) (Figure 35), which is very similar to the known theonellamide A, lacking only a methyl group on the *p*-bromophenylalanine and a hydroxyl group in the α-aminoadipic acid group [82]. Theonellamide G shows potent antifungal activity against both wild and amphotericin B-resistant strains of *Candida albicans*; IC_50_ 4.49 and 2.0 µM, respectively. The positive control amphotericin B had 1.48 µM against the wild type *Candida albicans*.

**Figure 36:**
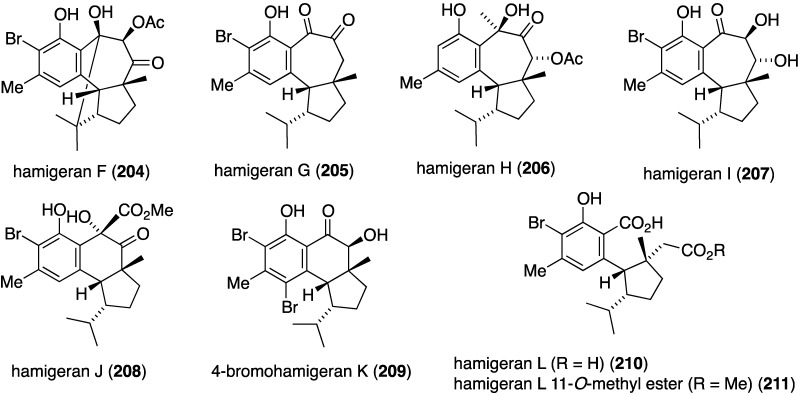
Structures of hamigerans **204**–**211** from the sponge *Hamigera tarangaensis* [83].

The New Zealand sponge *Hamigera tarangaensis* has yielded a suite of new hamigerans (**204**–**211**) (Figure 36), in addition to several known related hamigerans [83]. Hamigeran G (**205**) also exists as an enol tautomer, and hamigeran F (**204**) undergoes what appears to be an acid-catalyzed retro-aldol transformation (observed in a CDCl_3_ solution of **204**). Hamigeran G selectively inhibits the growth of two strains of the yeast *Saccharomyces cerevisiae*.

**Figure 37:**
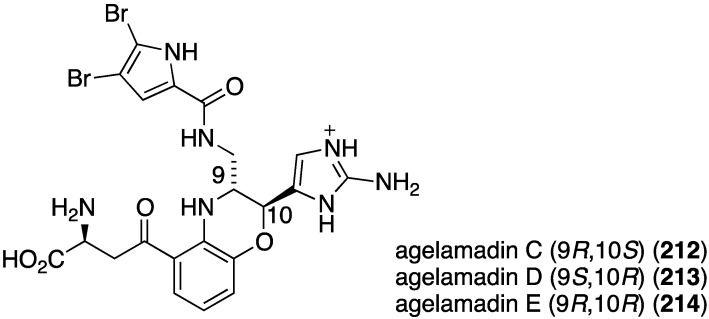
Structures of agelamadins C–E (**212**–**214**) from the sponge *Agelas* sp. SS-162 [84].

The indolo[3,2-*a*]carbazole **72** from the deep-water sponge *Asteropus* sp. is antifungal towards *Candida albicans* (MIC 25 µg/mL), but **73** is not [48]. Similarly, purpuroine D (**61**) is active against *C. albicans* (IC_50_ 19.03 ± 0.12 µg/mL), and purpuroines A (**59**), C (**60**), and D (**61**) inhibit the human disease-causing *Aspergillus fumigates* (IC_50_ 28.58 ± 0.52, 26.07 ± 0.55, 25.56 ± 0.44 µg/mL, respectively) [46]. The previously cited nakijinamine A (**79**) shows antifungal activity towards *C. albicans* (IC_50_ 0.25 µg/mL), *Cryptococcus neoformans* (IC_50_ 0.5 µg/mL), and *Trichophyton mentagrophytes* (IC_50_ 0.25 µg/mL). Less activity against *C. albicans* is seen with nakijinamines B (**80**) and F (**81**) (IC_50_ 8 µg/mL each) [50]. The *Agelas* sponge metabolites, agelasines O (**86**), P (**87**), Q (**88**), R (**89**), and T (**91**) show varying degrees of activity against the fungi *C. albicans*, *Aspergillus niger*, *Trichophyton mentagrophytes*, and *Cryptococcus neoformans*, with the greatest activity towards the latter fungus by Q (**88**) and R (**89**) (IC_50_ 8.0 µg/mL each) [52]. Similarly, these four fungi species are inhibited by the *Agelas* bromopyrroles **93**–**97**, especially mukanadin G (**97**) against *C. albicans* and *Cryptococcus neoformans* (IC_50_ 16 and 8.0 µg/mL, respectively) [53]. In addition to the *Agelas* sp. sponge metabolites agelamadins A (**98**) and B (**99**) [54], the new agelamadins C–E (**212**–**214**) (Figure 37) are also present in this sponge [84]. Antifungal activity is displayed against *Cryptococcus neoformans* by agelamadins A (**98**), B (**99**), C (**212**), and E (**214**) (IC_50_ 8.0, 4.0, 32, 32 µg/mL, respectively [54,84].

**Figure 38:**
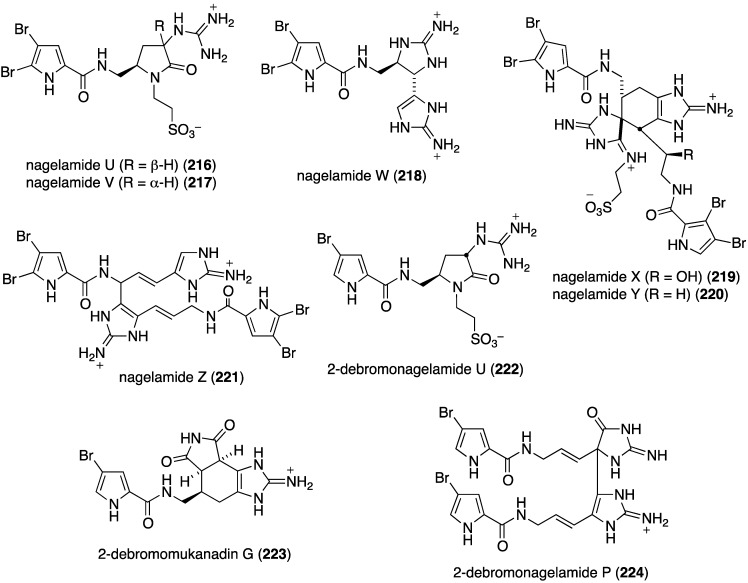
Structures of nagelamides U–Z (**216**–**221**) and **222**–**224** from *Agelas* spp. sponges [85,86,87].

Further examination of *Agelas* spp. (SS-162 and SS-156) sponges from Okinawa reveals the presence of nagelamides U–W (**216**–**218**) [85], X–Z (**219**–**221**) [86], 2-debromonagelamide U (**222**), 2-debromomukanadin (**223**), and 2-debromonagelamide P (**224**) [87] (Figure 38). Antifungal activity against several fungi is summarized in Table 7, for which nagelamide Z (**221**) shows significant activity towards all four fungi.

**Figure 39:**
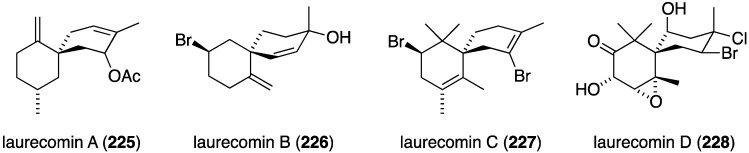
Structures of laurecomins A–D (**225**–**228**) from the red alga *Laurencia composita* [88].

Marine algae can exhibit antifungal activity and several recent examples are described. The red alga *Laurencia composita*, collected from Pingtan Island, China, has afforded novel chamigranes, the laurecomins A–D (**225**–**228**) (Figure 39) [88]. Of these, laurecomin B (**226**) is antifungal towards *Colletotrichum lagenarium* (inhibitory diameter of 10 mm).

**Figure 40:**
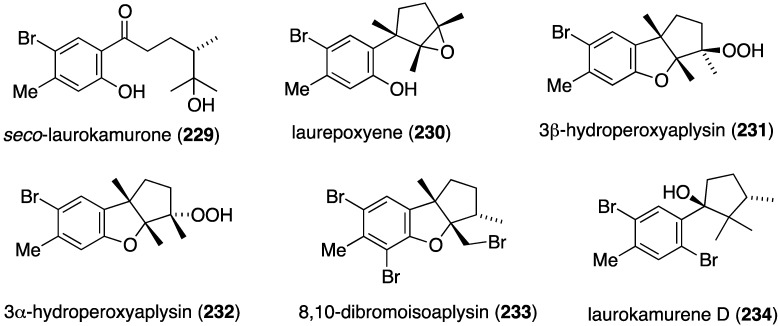
Structures of brominated sesquiterpenes **229**–**234** from the red alga *Laurencia okamurai* [89].

A collection of *Laurencia okamurai* from Nanji Island, China, has furnished several new brominated sesquiterpenes, *seco*-laurokamurone (**229**), laurepoxyene (**230**), 3β-hydroperoxyaplysin (**231**), 3α-hydroperoxy-3-epiaplysin (**232**), 8,10-dibromoisoaplysin (**233**), and laurokamurene D (**234**) (Figure 40) [89]. Antifungal activity of **230**–**233** is tabulated in Table 8.

**Table 7:** Antifungal activity of nagelamides U–Z (**216**–**221**) and **222**–**224** [85,86,87].

	Compound (IC_50_ µg/mL)							
Fungus	216	218	219	220	221	222	223	224
*Candida albicans*	4	4	2.0	2.0	0.25	–	–	–
*Trichophyton mentagrophytes*	–	–	16	<32	4.0	16	–	32
*Cryptococcus neoformans*	–	–	<32	<32	2.0	32	32	–
*Aspergillus niger*	–	–	32	<32	4.0	–	–	–

**Table 8:** Antifungal activity of brominated sesquiterpenes **230**–**233** from the red alga *Laurencia okamurai* [89].

	Compound ^a^					
Fungus	230	231	232	233	Amphotericin B ^b^	Fluconazole ^b^
*Cryptococcus neoformans* (32609)	>64	4	8	>64	1	1
*Candida glabrata* (537)	2	4	>64	>64	2	1
*Trichophyton rubrum*	32	16	>64	>64	1	>64
*Aspergillus fumigatus* (07544)	>64	>64	>64	>64	2	8

^a^ MIC_50_ µg/mL; ^b^ Positive controls.

**Figure 41:**
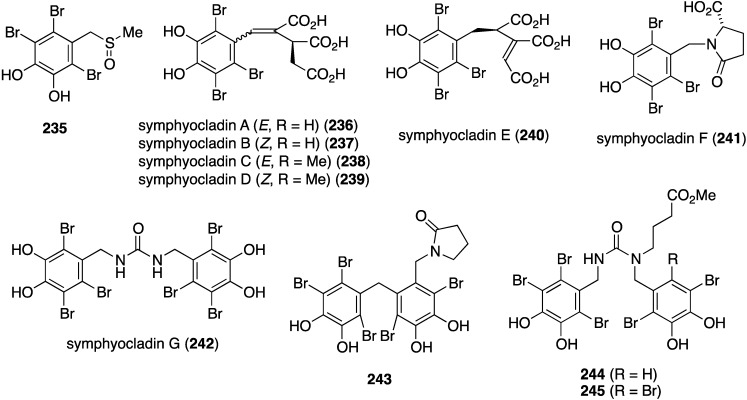
Structures of symphyocladins A–G (**236**–**242**) and other bromophenols from the red alga *Symphyocladia latiuscula* [90,91,92].

The red alga *Symphyocladia latiuscula* from the coast of Qingdao, China, is a rich source of brominated phenols, and several new examples have been discovered (**235**–**245**) (Figure 41) [90,91,92]. Bromocatechols **235**, **242**, and **244** display moderate activity against *Candida albicans* (MIC 37.5, 10, and 25 µg/mL, respectively) [90,91,92].

**Figure 42:**
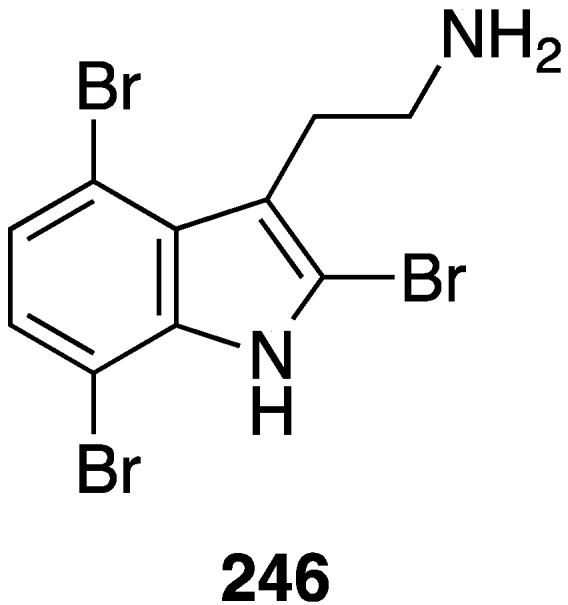
Structure of 2,4,7-tribromotryptamine (**246**) from the bryozoan *Chartella membranaceatruncata* [93].

Weak antifungal activity is observed for gemmacolides T–Y (**7**–**12**) against *Microbotryum violaceum* and *Septoria tritici*, in the zone of inhibition ranging from 9.5–17 mm [31]. Two of the *Synoicum* sp. ascidian eudistomins, Y_2_ (**132**) and the non-brominated Y_1_, show potent to moderate activity against *Candida albicans* (MIC 6.25 and 50 µg/mL, respectively) [65]. The other eudistomins Y_3_–Y_7_ are inactive against the four fungal strains tested. A study of the bryozoan *Chartella membranaceatruncata*, collected in Kandalaksha Bay, the White Sea, resulted in the characterization of 2,4,7-tribromotryptamine (**246**) (Figure 42), which displays potent activity towards *Candida albicans* and *Saccharomyces cereviseae*, although this result was not quantified [93].

Several marine-derived bacteria have antifungal properties, such as strepchloritide B (**47**), from *Streptomyces* sp. OUCMDZ-1703, towards *Candida albicans* (13 ± 0.5 mm inhibitory diameter zone) [42], and the extraordinarily complex forazoline A (**247**), from *Actinomadura* sp. cultivated from the ascidian *Ecteinascidia turbinata*, towards *Candida albicans* (MIC 16 µg/mL) [94]. This unique marine polyketide is also active *in vivo* in a disseminated candidiasis model in mice, with no toxicity. This important antifungal compound may prove to be a clinical candidate to treat *Candida albicans* fungal infections in humans such as candidiasis, which affects some 400,000 people annually with a mortality rate of 46%–75% [95]. Indeed, fungal infections of all types cause 1.5 million deaths per year worldwide [96].

**Figure 43:**
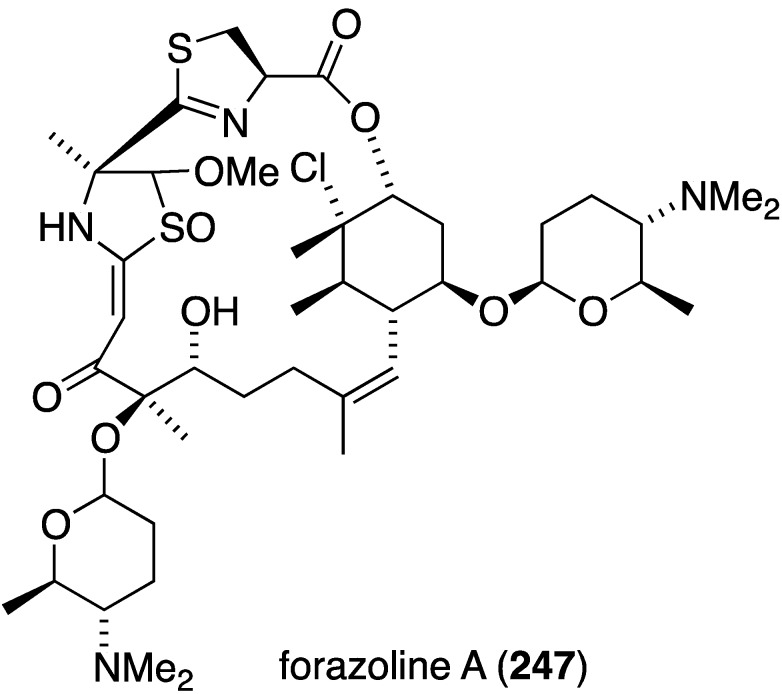
Structure of forazoline A (**247**) from *Actinomadura* sp. [94].

**Figure 44:**
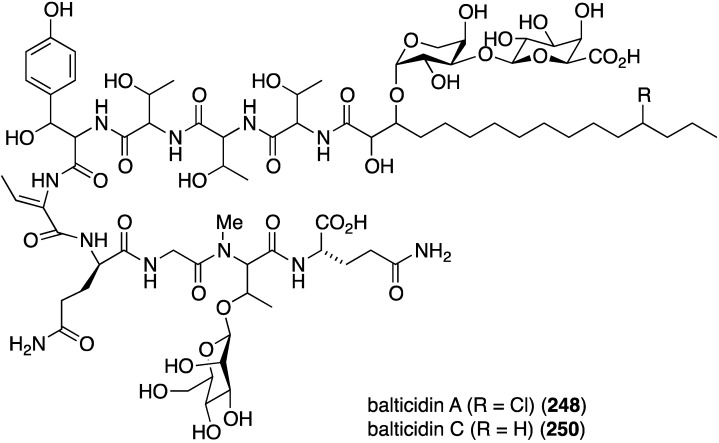

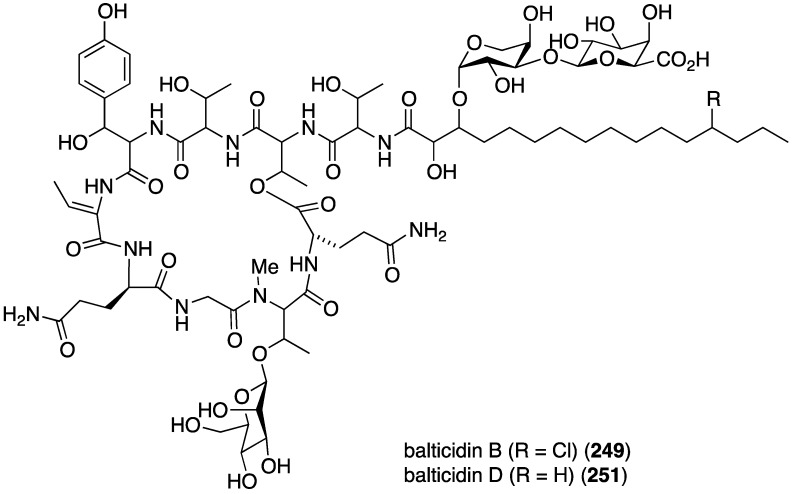
Structures of balticidins A–D (**248**–**251**) from the cyanobacterium *Anabaena cylindrica* Bio33 [97,98].

The fungal metabolite isochromophilone XI (**164**), from *Bartalinia robillardoides*, is active against the fungus *Trichophyton rubrum* (IC_50_ 41.5 µM), but not against *Candida albicans* and *Septoria tritici* [71]. The Baltic Sea cyanobacterium *Anabaena cylindrica* Bio33, cultivated in the laboratory, has provided the antifungal lipopeptides balticidins A–D (**248**–**251**) (Figure 44) [97,98]. These complex metabolites are active towards *Candida maltosa* with inhibition zones for balticidins A–D of 12, 15, 9, and 18 mm, respectively [97]. Antifungal activity with these compounds is also observed against *C. albicans*, *Candida krusei*, *Aspergillus fumigatus*, *Microsporum gypseum*, *Mucor* sp., and *Microsporum canis*. No antibacterial activity is observed for these compounds.

## 4. Antiparasitic Activity

The parasitic diseases malaria, leishmaniasis, echinococcus, Chagas disease (American trypanosomiasis), onchocerciasis (“river blindness”), dracunculiasis (“guinea worm disease”), trypanosomiasis (“sleeping sickness”), schistosomiasis, lymphatic filariasis (elephantiasis), and others in tropical and sub-tropical regions put billions of people at risk and account for millions of illnesses and deaths annually [99,100,101,102,103,104]. The need for new drugs to combat these parasite vectors is paramount, and a number of newly isolated halogenated marine natural products display antiparasitic activity.

**Figure 45:**
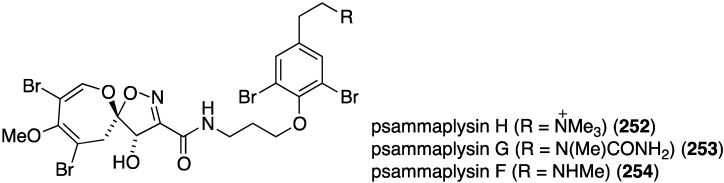
Structures of psammaplysins H (**252**), G (**253**), and F (**254**) from the sponge *Pseudoceratina* sp. [104].

A study of the Australian sponge *Pseudoceratina* sp. has yielded the new psammaplysin H (**252**) in addition to the known psammaplysins G (**253**) and F (**254**) (Figure 45) [104]. Compared to the latter two metabolites, **252** displays potent *in vitro* antimalarial activity against the chloroquine-sensitive (3D7) line of *Plasmodium falciparum* (IC_50_ (µM), **252**: 0.41 ± 0.1; **253**: 5.22 ± 1.6; **254**: 1.92 ± 1.1), and minimal toxicity towards the mammalian cell lines HEK293 and HepG2, relative to **253** and **254** (selectivity index ≥97).

**Figure 46:**
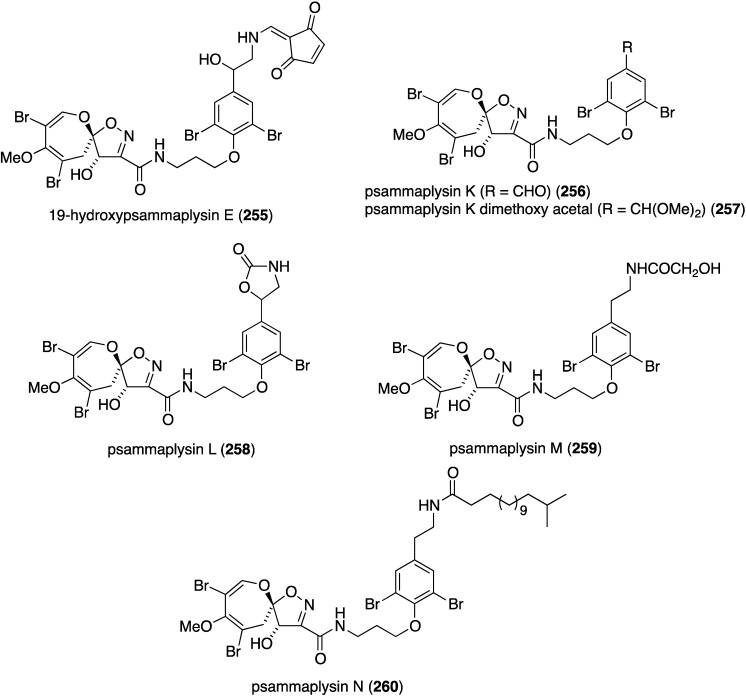

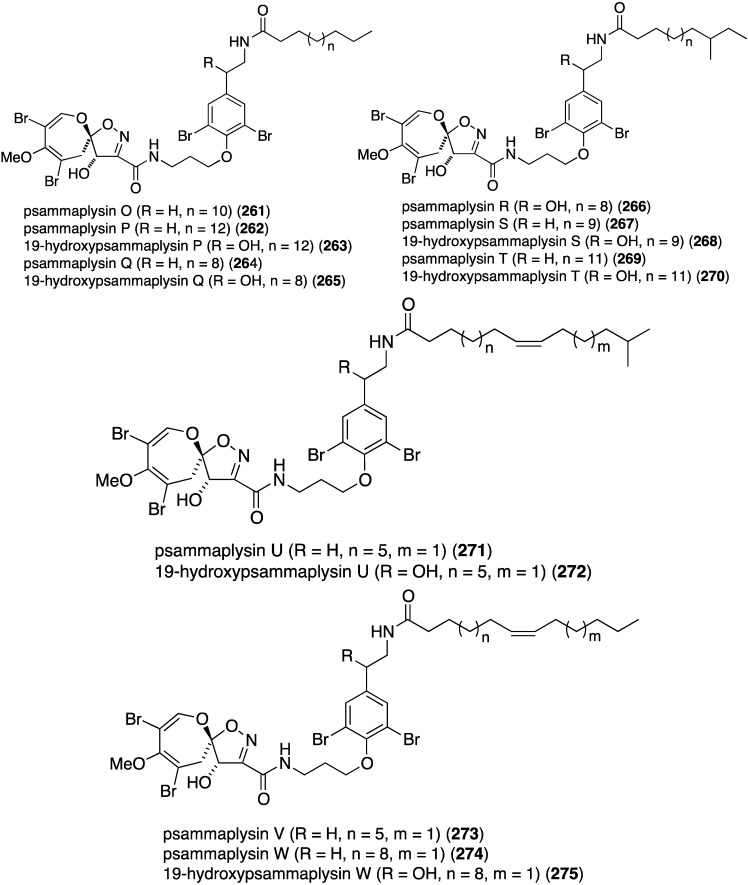
Structures of psammaplysins **255**–**275** from the sponge *Aplysinella strongylata* [105].

The Balinese sponge *Aplysinella strongylata* is the repository of 21 new psammaplysins (**255**–**275**) and six previously known analogues (Figure 46) [105]. Of those compounds tested (**255**, **256**, **258**, **259**, **263**, **269**, and **273**) against the 3D7 *Plasmodium falciparum* parasite, 19-hydroxypsammaplysin E (**255**) displays the highest activity (IC_50_ 6.4 µM).

**Figure 47:**
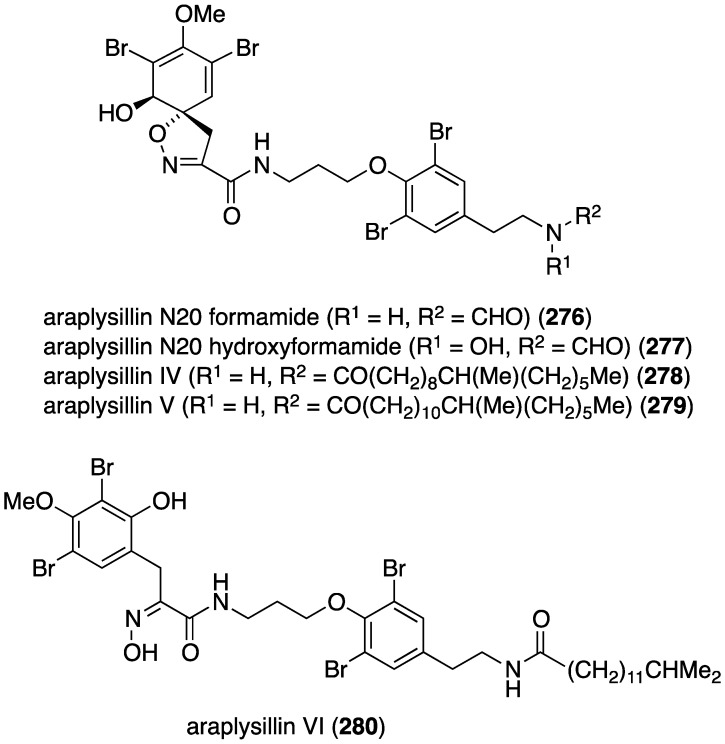
Structures of araplysillins **276**–**280** from the sponge *Suberea ianthelliformis* [106].

A collection of the sponge *Suberea ianthelliformis* has yielded five new bromotyrosines, araplysillin N20 formamide (**276**), araplysillin N20 formamide *N*-oxide (**277**), and araplysillins IV–VI (**278**–**280**) (Figure 47) in addition to 13 known brominated analogues [106]. Of the new metabolites, **276** and **277** show moderate activity against both chloroquine-resistent (FcB-1) strain of *Plasmodium falciparum* (IC_50_ 3.6 and 5.0 µM, respectively) and the chloroquine-sensitive (3D7) strain (IC_50_ 7.0 and 4.1 µM, respectively).

**Figure 48:**
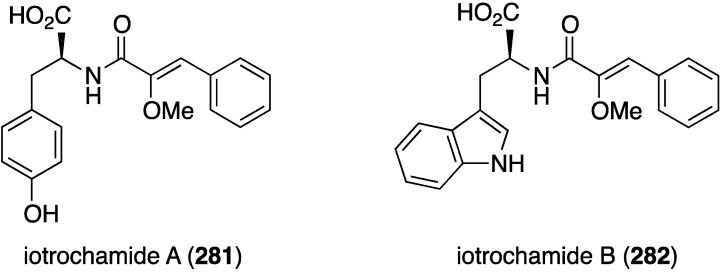
Structures of iotrochamides A (**281**) and B (**282**) from the sponge *Iotrochota* sp. [108].

A specimen of *Verongula rigida* from the coast of Columbia has afforded nine previously known bromotyrosines, and two of these, purealidin B and 11-hydroxyaerothionin, display selective antiparasitic activity at 10 and 5 µM against *Leishmania panamensis* and *Plasmodium falciparum* parasites, respectively [107]. The Australian sponge *Iotrochota* sp. contains the two antitrypanosomal compounds, iotrochamides A (**281**) and B (**282**) (Figure 48). Both compounds exhibit moderate activity against *Trypanosoma brucei brucei* (IC_50_ 3.4 and 4.7 µM, respectively) [108].

**Figure 49:**
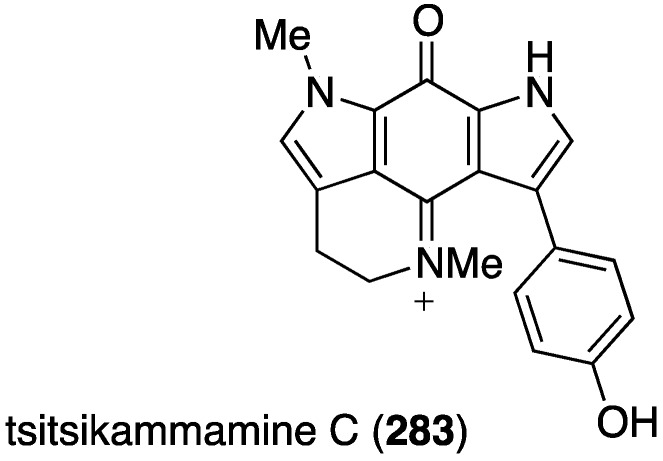
Structure of tsitsikammamine C (**283**) from the sponge *Zyzzya* sp. [109].

Another Australian marine sponge, *Zyzzya* sp., has furnished the new tsitsikammamine C (**283**), along with six previously known structurally related brominated alkaloids (Figure 49) [109]. This novel bispyrroloiminoquinone displays extraordinarily potent *in vitro* antimalarial activity towards both chloroquine-sensitive (3D7) and chloroquine-resistant (Dd2) *Plasmodium falciparum* with values of IC_50_ 13 and 18 nM, respectively. The selectivity index against HEK293 cells is >200. Known alkaloids makaluvamines J, G, and L are slightly less active than tsitsikammamine C in both screens.

**Figure 50:**
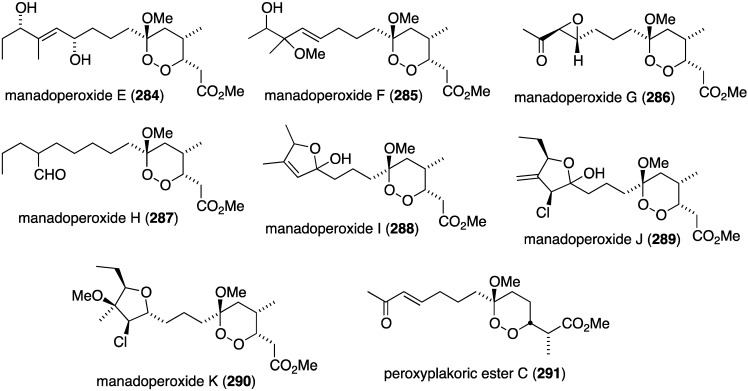
Structures of manadoperoxides E–K (**284**–**290**) and peroxyplakoric ester C (**291**) from the sponge *Plakortis* cfr. *lita* [110].

A suite of new manadoperoxides E–K (**284**–**290**) and peroxyplakoric ester C (**291**) were isolated from the sponge *Plakortis* cfr. *lita* (Figure 50), two of which contain chlorine (**289**, **290**), in addition to several known manadoperoxides [110]. Manadoperoxides I (**288**) and K (**290**) display the greatest activity of the new compounds tested against *Trypanosoma brucei rhodesiense* (IC_50_ 0.062 and 0.087 µg/mL, respectively) and *Leishmania donovani* (IC_50_ 0.633 and 1.89 µg/mL, respectively). However, the known manadoperoxide B has IC_50_ values of 0.003 and 0.589 µg/mL, respectively.

**Figure 51:**
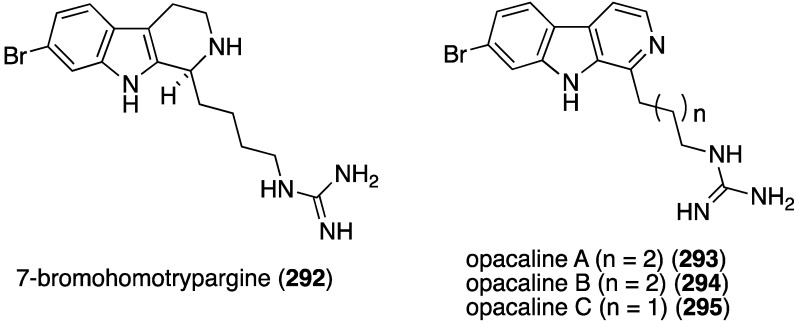
Structures of **292** and opacalines A–C (**293**–**295**) from the ascidian *Pseudodistoma opacum* [112].

The previously described bromophycoic acids (**127**–**131**) display activity against the malaria parasite (3D7 strain) *Plasmonium falciparum*, with the peroxy bromophycoic acid C (**129**) being the most active: IC_50_ 8.7 µM [64]. For comparison, the known macrolide bromophycolide A shows IC_50_ 0.5 µM [111] and the positive controls chloroquine and artemisin show IC_50_ 0.0058 and 0.0062 µM, respectively [64]. The New Zealand ascidian *Pseudodistoma opacum* contains four new brominated β-carboline alkaloids, (–)-7-bromohomotrypargine (**292**) and opacalines A–C (**293**–**295**) (Figure 51) [112]. Opacalines B and C show activity against several parasites (Table 9). Some non-brominated synthetic analogues have comparable antiparasitic activity. For example, debromoopacaline C shows IC_50_ 7.7 µM against *Trypanosoma brucei rhodesiense*, a parasite that causes human African trypanosomiasis.

**Table 9:** Antiparasitic activity of opacalines B (**294**) and C (**295**) and positive controls (IC_50_ µM).

	Compound					
Parasite	294	295	Melarsoprol	Benznidazole	Miltefosine	Chloroquine
*Trypanosoma brucei rhodesiense*	30	27	0.005	–	–	–
*Trypanosoma cruzi*	86	107	–	1.8	–	–
*Leishmania donovani*	130	101	–	–	0.53	–
*Plasmodium falciparum*	2.5	4.5	–	–	–	0.28

**Figure 52:**
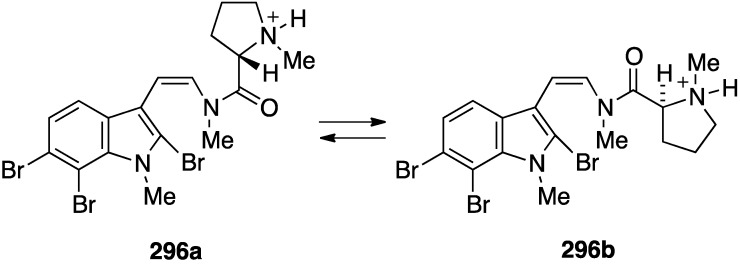
Structure of kororamide A (**296**) from the bryozoan *Amathia tortuosa* [113].

The novel kororamide A (**296**) from the Australian bryozoan *Amathia tortuosa*, which exists as a mixture of interconverting amide rotamers (Figure 52), has activity against *Plasmodium falciparum*; 70% growth inhibition at 20 µM for the chloroquine-sensitive strain, but only 50% growth inhibition of 50% at 20 µM for the chloroquine resistant strain [113].

**Figure 53:**
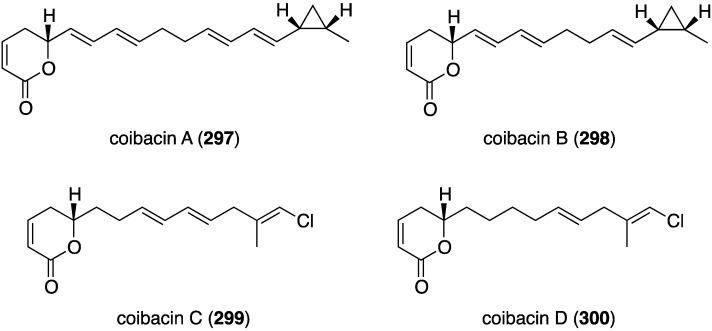
Structures of coibacins A–D (**297**–**300**) from the cyanobacterium *Oscillatoria* sp. [114].

A Panamanian cyanobacterium, *Oscillatoria* sp., has afforded the novel polyketide lactones coibacins A–D (**297**–**300**) (Figure 53) [114]. Activity of these compounds is seen against the parasite *Leishmania donovani* axenic amastigotes, with coibacin A (**297**) being the most active, showing IC_50_ 2.4 µM. The coibacins were inactive to malaria and Chagas’ disease.

## 5. Antiviral Compounds

In addition to harmful bacteria, fungi, and parasites, humans have to contend with lethal viruses, and the search for new antiviral compounds is intense. Although fewer in number than terrestrial sources, the marine environment has produced some antiviral active compounds. A review of antiviral lead compounds from sponges has appeared [115].

A collection of 11 well-known bromotyrosines that was isolated from the Columbian sponges *Verongula rigida* [107,116] and *Aiolochoria crassa* [117] were examined for *in vitro* inhibition of HIV-1 replication [118]. Of these 11, six inhibit HIV-1 replication at different steps. Aeroplysinin-1, purealidin B, and 3-bromo-5-hydroxy-*O*-methyltyrosine inhibit the HIV-1 replication in a dose-dependent fashion, with a median maximum inhibition percentage of 74% at 20 µM and 47% at 80 µM (not cytotoxic at these concentrations). Aeroplysinin-1, 19-deoxyfistularin 3, purealidin B, fistularin 3, and 3-bromo-5-hydroxy-*O*-methyltyrosine efficiently inhibit the nuclear import. In addition, aeroplysinin-1, purealidin B, fistularin 3, 3-bromo-5-hydroxy-*O*-methyltyrosine, and 3,5-dibromo-*N*,*N*,*O*,*O*-tetramethyltyraminium inhibit X4 HIV-1 cell entry with an inhibition median percentage maximum of 2%–30% [118].

**Figure 54:**
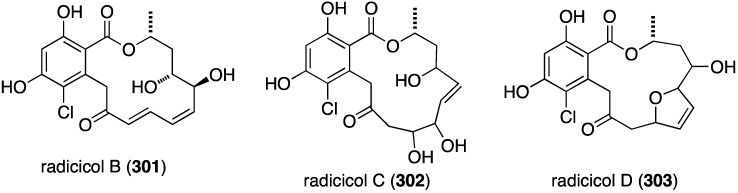
Structures of radicicols B–D (**301**–**303**) from *Humicola fuscoatra* [119].

The marine-derived fungus *Humicola fuscoatra* has yielded three new chlorine-containing resorcyclic acid lactones, radicicols B–D (**301**–**303**) (Figure 54), in addition to the known radicicol (=monorden) and pochonins B, D, and N [119]. Of the three new radicicols, radicicol B (**301**) is the most active in the latent HIV-1 reactivation assay (25%; EC_50_ 24.9 µM), but inferior to radicicol (98%; EC_50_ 9.1 µM), pochonin B (98%; EC_50_ 39.6 µM), and pochonin C (92%; EC_50_ 6.3 µM). Those compounds lacking a conjugated carbonyl (Michael acceptor) are inactive.

**Figure 55:**
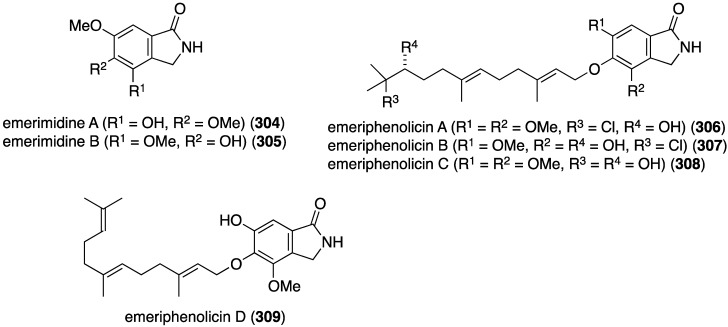
Structures of emerimidines A (**304**) and B (**305**), and emeriphenolicins A–D (**306**–**309**) [121].

Mangrove plants that grow in tropical and subtropical intertidal estuarine zones are a rich source of natural products including organohalogens [120]. The Chinese mangrove plant *Aegicerus corniculatum* has an associated fungus, *Emericella* sp., that produces six new isoindolones, emerimidines A (**304**) and B (**305**), and emeriphenolicins A–D (**306**–**309**) (Figure 55) [121]. Of this collection only the non-halogenated emerimidines A and B display antiviral activity towards influenza A virus (H_1_N_1_) replication in MDCK cells (IC_50_ 42.07 and 62.05 µg/mL, respectively; ribavirin positive control: 24.60 µg/mL).

**Figure 56:**
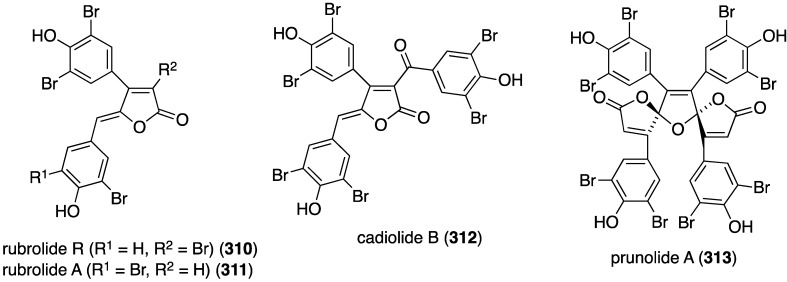
Structures of **310**–**313** from the ascidian *Synoicum* sp. [122].

The Indian Ocean ascidian *Synoicum* sp. has yielded the new rubrolide R (**310**) and the known rubrolide A (**311**) (isolated as the diacetates), along with the known cadiolide B (**312**) and prunolide A (**313**) (Figure 56) [122]. The latter two metabolites are active against the Japanese encephalitis virus at a concentration of 1 µg/mL.

**Figure 57:**
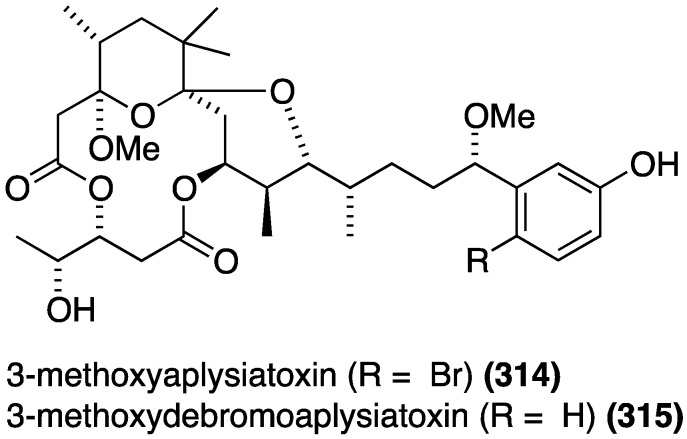
Structures of aplysiatoxins **314** and **315** from the cyanobacterium *Trichodesmium erythraeum* [123].

The marine cyanobacterium *Trichodesmium erythraeum*, collected in Singapore, has afforded the new aplysiatoxins, 3-methoxyaplysiatoxin (**314**) and 3-methoxydebromoaplysiatoxin (**315**) (Figure 57), in addition to the known aplysiatoxin, debromoaplysiatoxin, and anhydrodebromoaplysiatoxin [123]. Both **315** and anhydrodebromoaplysiatoxin display significant activity against the Chikungunya virus (CHIKV) in infected baby hamster kidney cells (BHK21), with EC_50_ values of 2.7 and 22.3 µM, respectively. Debromoaplysiatoxin is the most potent of these five compounds with EC_50_ 1.3 µM. The two brominated metabolites did not result in any significant inhibition at 10 µM.

**Figure 58:**
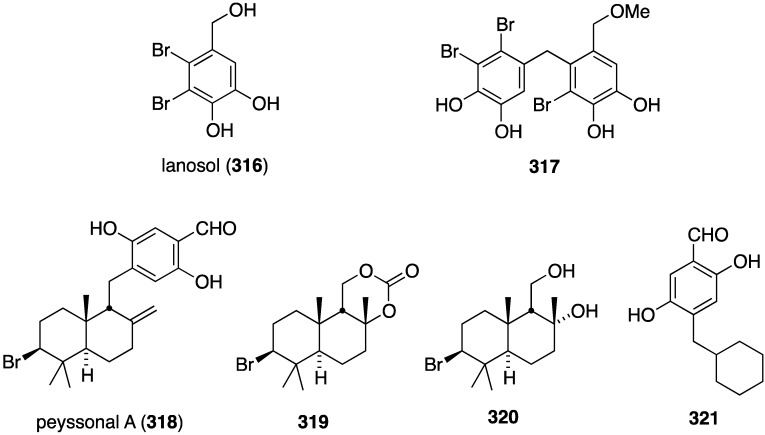
Structures of lanosol (**316**) and **317** from the red alga *Neorhodomela aculeata* [124] and peyssonol A (**318**) and synthetic analogues **319**–**321** [125].

Of several known polybromocatechols isolated from the Korean red alga *Neorhodomela aculeata*, lanosol (**316**) and **317** are active against the human rhinovirus HRV2, IC_50_ 2.50 and 7.11 µg/mL, respectively. The latter polybrominated diphenylmethane is also active towards HRV3, IC_50_ 4.69 µg/mL (Figure 58) [124]. The naturally occurring algae (*Peyssonnelia* sp.) and sponge (*Hyatella intestinalis*) metabolite peyssonol A (**318**) (Figure 58) has been evaluated, along with synthetic stereoisomers, against a recombinant HIV-1 strain (Rep-Rlue Sac II) [125]. Peyssonol A shows the most activity (EC_50_ 1 µM), with analogues **319**–**321** somewhat less active (IC_50_ 2–4 µM).

## 6. Antitumor Compounds

Of enormous concern to all mankind is cancer—the inexorable transformation of normal cells and the proliferation of cancerous cells into tumors. The marine environment provides an array of metabolites active against cancer cells.

**Figure 59:**
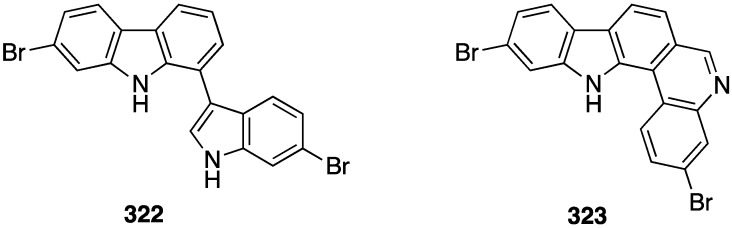
Structures of **322** and **323** from the sponge *Penares* sp. [126].

Amongst all marine life, sponges have afforded the vast majority of anti-tumor compounds. The Vietnamese sponge *Penares* sp. contains the novel alkaloids, **322** and **323** (Figure 59), the former of which is moderately cytotoxic to the human tumor cell lines HL-60 (lung) and HeLa (cervix), IC_50_ 16.1 and 33.2 µM, respectively, whereas **323** is inactive [126].

**Figure 60:**
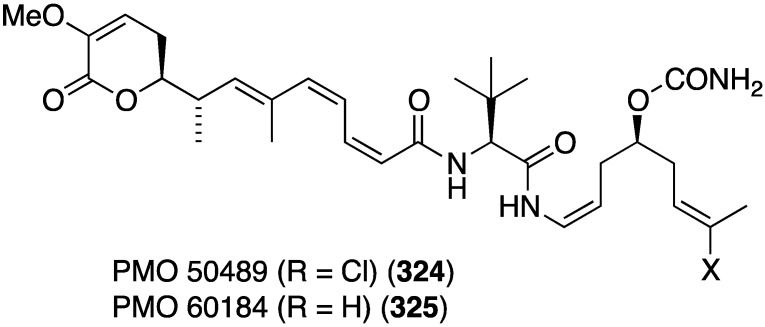
Structures of PM050489 (**324**) and PM060184 (**325**) from the sponge *Lithoplocamia lithistoides* [127].

The novel polyketides, PM050489 (**324**) and PM060184 (**325**), were isolated from the Madagascan sponge *Lithoplocamia lithistoides* (Figure 60) [127]. Both are tubulin-binders, and show excellent growth inhibition against human tumor cells, including HT-29 (colon), A-549 (lung), and MDA-MB-231 (breast), with GI_50_ values of 0.46, 0.38, and 0.45 (**324**) and 0.42, 0.59, and 0.71 (**325**) nM, respectively.

**Figure 61:**
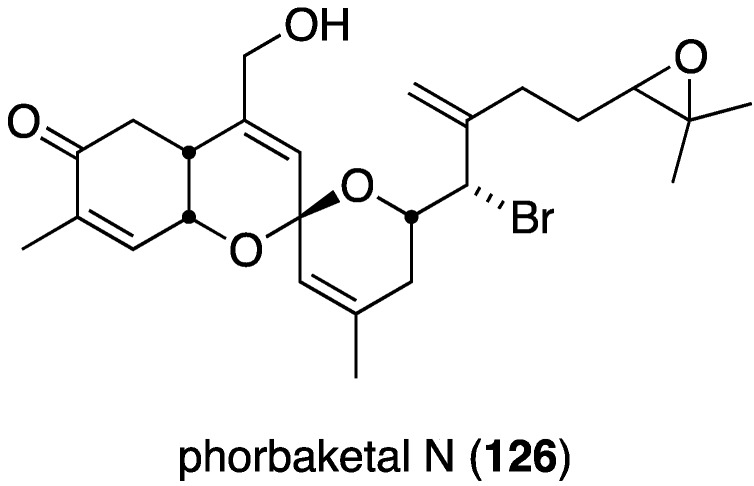
Structure of phorbaketal N (**326**) from the sponge *Phorbas* sp. [128].

The new sesterterpenoid phobaketals N (**326**) (Figure 61) isolated from a Korean *Phorbas* sp. sponge has potent cytotoxicity against the human pancreas cell line (Panc-1) and the human renal cell lines (A498 and ACHN) with IC_50_ 11.4, 18.7, and 24.4 µM, respectively [128]. Of the two nonbrominated phorbaketals (L and M) also isolated from this sponge, only phorbaketal L shows cytotoxicity (A498, 17.3 µM).

**Figure 62:**
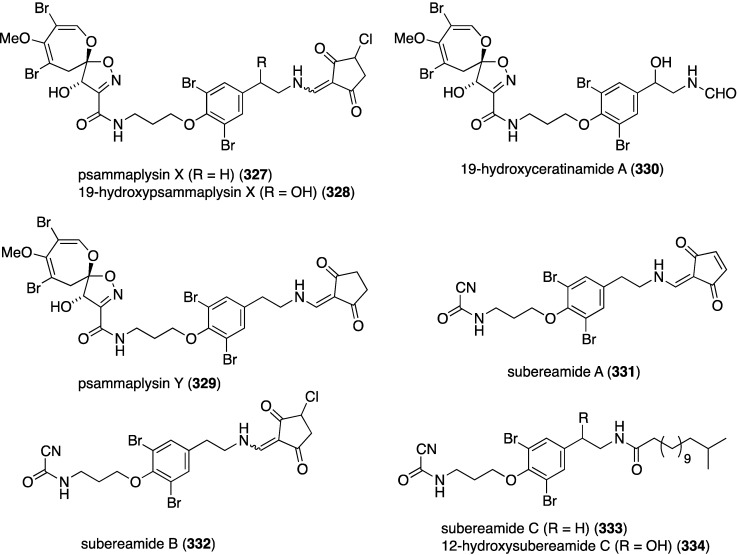
Structures of psammaplysins **327**–**330** and ceratinamines **331**–**334** from the *Suberea* sp. sponge [129].

A Micronesian specimen of a *Suberea* sp. sponge has afforded four new psammaplysins (**327**–**330**) and four new ceratinamines (**331**–**334**) (Figure 62), along with nine previously known bromotyrosine analogues [129]. Whereas the ceratinamines are essentially devoid of cytotoxicity against a panel of human cancer cell lines, the psammaplysins are quite active (Table 10). Included in the table are some of the isolated known analogues and the positive control doxorubicin.

**Figure 63:**
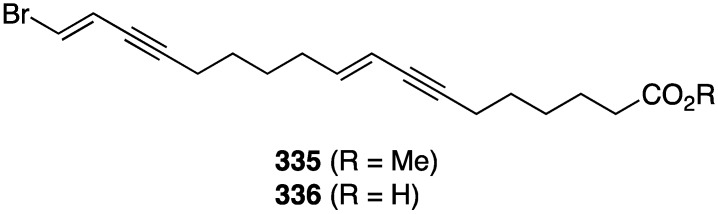
Structures of brominated acetylenes **335** and **336** from the *Haliclona* sp. sponge [130].

Two new brominated acetylenes, **335** and **336**, were isolated from a collection of *Haliclona* sp. sponge living in Saudi Arabia waters (Figure 63) [130]. Both are active towards MCF-7 human breast cancer cells, IC_50_ 32.5 and 50.8 µM, respectively, but not against HepG2 (human hepatocellular carcinoma), WI-38 (skin carcinoma), and Vero (African green monkey kidney).

**Figure 64:**
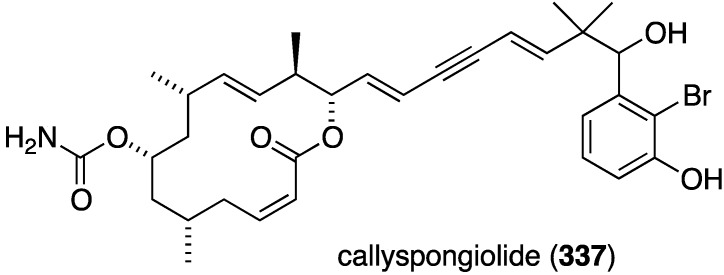
Structure of callyspongiolide (**337**) from the sponge *Callyspongia* sp. [131].

Callyspongiolide (**337**) is a novel macrolide characterized from the Indonesian sponge *Callyspongia* sp. (Figure 64) [131]. This metabolite exhibits potent cytotoxicity against L5178Y mouse lymphoma cells, human Jurkat J16 T and Ramos B lymphocytes with IC_50_ values of 320, 70, and 60 nM, respectively.

**Table 10:** Growth inhibition (GI_50_ µM) of psammaplysins and known analogues against human cancer cell lines [129].

Compound	HCT-15	PC-3	ACHN	MDA-MB-21	NUGC-3	NIC-H23
psammaplysin X (**327**)	3.3	2.3	3.3	1.2	3.5	6.4
10-hydroxypsammaplysin X (**328**)	3.5	2.1	2.5	0.8	4.0	3.5
psammaplysin A	3.9	6.9	5.1	4.3	3.8	12.4
psammaplysin B	4.0	2.7	1.6	0.53	2.5	3.7
psammaplysin D	24	25	27	21	26	27
psammaplysin E	7.4	3.7	10.3	3.9	4.0	7.0
19-hydroxypsammaplysin E	3.8	1.4	2.3	0.51	2.3	3.6
moloka’iamine	>70	>70	>70	>70	>70	>70
7-hydroxymoloka’iamine	>70	>70	>70	>70	>70	>70
ceratinamine	>70	>70	>70	>70	>70	>70
hydroxyceratinamine	>70	>70	>70	>70	>70	>70
doxorubicin	1.4	0.52	2.0	1.8	0.51	1.9

**Figure 65:**
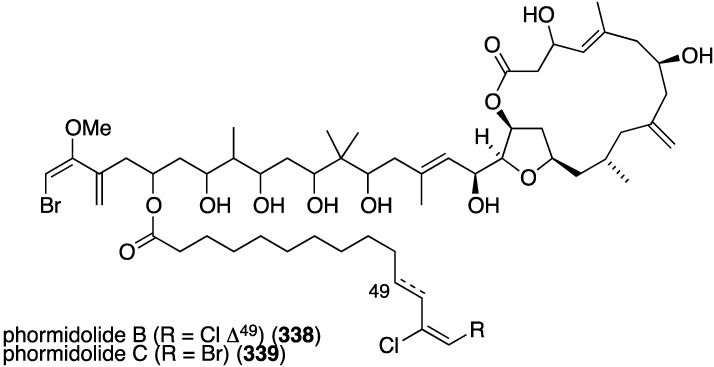
Structures of phormidolides B (**338**) and C (**339**) from a sponge of the Petrosiidae family [132].

From a sponge of the Petrosiidae family were isolated two new macrolides, phormidolides B (**338**) and C (**339**) (Figure 65) [132], which are structurally related to the known phormidolide A and oscillariolide. The new macrolides display growth inhibition of these human cancer cell lines: A-549 (lung), HT-29 (colon), and MDA-MB-231 (breast) with IC_50_ values for **338**/**339** of 1.4/1.3, 1.3/0.8, and 1.0/0.5 µM, respectively.

**Figure 66:**
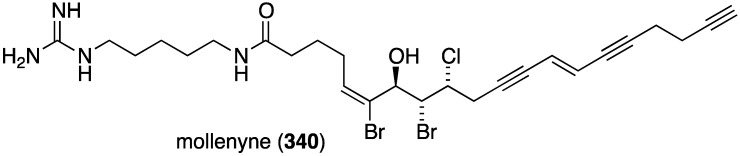
Structure of mollenyne (**340**) from the sponge *Spirastrella mollis* [133].

The Bahamas sponge *Spirastrella mollis* contains mollenyne (**340**) (Figure 66), a highly cytotoxic chlorodibromohydrin towards HCT-116 (human colon cancer cells) with IC_50_ 1.3 µg/mL [133]. The positive control etoposide has IC_50_ 0.55 µg/mL.

**Figure 67:**
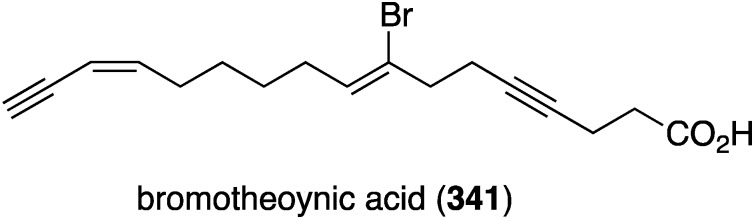
Structure of bromotheoynic acid (**341**) from the sponge *Theonella swinhoei* [134].

A collection of the sponge *Theonella swinhoei* from Japanese waters (Tanegashima, Kagoshima Prefecture) has provided bromotheoynic acid (**341**) (Figure 67) [134]. This new brominated C_17_ acetylenic acid inhibits the cell proliferation of U937 and HL60 (human leukemia), A549 and H1299 (human lung), and HEK293 (human embryonic kidney) with values of IC_50_ 24, 27, 58, 72, and 40 mg/mL, respectively. Bromotheoynic acid also inhibits the maturation of starfish (*Asterina pectinifera*) oocytes at a concentration of 100 ng/mL.

**Figure 68:**
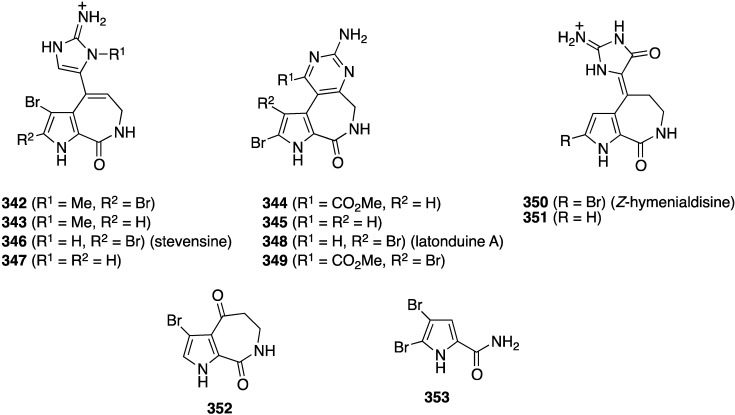
Structures of brominated alkaloids **342**–**353** from the sponge *Stylissa* sp. [135].

The sponge *Stylissa* sp. from the Derawan Islands in Indonesia has yielded four new brominated alkaloids, **342**–**345** (Figure 68), along with eight known analogues, including **346**–**353** [135]. All compounds were screened for their cytotoxicity towards mouse lymphoma cells L5187Y (Table 11), but only **342**, **348**, **350**, and **351** show strong activity in this screen. The presence of an *N*-methyl and a carbonyl group in the imidazole ring increases activity (**342** *vs.* **346**; and **350**/**351**), and the presence or absence of bromine may not always have a positive influence on the activity (**346** *vs.* **347**).

**Table 11:** Cytotoxicity of brominated alkaloids **342**–**353** against mouse lymphoma cells L5187Y [135].

Alkaloid	L5178Y% of Inhibition Concentration (10 µg/mL)	EC_50_
**342**	86.1	3.5
**343**	8.1	–
**344**	10.2	–
**345**	6.6	–
**346**	7.5	–
**347**	15.1	–
**348**	89.3	9.0
**349**	1.7	–
**350**	99.6	1.8
**351**	101.0	2.1
**352**	9.0	–
**353**	33.8	–
Kahalalide F (control)	–	6.3

**Figure 69:**
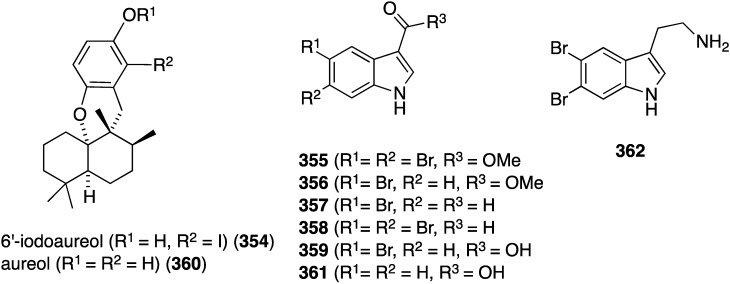
Structures of 6′-iodoaureol (**354**), aureol (**360**), and indoles **355**–**362** from the sponge *Smenospongia* sp. [136].

An examination of the Thai sponge *Smenospongia* sp. gathered in the Andaman Sea has uncovered the novel 6′-iodoaureol (**354**) and the bromoindoles **355**–**359** (Figure 69), isolated from a natural source for the first time, along with several other known natural products [136]. The new compounds, **354**–**359**, and the known **360**–**362** were screened against a battery of human cell lines for cytotoxicity (Table 12). Only 5,6-dibromotryptamine (**362**) shows good activity against MOLT-3 (human leukemia) and HeLa cells, with non-halogenated aureol (**360**) and **355** showing some modest cytotoxicity against HL-60 and HeLa, respectively.

**Table 12:** Cytotoxicity of **354**–**362** against human cancer cells (IC_50_ µM) [136].

Compound	MOLT-3	HepG2	A549	HuCCA-1	HeLa	HL-60	MDA-MB-231
**354**	39.8	44.7	68.2	63.6	61.4	43.2	44.7
**355**	>100	36.1	>100	>100	13.0	>100	>100
**357**	>100	>100	>100	>100	>100	>100	>100
**358**	>100	>100	>100	>100	69.3	–	>100
**359**	>100	>100	>100	>100	69.3	–	>100
**360**	24.8	29.2	76.4	87.6	62.1	14.6	29.7
**361**	73.2	>100	>100	>100	7.81	64.3	>100
**362**	5.4	23.1	78.6	23.6	9.4	–	34.1
Etoposide	0.03	–	–	–	–	1.18	–
Doxorubicin	–	0.69	0.43	0.69	0.38	–	0.62

**Figure 70:**
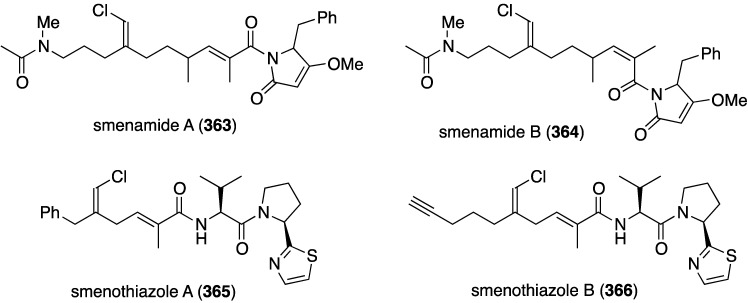
Structures of smenamides A (**363**) and B (**364**), and smenothiazoles A (**365**) and B (**366**) from the sponge *Smenospongia aurea* [137,138].

Two studies of the chemical content of the Caribbean sponge *Smenospongia aurea*, collected in the Bahamas along the coast of Little Inagua, has led to the chlorinated smenamides A (**363**) and B (**364**), and smenothiazoles A (**365**) and B (**366**) (Figure 70) [137,138]. Whereas the smenamides exhibit selectivity and nanomolar cytotoxic activity towards Calu-1 (lung) cancer cells, the smenothizoles are equally active and selective against A2780 (ovarian) cancer cells.

**Figure 71:**
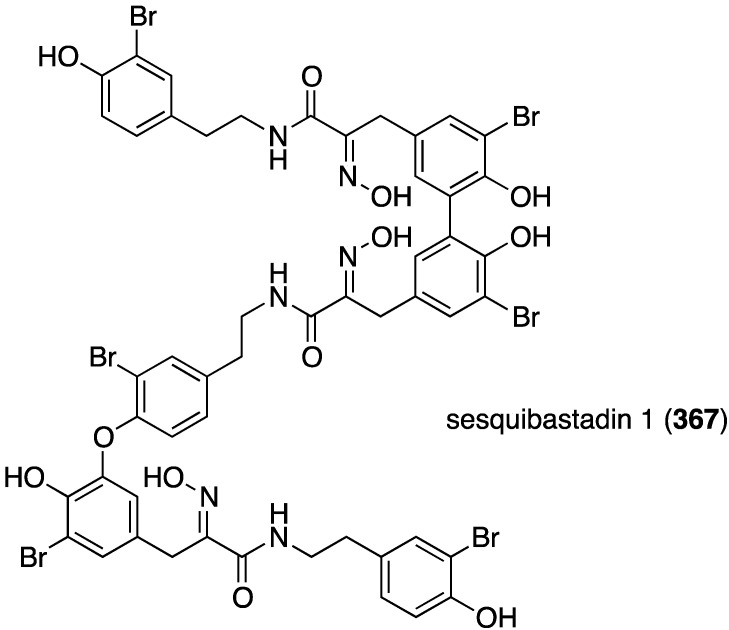
Structure of sesquibastadin 1 (**367**) from the sponge *Ianthella basta* [139].

The first naturally occurring trimeric hemibastadin, sesquibastadin 1 (**367**), was characterized from the sponge *Ianthella basta*, found in Ambon, Indonesia (Figure 71) [139]. The known bastadins 3, 6, 7, 11, and 16 were also isolated. Whereas sesquibastadin 1 does not display cytotoxicity against L5178Y cells (mouse lymphoma), bastadins 6, 7, 11, and 16 do inhibit cell proliferation, with IC_50_ values of 1.5, 5.3, 3.7, and 1.9 µM, respectively. However, sesquibastadin 1 is a potent protein kinase inhibitor as seen in a later section.

**Figure 72:**
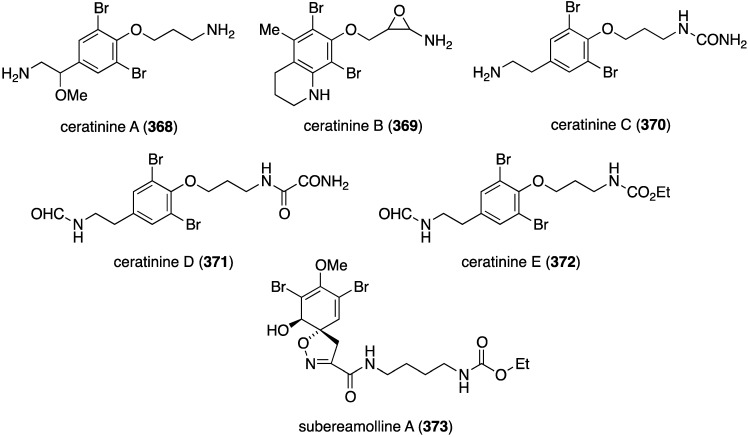
Structures of ceratinines A–E (**368**–**372**) and subereamolline A (**373**) from the sponge *Pseudoceratina arabica* [140].

The Red Sea sponge *Pseudoceratina arabica* from Hurghada at the Egyptian coast contains the new ceratinines A–E (**368**–**372**) (Figure 72), in addition to several known brominated alkaloids [140]. Screening of all isolated compounds against the highly metastatic MDA-MB-251 human breast cancer cell line reveals that only the known subereamolline A (**373**) is highly active, showing IC_50_ 1.7 µM.

**Figure 73:**
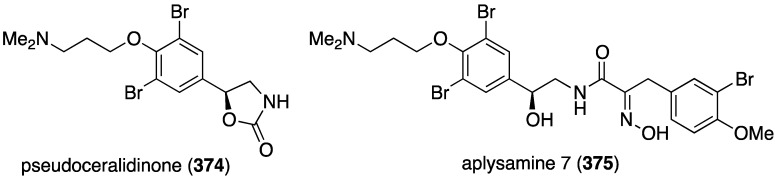
Structures of pseudoceralidinone A (**374**) and aplysamine 7 (**375**) from *Pseudoceratina verrucosa* [141].

An Australian version of *Pseudoceratina verrucosa* has furnished the new pseudoceralidinone A (**374**) and aplysamine 7 (**375**) (Figure 73), in addition to the known aerophobin 2, fiscularin 2, and fistularin 3 (not shown) [141]. Of these five bromotyrosines, only aplysamine 7 (**375**) shows cytotoxicity towards PC3 (prostate) cancer cells with IC_50_ 4.9 µM. All five compounds are inactive against HeLa (cervical) and NFF (human neonatal foreskin fibroblast) cells (IC_50_ > 10 µM).

**Figure 74:**
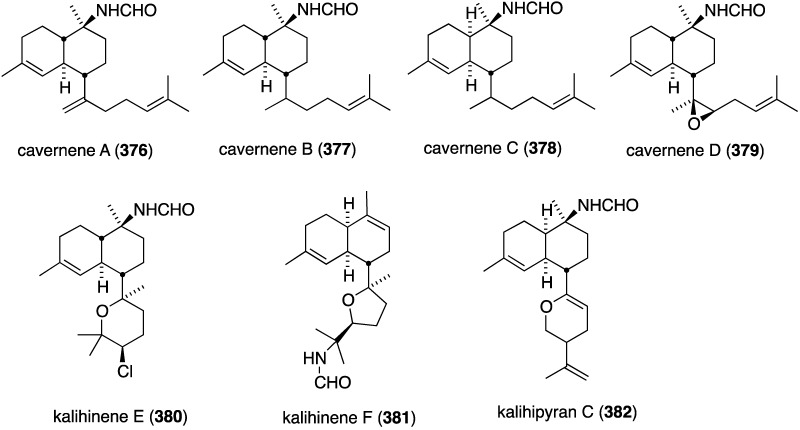
Structures of cavernenes A–D (**376**–**379**), kalihinenes E (**380**) and F (**381**) and kalipyran C (**382**) from the sponge *Acantella cavernosa* [142].

The South China Sea sponge *Acanthella cavernosa* has afforded the new cavernenes A–D (**376**–**379**), kalihinenes E (**380**) and F (**381**), and kalihipyran C (**382**) (Figure 74), in addition to several known analogues [142]. These metabolites were screened against several human cancer cell lines (Table 13). Cavernenes A and B display modest cytotoxicity towards HCT-116, and cavernene D shows slight activity against all five cell lines. The other new compounds (**378**, **379**, **381**, **382**) are inactive across the board.

**Table 13:** Cytotoxicity of **376**, **377**, **380**, and selected known analogues against human cell lines (IC_50_ µM) [142].

Compound	HCT-116	A549	HeLa	QGY-7701	MDA-MB-231
**376**	6.31	>50	>50	>50	>50
**377**	8.99	>50	>50	>50	>50
**380**	14.36	>50	13.36	17.78	12.84
kalihipyran A	>50	13.09	11.19	13.53	>50
15-formamido-kalihinene	>50	17.53	14.74	16.39	>50
10-formamido-kalihinene	>50	6.98	13.30	14.53	6.84
kalihinene X	12.25	8.55	10.59	13.02	7.46
kalihinene Y	>50	17.12	10.05	14.41	15.23
camptothecin	9.25	2.32	6.98	4.05	0.50

A number of known marine organohalogens were examined for possible cytotoxicity against cancer cell lines during the period covered by this review. To conserve space, their structures are not shown. A review of the antitumor activity of the *Jaspis* sponges is available [143]. The *Fascaplysinopsis* sp. sponge metabolite fascaplysin displays excellent cytotoxicity against chemoresistant SCLC (small cell lung cancer) cell lines, by multiple mechanisms [144]. Other cell lines are also discussed. The *Suberea* sp. sponge alkaloids ma’edamines A and B display significant cytotoxicity against COLO 205 (human colon cancer), MCF-7 (human breast cancer), and A549 (human lung) with IC_50_ values of 7.9/10.3, 6.9/10.5, and 12.2/15.4 for ma’edamines A/B, respectively [145]. Synthetic analogues show activity against three breast cancer cell lines representing hormone receptor positive and HER2 positive breast cancer [146]. The bis-indole alkaloid 6″-debromohamacanthin A from a *Spongosorites* sp. sponge inhibits angiogenesis in human umbilical vascular endothelial cells and mouse embryonic cells [147]. The *Pseudoceratina* sp. alkaloids ceratamines A and B disrupt microtubule dynamics, which provides an explanation for their pronounced antimitotic activity (lower micromolar) [148]. The well known dibromo-dihydroxyoxocyclohexenyl acetonitrile has excellent activity against the K562 leukemia cell line (IC_50_ 1.4 µg/mL) [149]. The known spirastrellolides A and B were isolated from the sponge *Epipolasis* sp. for the first time as free acids, and not as methyl esters. Both macrolides are cytotoxic to HeLa cells, with IC_50_ 20 and 40 nM, respectively [150].

The previously cited dictyodendrins F–I (**74**–**77**) (Figure 14) are cytotoxic towards the SW620 (human colon) cancer cell line with IC_50_ values of 8.5, 2.0, 16, and 10 µM, respectively. Dictyodendrin J is not cytotoxic. None of the five compounds is cytotoxic towards the multi-drug resistant variant SW620 Ad300 [49]. The kalihinols M–T (**100**–**107**) (Figure 18) were screened against several human cancer cell lines, along with some previously known kalihinols, and show weak to modest cytotoxicity (Table 14) [55].

The aforementioned new hamigerans F–J (**204**–**208**) (Figure 36) all show some degree of cytotoxicity towards HL-60 (human promyelocytic leukemia) with F (**204**), G (**205**), and **209** showing IC_50_ values of 4.9, 2.5, and 5.6 µM, respectively. The known hamigeran B is 3.4 µM [83]. The two most active hamigerans, G and B, share the same electrophilic 1,2-dione functionality. Of the three psammaplysins **252**–**254** (Figure 45), psammaplysin F (**254**) is moderately cytotoxic against the HepG2 human carcinoma cell line (IC_50_ 3.7 µM). Psammaplysins G (**253**) and H (**252**) show IC_50_ values of 17.4 and >40 µM, respectively [104].

**Table 14:** Cytotoxicity of kalihinols M–T (100–107) and related kalihinols against human cancer cell lines (IC_50_ µM) [55].

Kalihinol	HCT-116	H1299	CT-26
kalihinol O (**102**)	5.97	–	–
kalihinol P (**103**)	10.68	26.21	–
kalihinol Q (**104**)	20.55	–	–
kalihinol R (**105**)	13.44	–	–
kalihinol E	18.31	–	–
kalihinol A	17.40	–	–
10-*epi*-kalihinol X	8.21	–	–
10-*epi*-kalihinol I	28.67	–	–
10-β-formamidokalihinol-A	–	–	28.82

**Figure 75:**
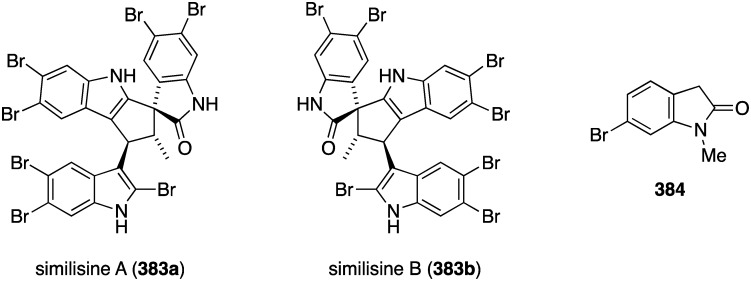
Structures of similisines A (**383a**) and B (**383b**), and oxindole **384** from *Laurencia similis* [151].

Red marine algae are also an excellent source of novel antitumor compounds with genus *Laurencia* in the limelight. A collection of *Laurencia similis* from the South China Sea has yielded the novel enantiomeric spiro-trisindoles similisines A (**383**) and B (**383b**), along with the new oxindole **384** (Figure 75) [151]. The racemate **383** was separated into similisines A and B by enantioselective HPLC. All three compounds were screened against eight human cancer cell lines but only oxindole **384** shows (weak) activity against HL-60 (leukemia) and JURKA (leukemia) with values of IC_50_ 35.06 and 53.27 µM, respectively.

**Figure 76:**
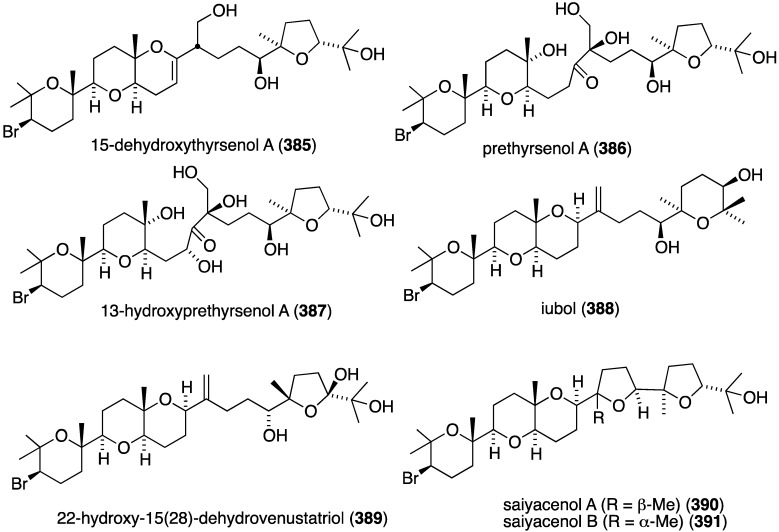
Structures of polycyclic triterpenoids **385**–**391** from *Laurencia viridis* [152,153,154].

An extensive examination of *Laurencia viridis* from the Canary Islands led to seven new brominated polyether triterpenoids, 15-dehydroxythyrsenol A (**385**), prethyrsenol A (**386**), 13-hydroxyprethyrsenol A (**387**) [152], iubol (**388**), 22-hydroxy-15(28)-dehydrovenustatriol (**389**) [153], and saiyacenols A (**390**) and B (**391**) [154] (Figure 76), along with two new non-brominated analogues 1,2-dehydropseudodehydrothyrsiferol and secodehydrothyrsiferol (not shown) [153]. These new oxasqualenoids were screened against several human cancer cell lines (Table 15). Jurkat cells are clearly the most sensitive to these brominated polyethers. The non-brominated secodehydrothyrsiferol shows IC_50_ 2.5 µM in this assay.

**Table 15:** Cytotoxicity of polycyclic triterpenoids **385**–**391** against human cancer cell lines (IC_50_ µM) [152,153,154].

Compound	Jurkat ^a^	MM144 ^b^	HeLa ^c^	CAD-ES-1
**385**	7.6	7.3	23.0	16.5
**386**	8.2	10.2	29.0	14.5
**387**	7.2	15.5	26.0	3.1
**388**	3.5	13.0	27.0	11.0
**389**	2.0	–	2.9	–
**390**	7.8	27.0	27.5	25.5
**391**	2.7	11.0	24.5	14.0

^a^ T-cell acute leukemia; ^b^ multiple myeloma; ^c^ cervical carcinoma; ^d^ Ewing’s sarcoma.

**Figure 77:**
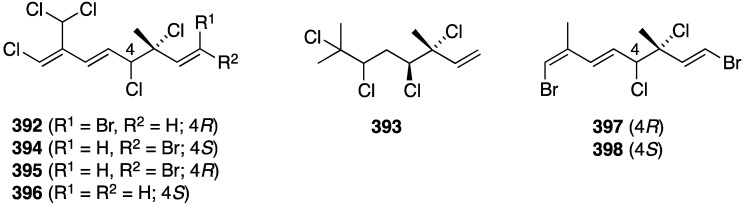
Structures of halogenated monoterpenes from *Plocamium suhrii* [156].

The polybromoindoles from *Laurencia brongniarii* (Figure 21) were tested for cytotoxicity, but in this group only 2,4,5,6-tetrabromo-3-methylthioindole shows activity against Hep3B (liver carcinoma) and MCF-7 (breast carcinoma); IC_50_ 7.7 and 10.5 µM, respectively. For comparison, the non-halogenated doxorubicin has values of IC_50_ 1.2 and 1.5 µM, respectively. Other cell lines examined were HepG2, MDA-MB-231, and A549 [62]. Of the six new laurane-type sesquiterpenes from *Laurencia okamurai* (Figure 40), only 3β-hydroperoxyaplysin (**231**) and 3β-hydroxyaplysin show any cytotoxicity towards the A-549 cell line (IC_50_ 35.3 and 15.4 µM, respectively. All other compounds are IC_50_ > 100 µM [89]. The known bis-(2,3-dibromo-4,5-dihydroxyphenyl)methane from *Laurencia nana* and *Rhodomela confervoides* displays significant growth inhibition against some cell lines (IC_50_ µg/mL): HeLa (17.6), RKO (colon; 11.4), HCT-116 (colon; 10.6), BEL-7402 (hepatoma; 8.7), U87 (glioblastoma; 23.7), and HUVEC (vascular endothelial; 30.2). Moreover, this compound induces detachment of the cancer cells and apoptosis, and inhibits metastasis [155]. Although the *Asparagopsis taxiformis* cyclopentenones mahorone (**108**) and 5-bromomahorone (**109**) (Figure 19) are not cytotoxic towards several human cancer cell lines (A549, HepG2, HT29, and MCF7), mahorone is cytotoxic against healthy liver cells (54% growth inhibition at 5 µM) [58]. The bromoditerpene from *Sphaerococcus coronopifolius*, sphaerodactylomelol (**122**) (Figure 21), shows some cytotoxicity and anti-proliferative property against HepG-2 cells (IC_50_ 720 and 280 µM, respectively). The known sphaerococcenol shows IC_50_ 43 µM for anti-proliferative activity. For comparison, cisplatin and tamoxifen have IC_50_ values of 75 and 46 µM, respectively [63]. In contrast, bromophycoic acid E (**131**) from *Callophycus* sp. (Figure 22) shows cytotoxicity of IC_50_ 6.8 µM as the mean value of 14 human cancer cell lines. The other bromophycoic acids are less active [64]. The South African *Plocamium suhrii* has provided the new halogenated monoterpenes **392** and **393** (Figure 77) and the known **394**–**398** [156]. These compounds were screened against the human esophageal cancer cell line WHCO1 with the following IC_50_ values (µM): **392** (9.3), **393** (7.9), **394** (6.6), **395** (9.9), **396** (8.5), **397** (8.4), and **398** (15.1). For comparison, cisplatin has IC_50_ 13 µM. Tetrachloro monoterpene **393** was previously isolated from *Plocamium corallorhiza* but not fully characterized [156].

**Figure 78:**
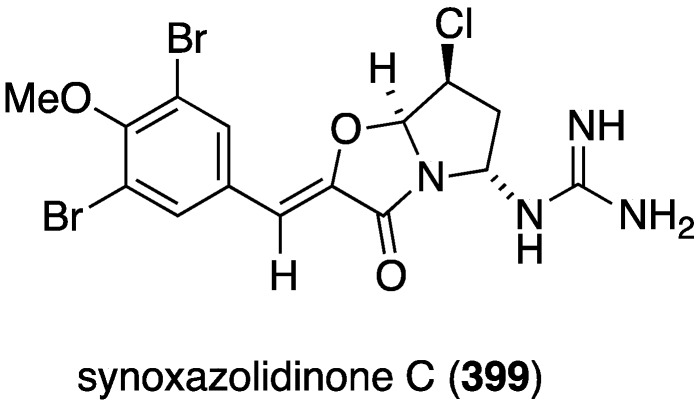
Structure of synoxazolidinone (**399**) from the ascidian *Synoicum pulmonaria* [157].

The *Synoicum* sp. eudistomins Y_2_–Y_7_ (**132**–**137**) (Figure 23) were screened against A549 cancer cells, but only the previously known eudistomin Y_9_ shows cytotoxicity (IC_50_ 17.9 µM) (doxorubicin has LC_50_ 3.3 µM) [65]. Another known *Synoicum* sp. ascidian metabolite, prunolide A, is cytotoxic to breast cancer cell lines at <1 µM [122]. The newest member of the synoxazolidinone family of metabolites from *Synoicum pulmonaria* is synoxazolidinone C (**399**) (Figure 78), which is cytotoxic to several human cancer cell lines: A2058 (melanoma), MCF-7 (breast), and HT-29 (colon) at IC_50_ 30.5 µM. This compound also kills normal lung fibroblast cells (MRC-5) at the same concentration [157].

**Figure 79:**
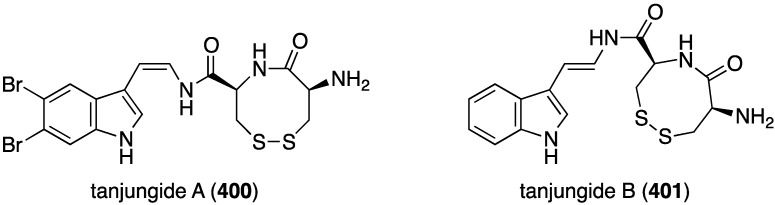
Structures of tanjungides A (**400**) and B (**401**) from the tunicate *Diazona* cf *formosa* [158].

The tunicate *Diazona* cf *formosa* living off the coast of Timor Island, near Indonesia, has afforded the novel tanjungides A (**400**) and non-halogenated B (**401**) (Figure 79) [158]. Cytotoxicity of these bromoindoles was assayed against A549, HT29, and MDA-MB-231 human cancer cell lines. The data show that tanjungide A (**400**) is strongly active against the three cell lines: IC_50_ 0.33, 0.19, and 0.23 µM, respectively. Tanjungide B is much less active (IC_50_ 2.50, 2.31, and 1.63 µM, respectively).

**Figure 80:**
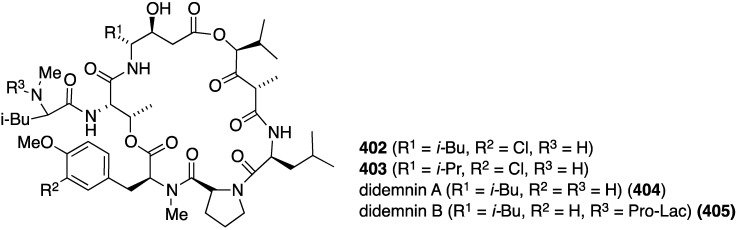
Structures of didemnins **402**–**405** from the tunicate *Trididemnum solidum* [159].

The two new chlorinated didemnins **402** and **403** were isolated from the tunicate *Trididemnum solidum* from Little Cayman island along with the known nonchlorinated didemnins A (**404**) and B (**405**) (Figure 80) [159]. All four didemnins were evaluated for cytotoxicity against human cancer cells (Table 16), and all strongly inhibit cell proliferation in the cancer cell lines, especially didemnuns A and B, but not in the noncancerous VERO cell line.

**Table 16:** Anti-cell proliferative activity of didemins **402**–**405** (IC_50_ µM) [159].

Didemnin	SK-MEL ^a^	KB ^b^	BT-549 ^c^	SK-OV-3 ^d^	VERO ^e^
**402**	0.12	0.26	0.16	0.26	4.8
**403**	0.06	0.42	0.16	0.38	2.08
**404**	0.055	0.16	0.07	0.16	4.78
**405**	0.022	0.09	0.02	0.1	0.15
Doxorubicin	1.1	1.66	1.01	1.66	14

^a^ Melanoma; ^b^ epidermal carcinoma; ^c^ breast; ^d^ ovarian; ^e^ monkey kidney fibroblasts.

**Figure 81:**
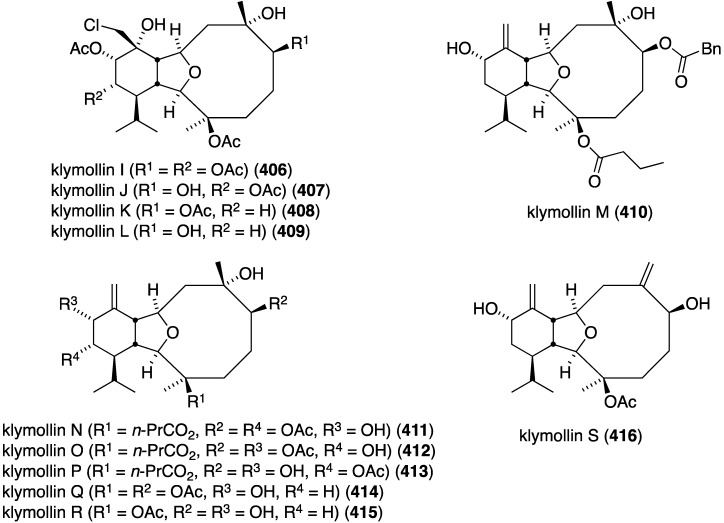
Structures of klymollins I–S (**406**–**416**) from the soft coral *Klyxum molle* [160].

The Formosan soft coral *Klyxum molle* has afforded 11 new eunicellin-type diterpenoids, klymollins I–S (**406**–**416**) four of which, I–L, contain chlorine (Figure 81) [160]. Of the klymollins screened for cytotoxicity against the human cancer cell lines K562 myeloblastoid (leukemia), Molt-4 (lymphoblastic leukemia), and T47D (breast carcinoma) only klymollin M (**410**) shows activity: ED_50_ 7.97, 4.35, and 8.58 µM, respectively.

**Figure 82:**
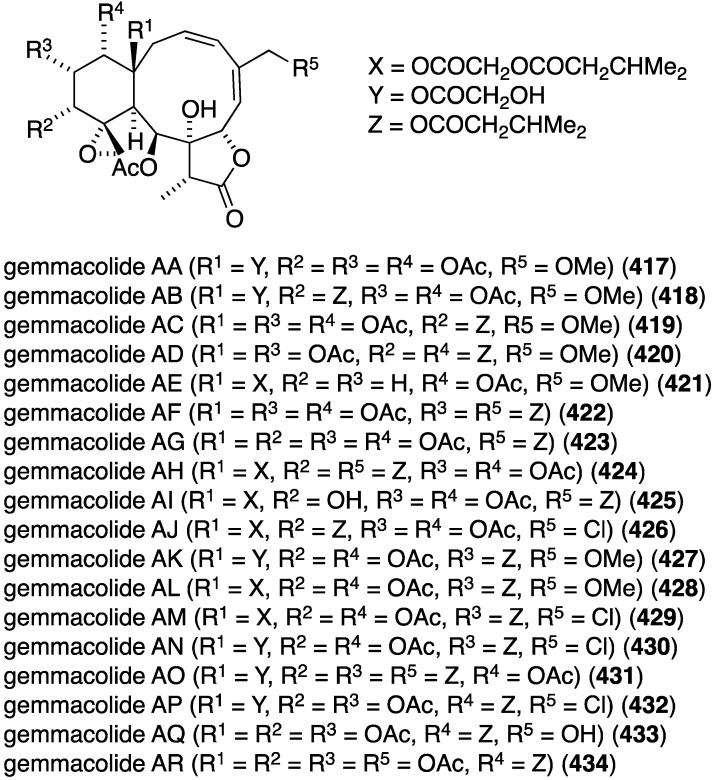
Structures of gemmacolides AA–AR (**417**–**434**) from the gorgonian *Dichotella gemmacea* [161].

The earlier discussed gemmacolides and dichotellides from the gorgonian *Dichotella gemmacea* (Figure 1 and Figure 2) display some antitumor properties [30,31,32,33,34,161]. Against the human cancer cell lines A549 (lung adenocarcinoma) and MG63 (osteosarcoma), gemmacolides V (**9**) and Y (**11**) show IC_50_ values of <1.5 and <0.3 µM, respectively, against A549; and gemmacolide Y has IC_50_ < 0.3 µM towards MG63. The positive control adriamycin gives IC_50_ 2.8 and 3.2 µM for these two cell lines, respectively [31]. Juncin R shows 5.6 µM towards MG63 cells [30]. Of gemmacolides G–M, only gemmacolide J shows good growth inhibition against A549 cells (IC_50_ < 1.4 µM) [33]. The dichotellides F–U are not cytotoxic to the human cancer cell lines SW1990, MCF-7, HepG2, and H460 cell lines, but dichotellide C displays (marginal) activity towards SW1990 (pancreatic) with IC_50_ 45 µM (fluorouracil, IC_50_ 121 µM) [34]. A later tour de force examination of *Dichotella gemmacea* revealed the presence of 18 new gemmacolides AA–AR (**417**–**434**) (Figure 82) [161]. The most cytotoxic compound in the A549 and MG63 cell line assays is gemmacolide AH (**424**) with IC_50_ for both cell types (adriamycin: IC_50_ 2.8 and 3.2 µM).

**Figure 83:**
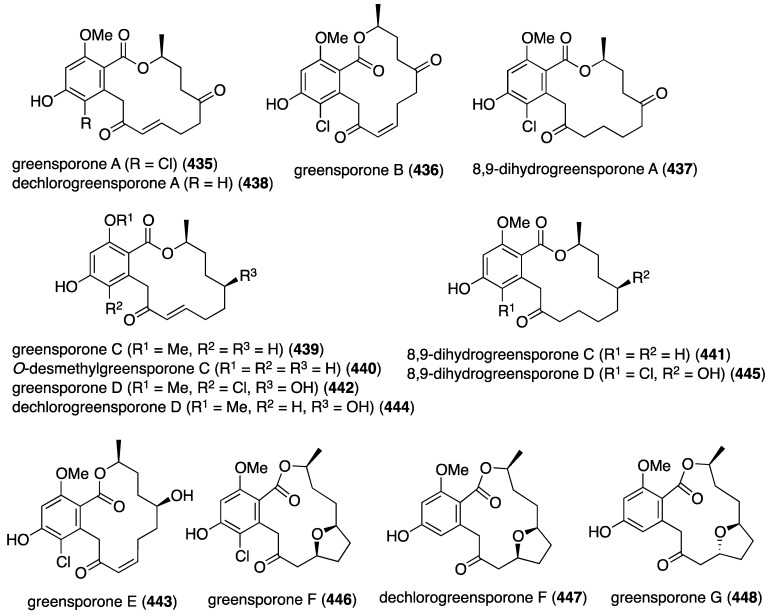
Structures of greensporones **435**–**448** from the freshwater aquatic fungus *Halenospora* sp. [162].

A study of the cochliomycins A–C (**39**–**41**) (Figure 5) reveals no cytotoxicity against A549 and HepG2 cancer cells, but the related LL-Z1640-1 shows modest activity; IC_50_ 44.5 and 98.6 µM, respectively [37]. The structurally related resorcylic acid lactones, greensporones **435**–**448** (Figure 83) from the aquatic fungus *Halenospora* sp., were assayed for antitumor activity [162]. However, only greensporone C (**439**) shows significant cytotoxicity against the cell lines MDA-MB-435 (melanoma) and HT-29 (colon) with IC_50_ 2.9 and 7.5 µM, respectively.

**Figure 84:**
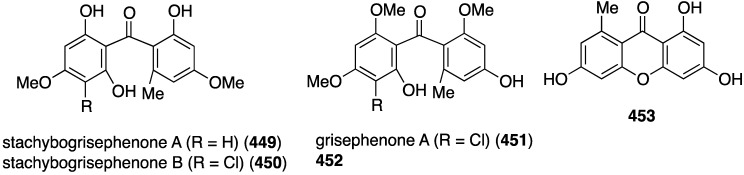
Structures of benzophenones **449**–**452** and xanthone **453** from *Stachybotry* sp. HH1 ZDDS1F1-2 [163].

The sponge-derived fungus *Stachybotry* sp. HH1 ZDDS1F1-2 has yielded several sesquiterpenoids and xanthones, totaling 15 compounds. In addition to the two new xanthones, stachybogrisephenones A (**449**) and B (**450**), the three known compounds grisephenone A (**451**), **452**, and **453** are cytotoxic towards U937, HeLa, and K562 cell lines (Figure 84) [163]. Grisephenone A (**451**) has IC_50_ 22.5 and 14.6 µM towards U937 and HeLa cells, respectively. Compound **452** has IC_50_ 22.3 and 14.0 µM against K562 and HeLa, respectively, and **453** shows IC_50_ 7.2 µM against the HeLa cell line.

**Figure 85:**
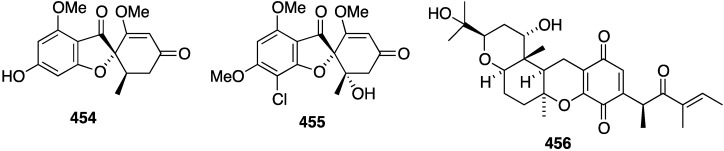
Structures of **454**–**456** from the fungus *Nigrospora* sp. MA75 [164].

In addition to the new griseofulvins **454** and **455**, the mangrove-derived (*Pongamia pinnata*) fungus *Nigrospora* sp. MA75 has afforded the quinone **456**, along with several known compounds (griseofulvins, xanthones, benzophenones) (Figure 85) [164]. Non-halogenated compound **456** is cytotoxic to these human cancer cell lines: MCF-7 (breast), SW1990 (pancreas), HepG2 (hepatocellular liver), NCI-H460 (lung), DU145 (prostate), and SMMC7721 (hepatocellular liver) with these respective IC_50_ values (µg/mL): 4, 5, 20, 11, 17, and 7 µg/mL. For comparison, fluorouracil shows IC_50_ 4, 16, 14, 1, 0.4, and 2 µg/mL, respectively.

**Figure 86:**
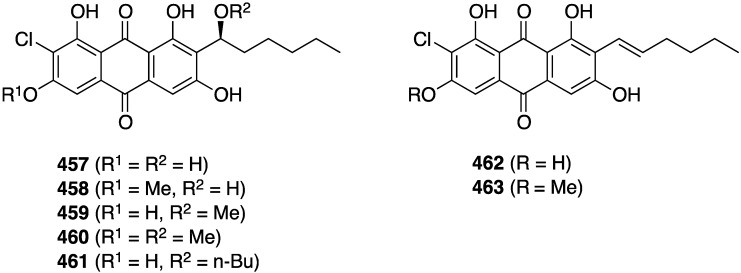
Structures of chlorinated averantin anthraquinones **457**–**463** from the fungus *Aspergillus* sp. SCS10 FO63 [165].

The marine-derived *Aspergillus* sp. SCS10 FO63 fungus produces seven new averantin-type chlorinated anthraquinones **457**–**463** (Figure 86) along with five known analogues [165]. From this group, only 6-*O*-methyl-7-chloroaverantin (**458**) exhibits good cytotoxicity against SF-268 (glioblastoma), MCF-7 (breast), and NCI-H460 (lung) with IC_50_ values of 7.11, 6.64, and 7.42 µM, respectively. For comparison, cisplatin has IC_50_ values of 4.59, 10.23, and 1.56 µM, respectively.

**Figure 87:**
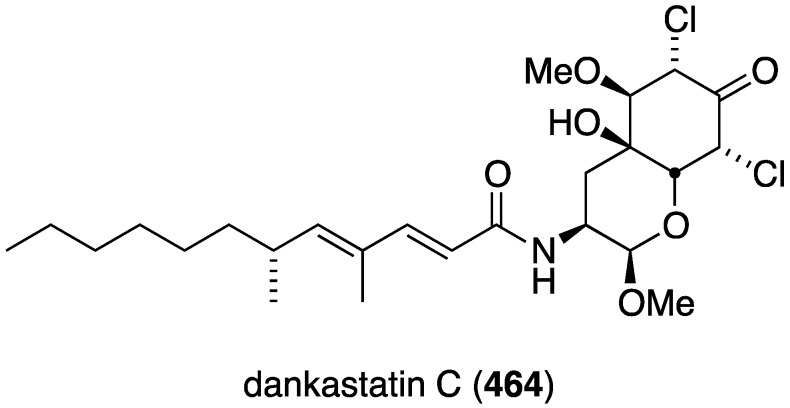
Structure of dankastatin C (**464**) from the fungus *Gymnascella dankaliensis* [166].

The *Homaxinella* sponge-derived fungus, *Gymnascella dankaliensis*, has furnished the new polyketide dankastatin C (**464**) (Figure 87) [166]. This compound displays pronounced cell growth inhibition of the murine P388 leukemia cell line with EC_50_ 57 ng/mL (comparable to 5-fluorouracil with EC_50_ 78 ng/mL).

**Figure 88:**
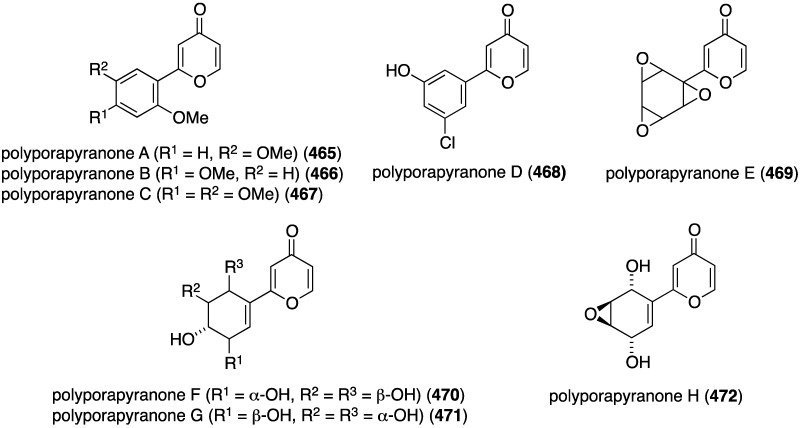
Structures of polyporapyranones A–H (**465**–**472**) from the fungi Polyporales PSU-ES44 and PSU-ES83 [167].

The seagrass (*Thalassia hemprichii*)-derived fungi Polyporales PSU-ES44 and PSU-ES83 have yielded the new polyporapyranones A–H (**465**–**472**) (Figure 88), along with eight known analogues [167]. Of these compounds, only **465** shows moderate activity against Vero cells (IC_50_ 6.93 µg/mL), and no polyporapyranone is active against MCF-7 cells. For comparison, ellipticine has IC_50_ 1.28 µg/mL against these African green monkey kidney fibroblast (Vero) cells.

**Figure 89:**
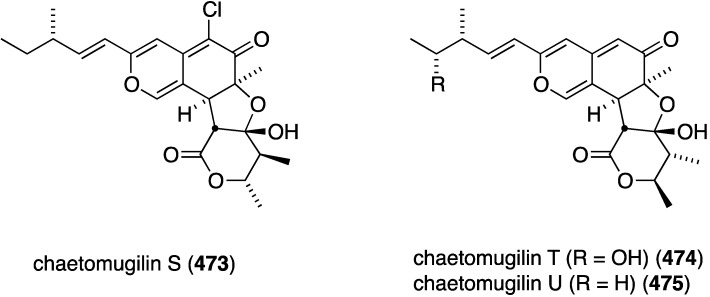
Structures of chaetomugilins S, T, and U (**473**–**475**) from the fungus *Chaetomium globosum* [168].

A strain of the fungus *Chaetomium globosum*, which was obtained from the marine fish *Mugil cephalus*, has produced three new azaphilones, chaetomugilin S (**473**), dechloro-chaetomugilin A (chaetomugilin T) (**474**), and dechloro-chaetomugilin D (chaetomugilin U) (**475**) (Figure 89) [168]. Chaetomugilin S (**473**) is modestly active towards these cell lines: P388, HL-60, L1210, and KB (IC_50_ 46.0, 39.1, 43.7, and 34.5, respectively).

**Figure 90:**
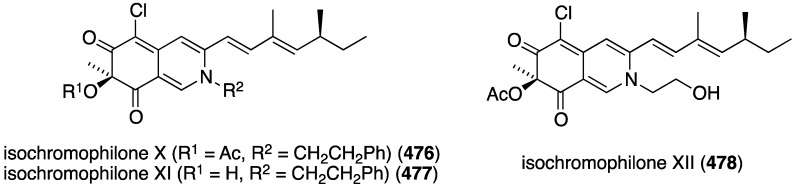
Structures of isochromophilones X–XII (**476**–**478**) from the fungus *Diaporthe* sp. [169].

Three new azaphilones, isochromophilones X–XII (**476**–**478**), have also been found in the fungus *Diaporthe* sp., which was isolated from the mangrove plant *Rhizophora stylosa* of Hainan Province, China (Figure 90) [169]. The familiar sclerotioramine and isochromophilone VI were also isolated. This is the first example of azaphilones being found in *Diaporthe*. Isochromophilone X (**476**) displays moderate cytotoxicity against MCF-7 (breast), SGC-7901 (gastric), SW1116 (colorectal), A549 (lung), and A375 (melanoma) with IC_50_ values of 14.90, 16.84, 24.15, 26.93, and 35.75 µM. The other azaphilones have >50 µM against these cell lines.

**Figure 91:**
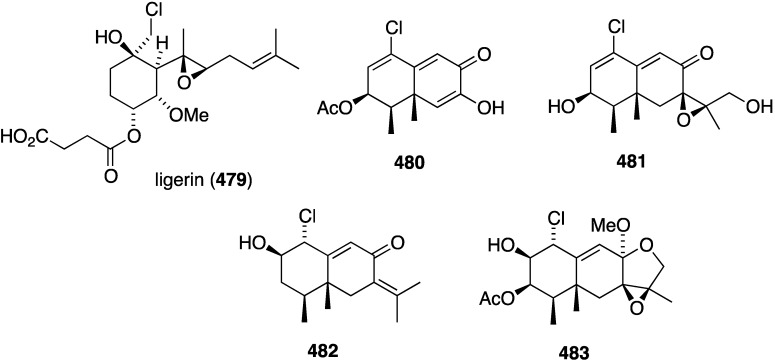
Structures of ligerin (**479**) and **480**–**483** from the fungi *Penicillium* spp. [170,171].

A marine-derived *Penicillium* sp., which was isolated from seawater on the French coast, has yielded an analogue of fumagillin, ligerin (**479**) (Figure 91) [170]. Evaluation of ligerin against these cancer cell lines: KB (nasopharyngeal), AT6-1 (murine prostatic), POS1 and OSRGa (murine osteosarcoma), and L929 (murine fibroblasts) shows antiproliferative activity against all of these cell lines except KB cells. The highest activity of ligerin is seen in the POS1 cell line (IC_50_ 117 nM), which is 20 times more active than the other cell lines. An Antarctic deep-sea fungus, *Penicillium* sp. PR19N-1, has yielded the four novel chlorine-containing sesquiterpenes **480**–**483** (Figure 91) [171]. The known non-chlorinated eremofortine C is also present.

**Figure 92:**
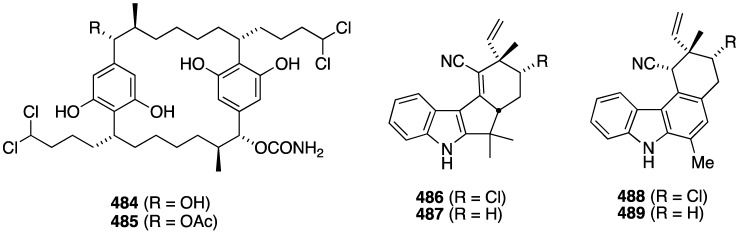
Structures of carbamidocyclophanes F (**484**) and G (**485**) from the cyanobacterium *Nostoc* sp., and fischerindoles **486**–**489** from the cyanobacterium *Fischerella* sp. [172,173].

Cyanobacteria continue to be a major supplier of novel natural products, including halogenated metabolites. The freshwater cyanobacterium *Nostoc* sp. (UIC 10274) from Illinois has afforded the two new carbamidocyclophanes F (**484**) and G (**485**) (Figure 92) [172], both of which are antiproliferative against the human cancer cell lines MDA-MB-435 (breast) and HT-29 (colon) with IC_50_ 0.5–0.7 µM for both **484** and **485**. The cyanobacterium *Fischerella* sp. (SAG 46.79), a rich source of chlorinated indoles, contains the four new fischerindoles **486**–**489** (Figure 92) [173]. Of these four compounds only **487** (deschloro 12-*epi*-fischerindole I nitrile) shows (weak) cytotoxicity towards HT-29 cells (ED_50_ 23 µM). Compounds **488**/**489** are the first carbazole-type fischerindoles to be discovered.

**Figure 93:**
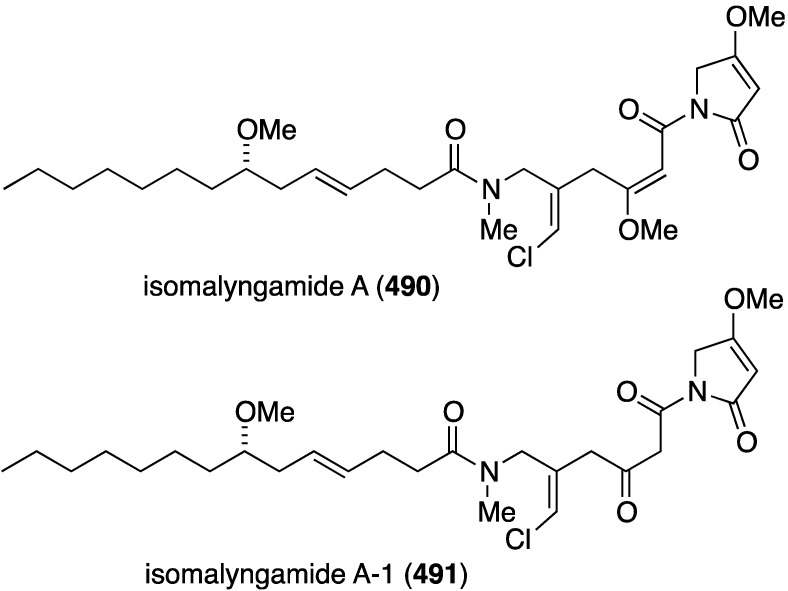
Structures of isomalyngamides A (**490**) and A-1 (**491**) from the cyanobacterium *Lyngbya majuscule* [174].

*Lyngbya* genus is a prolific producer of organohalogens and the Taiwanese *Lyngbya majuscule* has afforded the known isomalyngamide A (**490**) and the new isomeric A-1 (**491**) (Figure 93) [174]. Both compounds are antiproliferative towards MCF-7 and MDA-MG-231 cells (IC_50_ 4.6 and 2.8 µM, respectively, for **490**), and they inhibit the migration of MDA-MB-231 cells (IC_50_ 0.060 and 0.337 µM, for **490** and **491**, respectively). Consistent with an antimetastatic mechanism for these isomalyngamides is that they both inhibit α-2,3-sialyltransferase (IC_50_ 77.2 and 65.7 µM for **490** and **491**, respectively).

**Figure 94:**
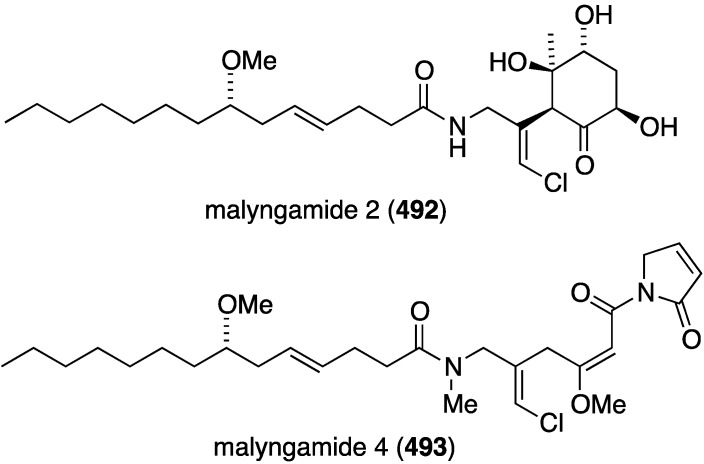
Structures of malyngamides 2 (**492**) from *Lyngbya sordida* and 4 (**493**) from *Moorea producens* [175,176].

The new malyngamide 2 (**492**) was characterized from a Papua New Guinea collection of *Lyngbya sordida* (Figure 94) [175]. Cytotoxicity towards H-460 (lung) is modest at IC_50_ 21 µM. The Red Sea *Moorea producens* (formerly *Lyngbya majuscula*) produces malyngamide 4 (**493**) (Figure 94), along with five known analogues [176]. This compound is weakly inhibitory to the human cancer cell lines MDA-MB-231, A549, and HT-29 (GI_50_ 44, 40, and 50 µM, respectively).

**Figure 95:**
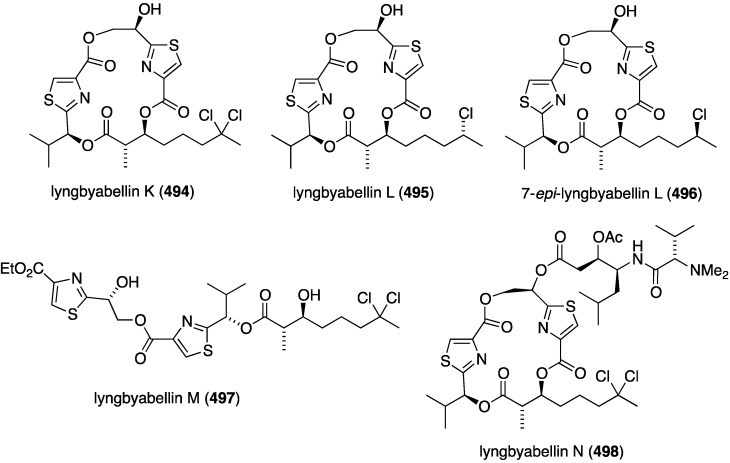
Structures of lyngbyabellins **494**–**498** from the cyanobacterium *Moorea bouillonii* [177].

The previously presented coibacins A–D (**297**–**300**) from the Panamanian *Oscillatoria* sp. (Figure 53) show cytotoxicity against the H460 (lung) human cancer cell line, with coibacin D having the highest activity (IC_50_ 11.4 µM) [114]. A collection of *Moorea bouillonii* from the Palmya Atoll in the Central Pacific Ocean has led to the discovery of five novel lyngbyabellins, **494**–**498** (Figure 95) [177]. Lyngbyabellin N (**498**) is very similar to the known lyngbyabellin H. Although **494**–**497** are inactive in the H-460 cytotoxicity screen, **498** shows a range of activity in this cell line, IC_50_ 0.0048–1.8 µM, which may result from solubility difficulties in the assay medium. However, in the HCT-116 colon cancer cell line, **498** gives the reproducible and very potent IC_50_ 40.9 ± 3.3 nM.

**Figure 96:**
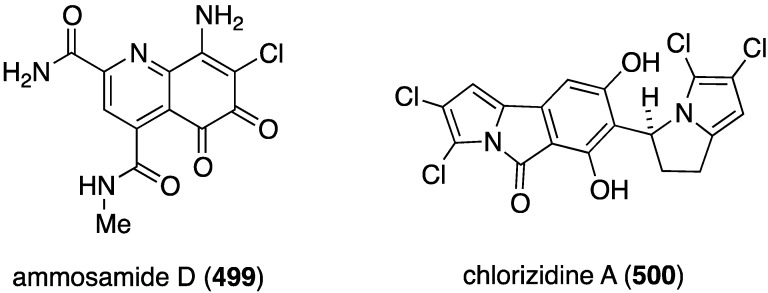
Structures of *Streptomyces* sp. ammosamide D (**499**) and chlorizidine A (**500**) [178,179].

Like terrestrial bacteria, marine bacteria can synthesize extremely complex natural products, most notably by marine-derived *Streptomyces* sp. A Bahamas marine sediment has provided *Streptomyces variabillis* (SNA-020) that produces ammosamide D (**499**) (Figure 96) [178]. This newest member of the ammosamide family has modest activity in the human cancer cell line MIA PaCa-2 (pancreas), IC_50_ 3.2 µM. Similarly, a marine sediment from the San Clemente, California, coast has yielded chlorizidine A (**500**) (Figure 96) [179]. This metabolite, with the unprecedented 5*H*-pyrrolo[2,1-*a*]isoindol-5-one ring system, is strongly cytotoxic to the human cell line HCT-116 (colon), IC_50_ 3.2–4.9 µM.

**Figure 97:**
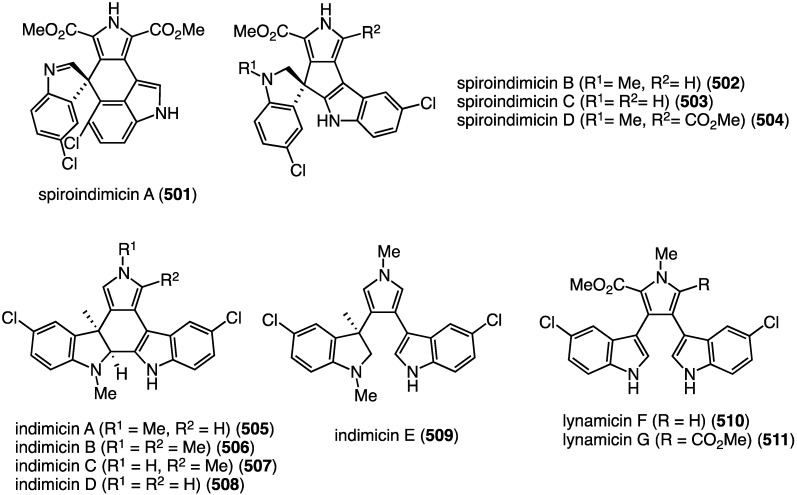
Structures of spiroindimicins A–D (**501**–**504**) and indimicins A–E (**505**–**509**) and lynamicins F (**510**) and G (**511**) from *Streptomyces* sp. SCS10 03032 [180,181].

The earlier discussed strepchloritides A (**46**) and B (**47**) (Figure 7), from *Streptomyces* sp. OUCMDZ-1703, are cytotoxic against the MCF-7 (breast) cell line; IC_50_ 9.9 and 20.2 µM, respectively [42]. The deep-sea derived *Streptomyces* sp. SCS10 03032 has provided the remarkable spiroindimicins A–D (**501**–**504**) (Figure 97) [180]. Spiroindimicin B (**502**) shows moderate activity against B16 (mouse melanoma), H460 (human lung), and CCRF-CEM (human leukemia): 5, 12, and 4 µg/mL, respectively. Spiroindimicin C (**503**) towards HepG2 (human hepatocellular liver) and H460 gives: 6 and 15 µg/mL, respectively. Spiroindimicin D (**504**) is slightly less active, and A (**501**) is inactive in all five cell lines, including MCF-7 (breast). For comparison, 5′-hydroxystaurosporine shows IC_50_ values of 8, 2, 8, and 5 µg/mL for HepG2, B16, H460, and CCRF-CEM, respectively. This same *Streptomyces* sp. contains indimicins A–E (**505**–**509**) and lynamicins F (**510**) and G (**511**) (Figure 97) [181]. Of this collection, only indimicin B (**506**) is cytotoxic to the MCF-7 cell line, IC_50_ 10.0 µM. No cytotoxicity is observed for the other indimicins when tested against SF268, MCF-7, H460, and HepG2.

**Figure 98:**
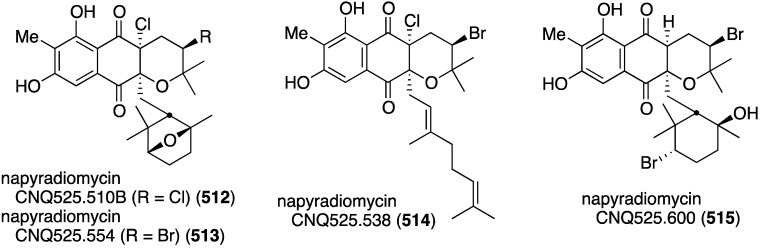
Structures of napyradiomycins **512**–**515** from actinomycete strain CNQ525 [182].

The aforementioned napyradiomycins **184**–**193** (Figure 30 and Figure 31) [75,76] display antitumor activity towards the human colon cell line HCT-116 with these cytotoxicity values (IC_50_ µg/mL): napyradiomycin A (**184**) (4.19), B (**185**) (>20), C (**186**) (>20), D (**187**) (16.1), E (**188**) (4.81), F (**189**) (9.42), B2 (3.18), B3 (**190**) (0.19), and B4 (1.41) [75]. The effect of chlorine on the cytotoxicity is noteworthy (*i.e.*, napyradiomycins A *vs.* B, and F *vs.* B2). A La Jolla, California, coastal sediment has afforded the actinomycete strain CNQ525, which produces the novel napyradiomycins **512**–**515** (Figure 98) [182]. Assays of these compounds against the HCT-116 human colon cell line are as follows for the most active napyradiomycins (IC_50_ µM): CNQ525.538 (**514**) (6), B1 (2), B3 (**190**) (3), A80915A (3), A80915B (<1), and A809150 (<1). The etoposide control has 1 µM.

**Figure 99:**
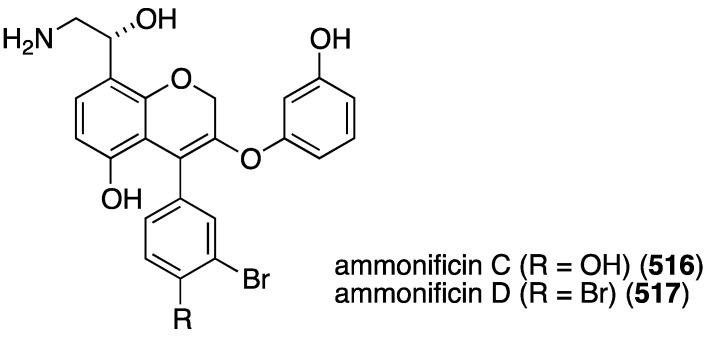
Structures of ammonificins C (**516**) and D (**517**) from *Thermovibrio ammonificans* [183].

The thermophilic bacterium *Thermovibrio ammonificans*, collected from the walls of a deep-sea hydrothemal vent on the East Pacific Rise, has provided two additional ammonificins, C (**516**) and D (**517**) (Figure 99) [183]. The *ortho* dibromophenyl ring is unique amongst natural organohalogens. Both ammonificins C and D induce apoptosis at 2 and 3 µM, respectively, in a standard apoptosis assay with W2 and D3 cells.

**Figure 100:**
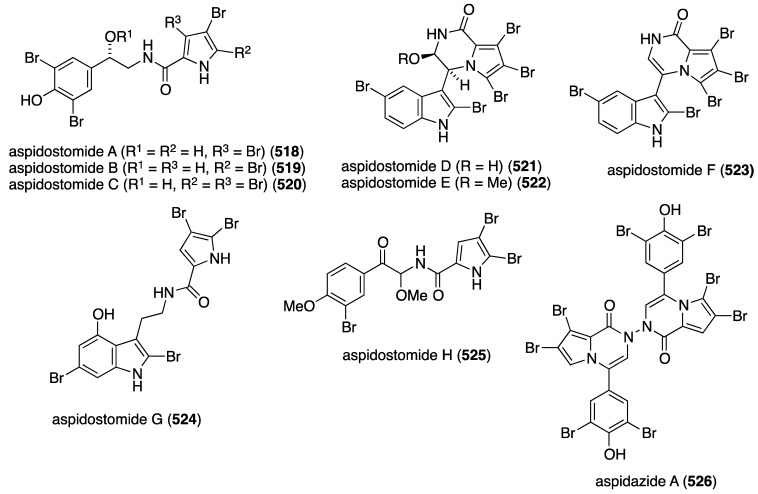
Structures of aspidostomides A–H (**518**–**525**) and aspidazide A (**526**) from the bryozoan *Aspidostoma giganteum* [184].

Despite their bland appearance, bryozoans (“moss animals”) are the repository of incredibly complex natural products, many of which are heavily brominated. The Patagonian bryozoan *Aspidostoma giganteum* contains a wealth of such organobromines, the aspidostomides A–H (**518**–**525**) and aspidazide A (**526**) (Figure 100) [184]. The only cytotoxic member (IC_50_ < 10 µM) of this collection is aspidostomide E (**522**), which displays IC_50_ 7.8 µM towards the human cell line 786-O (renal carcinoma).

**Figure 101:**
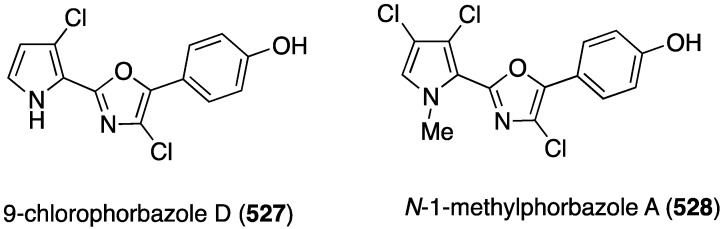
Structures of phorbazoles **527** and **528** from the nudibranch *Aldisa andersoni* [185].

The Indian Ocean nudibranch, *Aldisa andersoni*, has afforded two phorbazoles, 9-chlorophorbazole D (**527**) and *N*1-methylphorbazole A (**528**) (Figure 101), in addition to the known phorbazoles A, B, and D [185]. Both new phorbazoles show modest growth inhibition against the human cell lines A549, MCF-7, SKMEL-28 (melanoma), Hs683 (oligodendroglioma), and U373 (glioblastoma) in the range of IC_50_ 18–29 µM and 19–34 µM for **527** and **528**, respectively. These data are comparable or superior to the IC_50_ levels observed with carboplatin and temozolomide.

**Figure 102:**
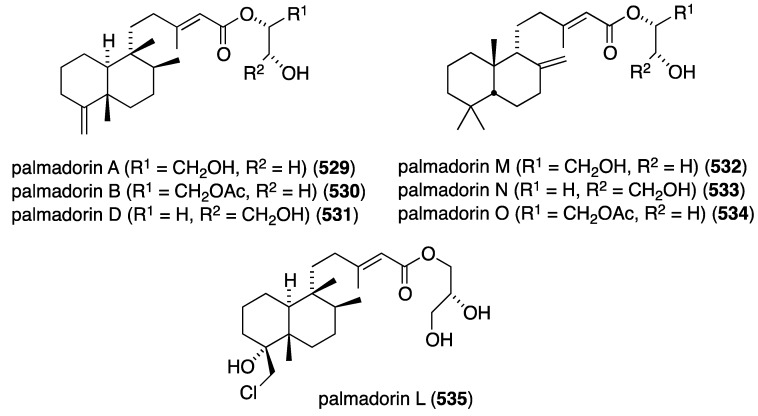
Structures of palmadorins (**529**–**535**) from the nudibranch *Austrodoris kerguelenensis* [186].

The Antarctic nudibranch *Austrodoris kerguelenensis*, collected near Palmer Station, produces sixteen new and some old diterpenoid glyceride esters, the palmadorins, several of which inhibit human erythroleukemia (HEL) cells. These are palmadorins A (**529**), B (**530**), D (**531**), M (**532**), N (**533**), and O (**534**) (Figure 102) [186]. One contains chlorine, the inactive palmadorin L (**535**) (Figure 102) [186]. The growth inhibition data for the active palmadorins are (IC_50_ µM): A (8.7), B (8.3), D (16.5), M (4.9), N (6.3), and O (13.4), respectively.

Larger marine animals like gastropod molluscs are known to produce biologically active metabolites, some of which contain halogen. The anticancer properties of the lamellarins, which were first isolated from a marine mollusc, have been reviewed [187].

**Figure 103:**
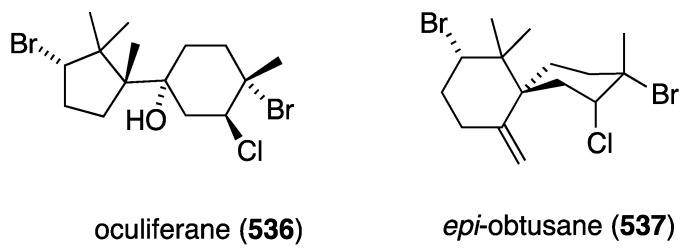
Structures of oculiferane (**536**) and *epi*-obtusane (**537**) from the sea hare *Aplysia oculifera* [190].

The Australian gastropod *Dicathais orbita* contains the well-known 6-bromoisatin which is active against the human cancer cell lines HT-29 and Caco2. It inhibits cell cycle progression of HT-29 cells by arresting some cells in the G2/M phase, and induces apoptosis [188,189]. The Egyptian sea hare *Aplysia oculifera* has provided two new halogenated sesquiterpenes, oculiferane (**536**) and *epi*-obtusane (**537**) (Figure 103) [190]. Both compounds are cytotoxic (IC_50_ < 10 µg/mL) to the human cell lines PC-3 (prostate), A549, MCF-7, HepG2, and HCT 116, with these IC_50_ values (**536**/**537**): 3.9/3.1, 3.1/0.96, 5.6/5.9, 3.3/2.4, and 5.9/4.1 µg/mL, respectively. **537** is comparable to 5-fluorouracil against A-549 (0.96 *vs.* 0.90 µg/mL).

## 7. Antioxidants and Antiinflammation

Because antioxidants can have anti-inflammatory activity, these two categories are combined.

**Figure 104:**
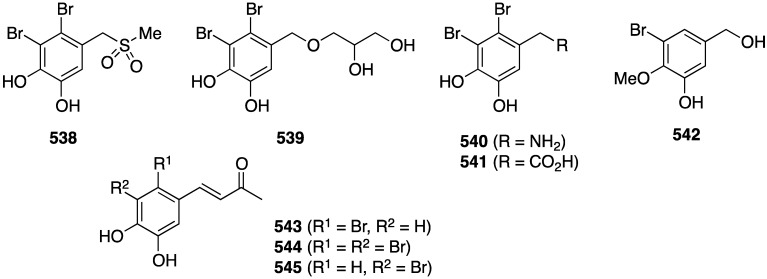
Structures of bromophenols **538**–**545** from the alga *Rhodomela confervoides* [191].

Like terrestrial phenolic compounds, marine phenols with antioxidant properties are well known, and several recent examples have appeared. The red alga *Rhodomela confervoides* from Liaoning Province, China, has afforded 19 bromophenols, six of which are new (**538**–**543**) (Figure 104) [191]. Two known examples, **544** and **545**, are included because they are active in the radical scavenging assays. All 19 bromophenols were subjected to both the DPPH (1,1-diphenyl-2-picrylhydrazyl) and the ABTS (2,2′-azinobis(3-ethylbenzothiazoline-6-sulfonic acid)diammonium salt) free radical scavenging assays. Most of the bromophenols display more potent antioxidant activity than either BHT (butylated hydroxytoluene) or ascorbic acid. For the DPPH assay the most active compound is **539** (IC_50_ 7.43 µM) followed by **544** > **543** > **545** > **538**. In the ABTS assay **543** is the most active, followed by **544** > **545** > **541**.

**Figure 105:**
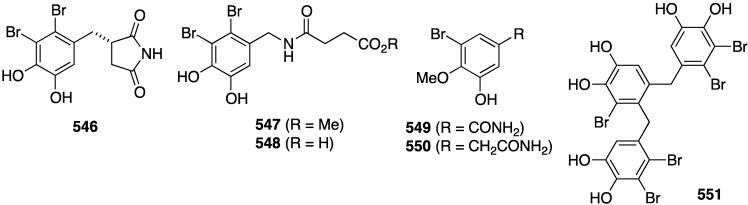
Structures of bromocatechols **546**–**551** from the alga *Rhodomela confervoides* [192].

Another study of *Rhodomela confervoides* has led to the discovery of five new nitrogen-containing bromophenols **546**–**550** (Figure 105) in addition to nine known analogues such as **551** [192]. In the DPPH assay bromophenol **546** shows the strongest activity (IC_50_ 5.22 µM) (BHT, IC_50_ 82.1 µM), followed by **548** > **547** > **551**. In the ABTS assay, **551** is the most active, more active than ascorbic acid. The antioxidant capacity of these bromophenols seems to be correlated with the number of hydroxyl groups (or phenolic rings).

**Figure 106:**
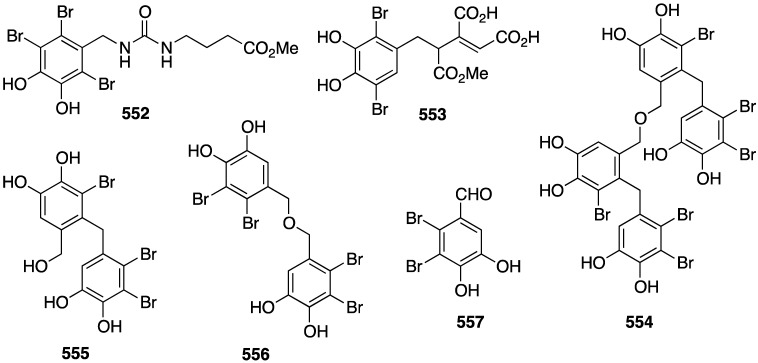
Structures of bromocatechols **552**–**556** from the red algae *Symphyocladia latiuscula* and *Vertebrata lanosa* [193,194].

A specimen of the red alga *Symphyocladia latiuscula* from the coast of Qingdao, Shandong Province, China, has furnished the new bromocatechols **552** and **553** (Figure 106) [193]. Both are modest radical scavengers in the DPPH assay with IC_50_ 14.5 and 20.5 µg/mL, respectively. Ascorbic acid shows IC_50_ 7.82 µg/mL. The red alga *Vertebrata lanosa*, collected from Ullsfjorden, Norway, afforded the new bromocatechol **554** and the known **555**–**557** (Figure 106) [194]. Their antioxidant capacity was screened using these assays: ORAC (oxygen radical absorbance capacity), CAA (cellular antioxidant activity), and CLPAA (cellular lipid peroxidation antioxidant activity). This study is the first to measure the cellular antioxidant activity of bromocatechols. The antioxidant activity is highest for **555** followed by **554**, and then **556** and **557**. At concentrations as low as 10 µg/mL, bromocatechol **555** inhibits 68% of oxidation in the CAA assay. By comparison, the known antioxidants quercetin and luteolin at this same concentration (10 µg/mL) inhibit the oxidation of the CAA substrate (2′,7′-dichlorofluorescin) to the extent of 92% and 58%, respectively.

**Figure 107:**
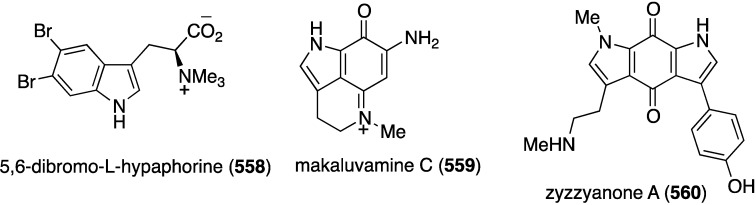
Structures of **558**–**560** from the sponges *Hyrtios* sp. and *Zyzza fuliginosa* [195,196].

Several marine sponges exhibit antioxidant behavior. The new 5,6-dibromo-l-hypaphorine (**558**) (Figure 107), along with four known bromoindoles, was isolated from the sponge *Hyrtios* sp. living in Fiji [195]. This new bromoindole displays significant antioxidant ability in the ORAC assay, only 4-fold less active than Trolox (a water-soluble analogue of Vitamin E). A study of the antioxidant activity of the known *Zyzzya fuliginosa* sponge metabolites, zyzzyanones and makaluvamines reveals that the presence of a phenolic ring is essential for maximum activity in both the ABTS and APPH assays, and that a *p*-hydroxystyryl unit as in the makaluvamines (e.g., **559**) is more important than a simple phenolic ring as in the zyzzyanones (e.g., **560**) (Figure 107) [196].

**Figure 108:**
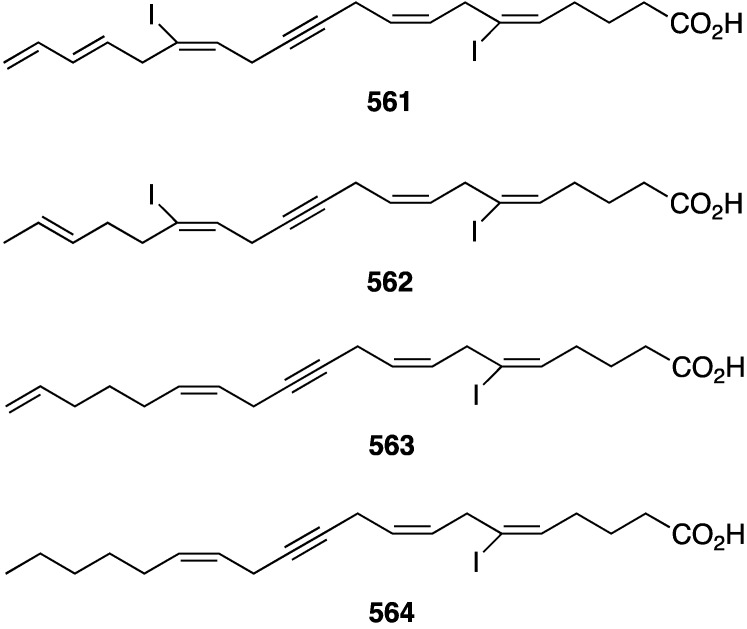
Structures of iodinated acetylenic acids **561**–**564** from the sponges *Suberites mammilaris* and *S. japonicus* [197].

The novel iodinated acetylenic acid sponge metabolites **561**–**564**, isolated from the South Korean *Suberites mammilaris* (**561** and **562**) and *Suberites japonicus* (**563** and **564**), were examined for their antiinflammatory activity (Figure 108) [197]. The methyl esters **561** and **562** strongly inhibit nitric oxide (NO) production from RAW 264.7 murine macrophase cells, with IC_50_ 3.9 and 7.0 µM, respectively. However, in BV2 microglia cells, the methyl esters of **563** and **564** are the most active in NO inhibition: IC_50_ 3.1 and 1.8 M, respectively. All four methyl esters attenuate the production of PGE_2_ (prostaglandin E2) from RAW 264.7 and BV2 cells as induced by LPS (lipopolysaccharide).

**Figure 109:**
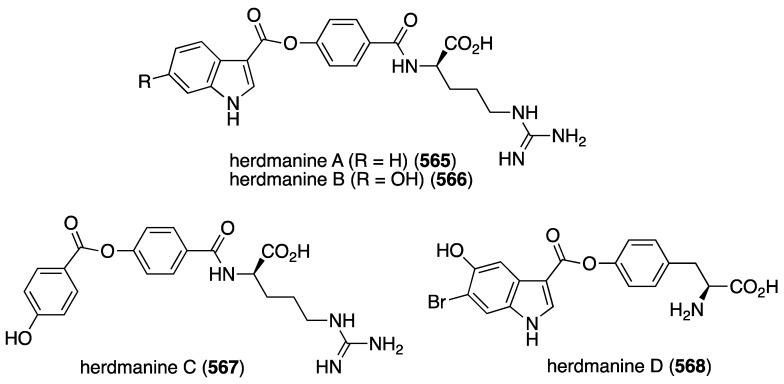
Structures of herdmanines A–D (**565**–**568**) from the ascidian *Herdmania momus* [198].

The previously cited 4,5,6-tribromo-2,3-bis(methylthio)indole (**121**) (Figure 21) dramatically reduces the expression of both the pro-inflammatory enzyme *i*-NOS (inducible nitric oxide synthase) and COX-2 (cyclooxygenase) in LPS-activated RAW 264.7 cells. This indole has superior antiinflammatory activity relative to the other bromoindoles in this study [62]. Likewise, pitinoic acid B (**199**) (Figure 33) in LPS-stimulated differentiated THP-1 (human acute monocytic leukemia) cells decreases the level of the pro-inflammatory cytokines TNF-α (tumor necrosis factor alpha) and IL-6 (interleukin 6), which probably accounts for the antiinflammatory effects of **200** [79]. Coibacin B (**298**) (Figure 53) also inhibits the gene transcription of the cytokines TNF-α, IL-6, IL-1b, and *i*-NOS. In the latter assay for NO production **298** has IC_50_ 5 µM [114]. The didemnins from the tunicate *Trididemnum solidum* (Figure 80) have pronounced antiinflammatory activity, particularly didemnin B (**405**), which inhibits *i*-NOS and NF-κB (nuclear factor-kappa B) expression, with IC_50_ 0.002 and 0.03 µM, respectively. Chlorinated didemnin **402** shows IC_50_ 0.4 and 0.26 µM, respectively [159]. Malyngamide 2 (**492**) (Figure 94) has a value of IC_50_ 8.0 µM in LPS-induced RAW 264.7 macrophages for the inhibition of NO production [175]. The herdmanines A–D (**565**–**568**) (Figure 109) from the Korean ascidian *Herdmania momus* inhibit the mRNA expression of *i*-NOS, and thereby suppress NO production with IC_50_ values of 90 and 9 µM, for **567** ad **568**, respectively. These two herdmanines also inhibit PGE_2_ production via the reduced mRNA expression of COX-2, and herdmanine D (**568**) inhibits the mRNA expression of IL-6 [198].

**Figure 110:**
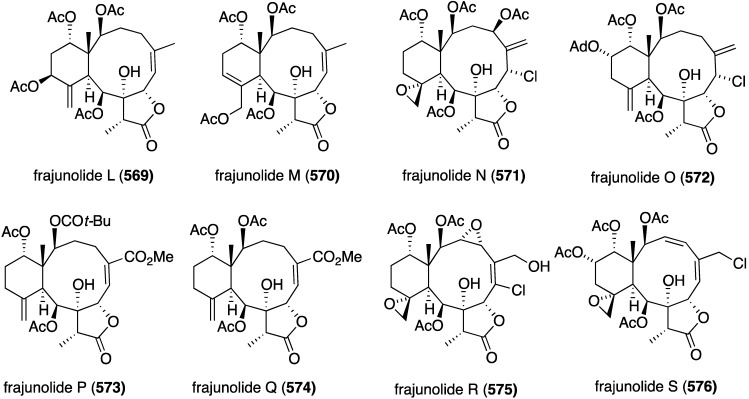
Structures of frajunolides L–S (**569**–**576**) from the gorgonian *Junceella fragilis* [199,200].

**Figure 111:**
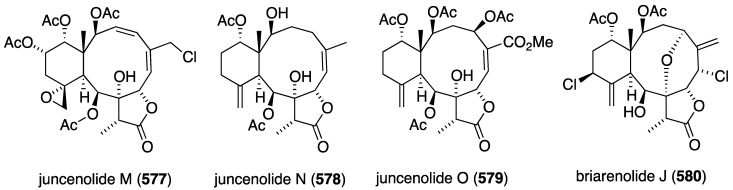
Structures of juncenolides M–O (**577**–**579**) from the gorgonian *Junceella juncea* [201].

The Taiwanese gorgonian *Junceella fragilis* has afforded eight new 8-hydroxybriarane diterpenoids, frajunolides L–O (**569**–**572**) [199] and P–S (**573**–**576**) [200] (Figure 110). The antiinflammatory activities of these frajunolides were examined by measuring superoxide generation and elastase release by human neutrophils in response to fMLP/CB (formylmethionyl-leucyl-phenylalanine/dihydrocytochalasin B. These data are summarized in Table 17. A similar set of briarane diterpenoids was characterized in the gorgonian *Junceella juncea*, juncenolides M–O (**577**–**579**) (Figure 111) [201]. The antiinflammatory activity of these juncenolides is shown in Table 17. Of the frajunolides L–S, P and Q are the most active on both superoxide anion generation and elastase release. Of the juncenolides M–O, O is the most active and N shows inhibition against elastase release. The gorgonian *Briareum* sp. collected in Taiwan yielded the novel dichlorinated briarenolide J (**580**) (Figure 111), which also displays antiinflammatory activity (Table 17) [202]. It would appear that frajunolide S (**576**) is identical with juncenolide M (**577**).

**Table 17:** Effect of frajunolides L–S (**569**–**576**), juncenolides M–O (**577**–**579**), and briarenolide J (**580**) on superoxide anion generation and elastase release in response to fMLP/CB.

	% Inhibition ^a^	
Compound	Superoxide Anion	Elastase Release
frajunolide L (**569**)	18.7	16.2
frajunolide M (**570**)	2.0	13.3
frajunolide N (**571**)	0.6	22.3
frajunolide O (**572**)	8.3	17.2
frajunolide P (**573**)	32.5	35.6
frajunolide Q (**574**)	28.7	34.1
frajunolide R (**575**)	9.7	16.0
frajunolide S (**576**)	5.8	−4.5
juncenolide M (**577**)	7.6	15.9
juncenolide N (**578**)	6.7	29.0
juncenolide O (**579**)	27.6	35.9
briarenolide J (**580**)	14.98	9.96
genistein	65.0	51.6

^a^ % inhibition at 10 µg/mL concentration.

**Figure 112:**
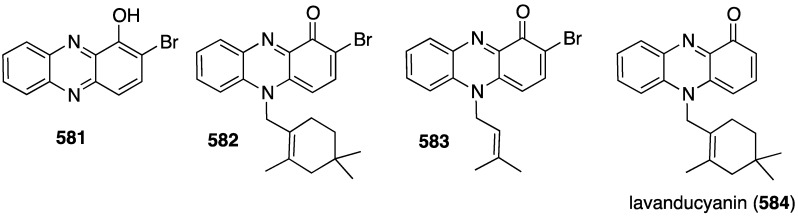
Structures of phenazines (**581**–**584**) from *Streptomyces* sp. CNS284 [203,204].

Marine bacteria have yielded some antiinflammatory compounds, such as *Streptomyces* sp. CNS284 which produces 2-bromo-1-hydroxyphenazine (**581**) (Figure 112), which has some activity in the NF-κB-luciferase assay (IC_50_ 73 µM) [203]. Several synthetic analogues are more active than **581** and show potent inhibition of *i*-NOS expression and display chemoprevention, QR1 (quinone reductase 1) induction and QR2 (quinone reductase 2) inhibition. The two novel phenazines, **582** and **583**, were characterized from the same *Streptomyces* sp. CNS284 along with the known lavanducyanin (**584**) (Figure 112) [204]. All three phenazines inhibit TNF-α-induced NF-κB activity (IC_50_ 4.1, 24.2, and 16.3 μM, respectively), and LPS-induced NO production (IC_50_ > 48.6, 15.1, and 8.0 µM, respectively). The blocking of PGE_2_ production was even more efficient (IC_50_ 7.5, 0.89, and 0.63 µM, respectively). This study also shows that lavanducyanin inhibits the activity of COX-1 and COX-2, in addition to the production of NO and PGE_2_.

## 8. Enzymatic and Molecular Activity

Overshadowed by the biological effects presented in the previous sections are molecular interactions between the marine natural products and the target molecules (enzymes, peptides, and other small biological molecules) that are the root cause of these effects. A review of the targeting of marine natural products to cytoskeletal proteins has appeared [205].

**Figure 113:**
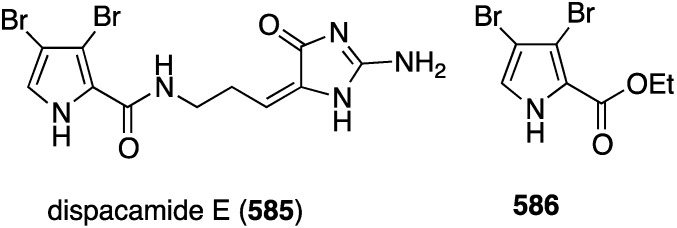
Structures of dispacamide E (**585**) and pyrrole **586** from the sponges *Stylissa massa* and *Stylissa flabelliformis* [207].

**Figure 114:**
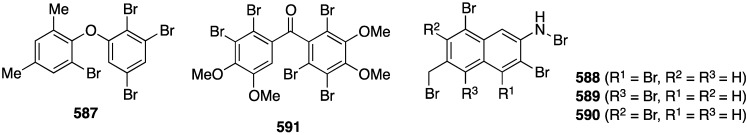
Structures of polybromides **587**–**591** from the red alga *Laurencia similis* [208].

Several marine brominated natural products are protein kinase inhibitors. Purpuroines A (**59**) and D (**62**) (Figure 11) are selective inhibitors of the kinase LCK (lymphocyte-specific protein tyrosine kinase) with IC_50_ 2.35 and 0.94 µg/mL, respectively. Purpuroine D is inhibitory towards PLK1 (serine/threonine-protein kinase) with IC_50_ 0.94 µg/mL. For comparison, staurosporine shows IC_50_ 3.73 and 0.92 µg/mL for LCK and PLK1, respectively. All of the purpuroines are weak inhibitors to CDK2 (cyclin-dependent kinase 2) (IC_50_ > 50 µg/mL) [46]. The study of the massadines (Figure 12) re-established that the known debromohymenialdisine and hymenialdisine are nanomolar kinase inhibitors of CDK5/P25 (cyclin-dependent kinase 5), CD1δ (casein kinase 1), and GSK3β (glycogen synthase kinase 3β): IC_50_ 0.4, 0.1, and 0.2 µM, respectively, for debromohymenialdisine, and IC_50_ 0.16, 0.03, and 0.07 µM, respectively, for hymenialdisine [47]. The novel sesquibastadin 1 (**367**) and bastadin 3 (Figure 71) are strong inhibitors of at least 22 protein kinases (IC_50_ 0.1–6.5 µM). For example sesquibastadin 1 causes potent inhibition of the receptor tyrosine kinases EGF-R and VEGF-R2 (both IC_50_ 0.6 µM), and of T1E2 (IC_50_ 0.6 µM). Bastadin 3 is a potent inhibitor of Aurora A and B (IC_50_ 0.1 and 0.5 µM, respectively). This bastadin inhibits all of the examined kinases at submicromolar activity. The other bastadins 6, 7, 11, and 16 are either inactive or much less active, exactly the opposite to their cell proliferation inhibitory activity (*vide supra*) [139]. A study of the known ageladine A, and synthetic analogues, against a battery of kinases shows that ageladine A has modest activity towards the tyrosine kinase DYRK1A and Pim 1 [206]. The Indonesian sponges *Stylissa massa* and *Stylissa flabelliformis* yielded 25 bromopyrroles, including the new dispacamide E (**585**) and **586** (Figure 113) [207]. All isolated compounds were assayed against these protein kinases: DYRK1A, CDK5, GSK-3, CLK-1, CK-1, CDK1, CDK2/A, CDK9/cyclin T, and *Plasmodium falciparum* glycogen synthase kinase-3 (*Pf*GSK-3). Dispacamide E is particularly active against GSK-3, DYRK1A and CK-1 (IC_50_ 2.1, 6.2, and 4.9 µM, respectively). The known hymenine and some hymenialdisine derivatives are very active against *Pf*GSK-3 with IC_50_ in the nanomolar range [207]. The red alga *Laurencia similis* from the Hainan coast, China, has afforded five new polybrominated compounds, **587**–**591** (Figure 114) [208]. The brominated *N*-bromo-2-naphthylamines **588**–**590** are remarkably unique structures, unlike the brominated diphenyl ether **587** and benzophenone **591**, for which many examples are known. Metabolites **587** and **591** are inhibitory towards PTP1B (protein tyrosine phosphatase B) with IC_50_ 2.97 and 2.66 µM, respectively. The Yesinia outer protein (YopE), which is also a protein tyrosine phosphatase, is inhibited by pseudoceramines B (**52**) and D (**54**) (Figure 9), with IC_50_ 19 and 6 µM, respectively [44]. This enzyme is essential for bacterial virulence of the Gram-negative *Yersinia* spp.

**Figure 115:**
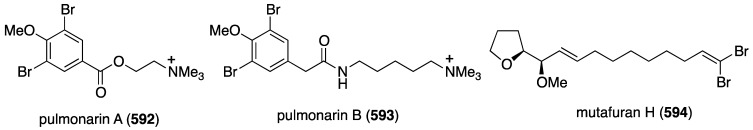
Structures of pulmonarins A (**592**) and B (**593**) from the ascidian *Synoicum pulmonaria* [209] and mutafuran H (**594**) from the sponge *Xestospongia testudinaria* [210].

The known helicusin A which was isolated from the fungus *Bartalina robillardoides* strain LF550 along with three new chloroazaphilones (Figure 27), shows inhibition of acetylcholinesterase (IC_50_ 2.1 µM) (the positive control hyperzine has IC_50_ < 0.1 µM). In this study the known deacetylsclerotiorin inhibits phosphodiesterase 4 (IC_50_ 2.79 µM), as does isochromophilone XI (**164**), albeit weaker (IC_50_ 8.30 µM). (The positive control rolipam has 0.75 µM) [71]. The new pulmonarins A (**592**) and B (**593**) (Figure 115), isolated from the clonial ascidian *Synoicum pulmonaria* living on the coast of Tromsø, Norway, are reversible, noncompetitive inhibitors of acetylcholinesterase; K_i_ = 90 µM and 20 µM, respectively. Relative to **593**, the Calabar bean alkaloid physostigmine has K_i_ = 30 nM [209]. The South China Sea sponge *Xestospongia testudinaria* has yielded the novel mutafuran (**594**) (Figure 115) along with three known bromine-containing polyacetylenes [210]. Mutafuran shows significant acetylcholinesterase activity (IC_50_ 0.64 µM). The positive control tacrine, which is used to treat early stage Alzheimer’s disease, has IC_50_ 0.41 µM.

**Figure 116:**
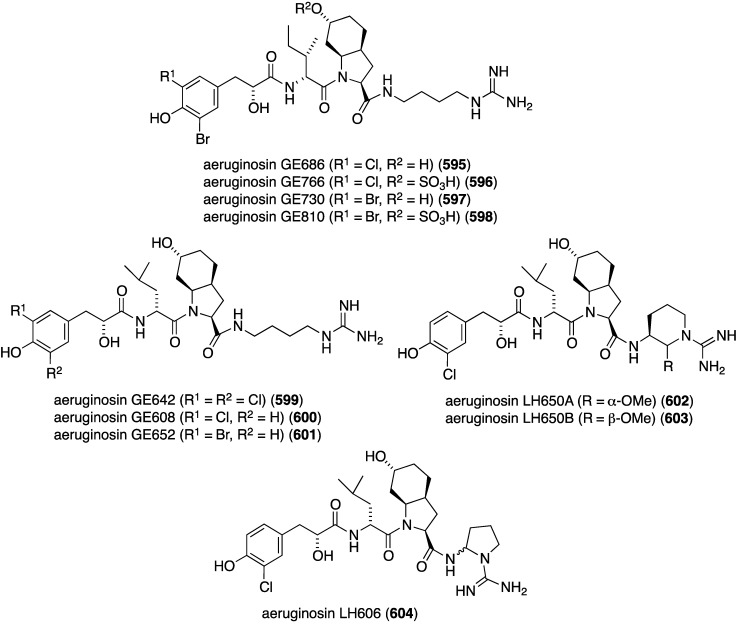
Structures of aeruginosins **595**–**604** from the cyanobacteria *Microcystis aeruginosa* and *Microcystis* spp. [211,212,213].

Several marine organohalogens discovered in the timeframe of this survey are protease inhibitors. The *Ianthella* sp. sponge metabolites dictyodendrin F, H, I, and J (Figure 14) are potent inhibitors of BACE 1 (β-secretase 1) with IC_50_ values of 1.0–2.0 µM). Only dictyodendrin G (**75**) is inactive [49]. The known cyanobacterium fischerindole hapalosin inhibits the 20s proteasome (IC_50_ 12 µM), whereas the other fischerindoles isolated in this study (Figure 92) are inactive [173]. Extensive studies of the cyanobacteria *Microcystis aeruginosa* and *Microcystis* spp. in Israel and India have revealed several novel aeruginosins. These are aeruginosin GE686 (**595**), GE766 (**596**), GE730 (**597**), GE810 (**598**), GE642 (**599**) [211], IN608 (**600**), IN652 (**601**) [212], LH650A (**602**), LH650B (**603**), LH606 (**604**), and the nonchlorinated microviridin LH1667 [213] (Figure 116). Several known analogues were also isolated from these blooms. The aeruginosins are inhibitors of the serine proteolytic enzymes trypsin and thrombin. The trypsin inhibitory activities (IC_50_ µM) are best realized for GE686 (**595**), 3.2; GE730 (**597**), 2.3; IN608 (**600**), 4.3; IN652 (**601**), 4.1; and LH606 (**604**), 18.5. The thrombin inhibitory activities (IC_50_ µM) are best seen for GE686 (**595**), 12.8; GE730 (**597**), 12.9; LH650A (**602**), 1.8; LH650B (**603**), 1.8; and LH606 (**604**) 2.5. Microviridin inhibits chymotrypsin, IC_50_ 2.8 µM [211,212,213].

**Figure 117:**
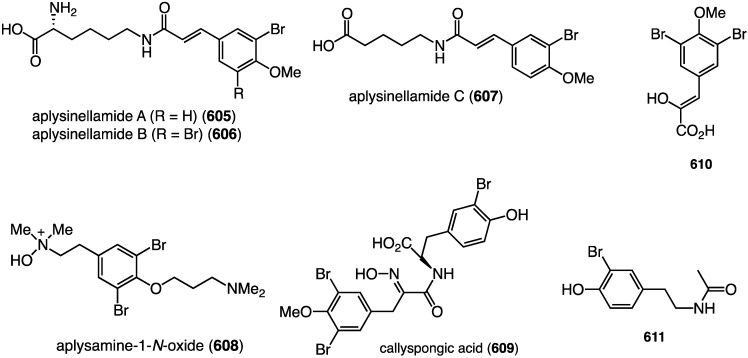
Structures of bromotyrosines **605**–**611** from the sponges *Aplysinella* sp. and *Callyspongia* sp. [214,215].

Some marine sponge metabolites increase the production of ApoE (apoliproprotein E), an important enzyme that mediates cholesterol metabolism, which has implication in the treatment of Alzheimer’s disease. The Great Barrier Reef, Australia, sponge *Aplysinella* sp. has afforded three new aplysinellamides A–D (**605**–**607**) and aplysamine-1-*N*-oxide (**608**) (Figure 117) along with six known analogues. Amongst the latter, aplysamine-1 displays ApoE-modulating activity by increasing by 2-fold the secretion of ApoE from human astrocytoma cells at a concentration of 30 µM [214]. Likewise, the Australian sponge *Callyspongia* sp. has yielded the new bromotyrosines **609**–**611**, along with ten known compounds (Figure 117). Of these, **610** shows weak ability to increase ApoE from human astrocytoma cells (CCF-STTG1) at a concentration of 40 µM [215].

**Figure 118:**
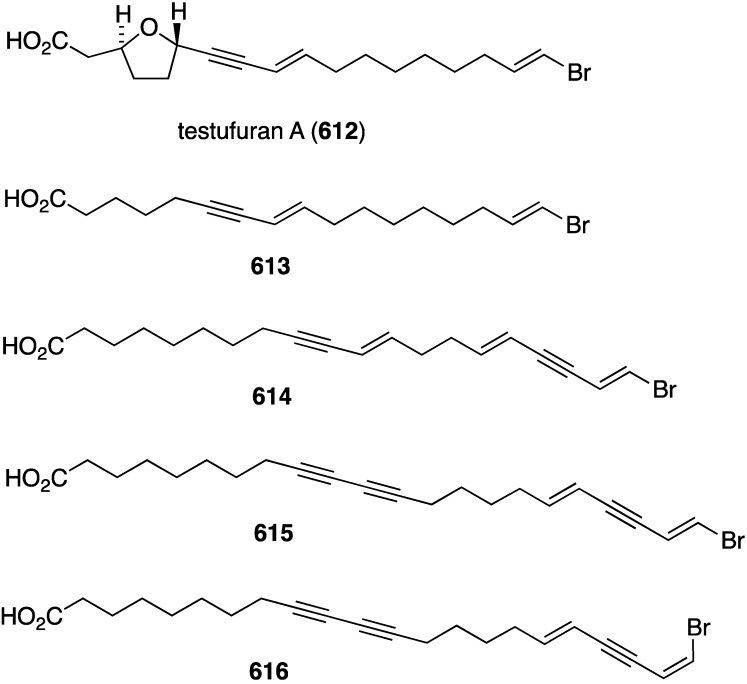
Structures of bromo carboxylic acids **612**–**616** from the sponge *Xestospongia testudinaria* [216].

The sponge *Xestospongia testudinaria* has yielded five new brominated fatty acids, **612**–**616** (Figure 118), which include testufuran A (**612**), similar to mutafuran H (**594**) (Figure 115) isolated from the same sponge. An additional 11 known brominated acetylenic acids were also characterized. Most of these 16 bromo carboxylic acids stimulated the secretion of the protein hormone adiponectin, which regulates glucose levels and fatty acid breakdown, from differentiated ST-13 preadipocytes. These compounds do not exhibit agonistic activity against PPAR-γ (the peroxisome proliferator-activated receptor) [216].

**Figure 119:**
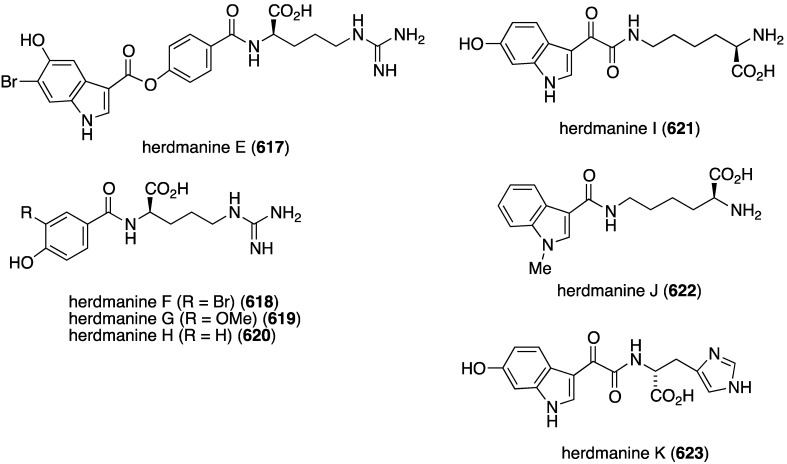
Structures of herdmanines E–K (**617**–**623**) from the ascidian *Herdmania momus* [217].

The ascidian *Herdmania momus* has yielded seven new herdmanines E–K (**617**–**623**) (Figure 119), some of which demonstrate significant PPAR-γ activation in Ac2F rat liver cells. The active examples are I (**621**) and K (**623**). For example, the latter herdmanine K exhibits strong PPAR-γ activation at 1 and 10 µg/mL concentrations, with greater potency than the antidiabetic drug rosiglitazone. The known (–)-leptoclinidamine B was also isolated from the ascidian and is only slightly less active than **623** [217].

**Figure 120:**
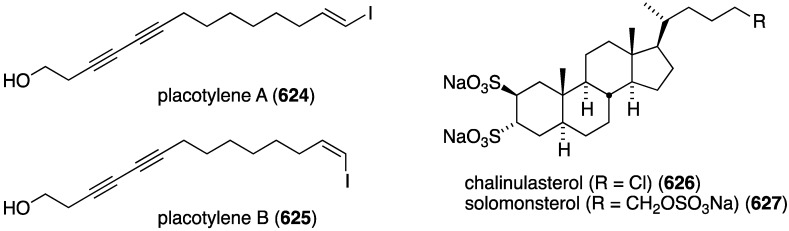
Structures of placotylenes A (**624**) and B (**625**) from the sponge *Placospongia* sp. [218] and chalinulasterol (**626**) from the sponge *Chalinula molitba* [219].

The two rare iodinated polyacetylenes, placotylenes A (**624**) and B (**625**), were characterized in the Korean sponge *Placospongia* sp. (Figure 120). Placotylene A inhibits osteoclast differentiation of bone marrow-derived macrophages, perhaps by decreasing the expression of RANKL (receptor activator of nuclear factor-κB ligand). This marine polyacetylene could represent a lead compound for osteoporosis treatment [218]. The Caribbean sponge *Chalinula molitba* has afforded the novel chlorinated sterol disulfate, chalinulasterol (**626**) (Figure 120). Despite the resemblance of chalinulasterol to the known PXR (pregnane X receptor) agonist solomonsterol A (**627**), no activity is observed for the former sterol. This important receptor regulates expression of drug metabolizing and detoxifying enzymes [219].

Two sets of metabolites from the ascidian *Synoicum* sp. exhibit inhibition of the peptidase-type proteins sortase A and isocitrate lyase, two enzymes that have important functions in the virulence and survival of pathogenic bacteria. Thus, of the eudistomins cited earlier (Figure 23), Y_4_ (**134**) and Y_5_ (**135**) show modest activity toward sortase A (SrtA) (IC_50_ 163.2 and 146.4 µM, respectively), whereas Y_2_ (**132**) shows IC_50_ 50.2 µM against isocitrate lyase (ICL) [65]. Of the brominated aromatic furanones examined (Figure 24), cardiolide E (**138**) inhibits SrtA (IC_50_ 78.8 µM), and cardiolides E (**138**) and I (**142**) show IC_50_ 8.9 and 10.8 µM, respectively, for SrtA [67]. These *Synoicum* metabolites also inhibit the enzyme Na^+^/K^+^-ATPase (sodium-potassium adenosine triphosphatase) as follows: Y_4_ (**134**), Y_6_ (**136**), Y_7_ (**137**), cardiolide E (**138**), and cardiolide I (**142**) give these values: 7.5, 10.1, 11.3, 2.5, and 5.0 µM, respectively [65,67]. This enzyme is a sodium-potassium pump with several functions.

**Figure 121:**
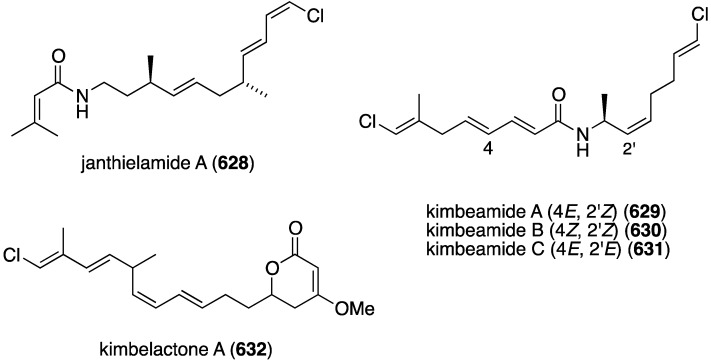
Structures of janthielamide A (**628**), kimbeamides A–C (**629**–**631**), and kimbelactone A (**632**) from cyanobacteria [220].

A combined Curacao and Papua New Guinea collection of cyanobacteria has yielded five new vinylchloride metabolites, janthielamide A (**628**), kimbeamides A–C (**629**–**631**), and kimbelactone A (**632**) (Figure 121). Janthielamide A came from the collection at Jan Thiel Bay in Curacao, and the latter four metabolites came from the collection at Kimbe Bay, New Britain, Papua New Guinea. Janthielamide A (**628**) exhibits Na^+^ channel blocking in murine Neuro-2a cells (IC_50_ 11.5 µM), and also antagonizes induced Na^+^ influx in neurons (IC_50_ 5.2 µM). Kimbeamide A (**629**) displays similar Na^+^ blocking activity at a concentration of 20 µg/mL, but it, along with the **630**–**632**, undergoes oxidative decomposition [220].

**Figure 122:**
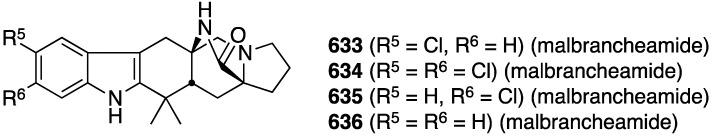
Structures of malbrancheamides **633**–**636** from the fungus *Malbranchea aurantiaca* [221].

The new isomalbrancheamide B (**633**), along with three known analogues, was isolated from the fungus *Malbranchea aurantiaca* (Figure 122). Isomalbrancheamide B (**633**) and the known malbrancheamide (**634**) and malbrancheamide B (**635**) are classical CaM (calmodulin) inhibitors, whereas the nonchlorinated premalbrancheamide (**636**) is not. Malbrancheamide (**634**) is the most active, and it binds to the same hydrophobic pocket as the antipsychotics chlorpromazine and trifluoperazine, two classical CaM inhibitors [221].

**Figure 123:**
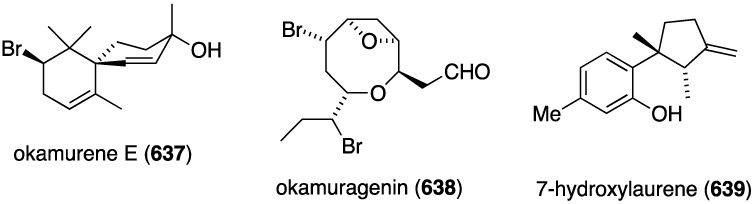
Structures of okamurene E (**637**) and okamuragenin (**638**) from the red alga *Laurencia okamurai*, and 7-hydroxylaurene (**639**) [222].

The previously cited 5,6-dibromo-l-hypaphorine (**558**) (Figure 107) from the sponge *Hyrtios* sp. is a weak inhibitor of bee venom phospholipase A_2_ (PLA_2_). Relative to the positive control, manoalide (IC_50_ 0.5 µM), **558** has IC_50_ 0.20 mM [195]. The red alga *Laurencia okamurai* has yielded the new chamigrane, okamurene E (**637**), and the new C_12_-acetogenin, okamuragenin (**638**) (Figure 123), along with the known okamurenes A–D and nine known sesquiterpenes and four known C_15_-acetogenins. All of these compounds were evaluated for toxicity against brine shrimp (*Artemia salina*). Of all compounds, only 7-hydroxylaurene (**639**) expressed lethal toxicity with LD_50_ 1.8 µM [222].

**Figure 124:**
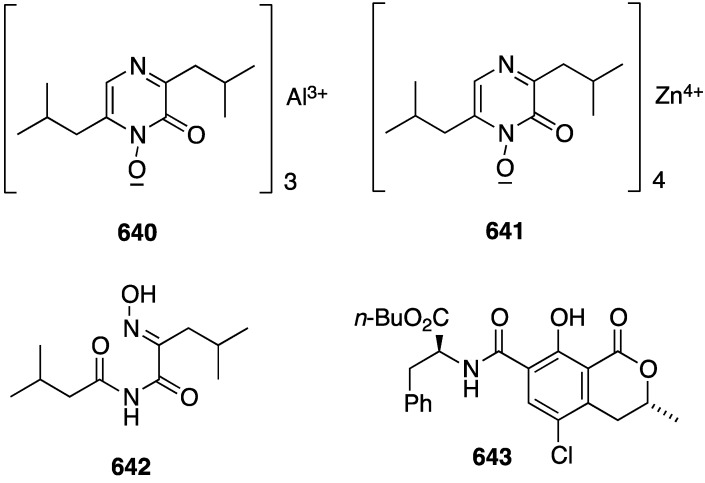
Structures of mycotoxins **640**–**643** from the fungus *Aspergillus* sp. SCSGAF0093 [223].

The marine-derived fungus *Aspergillus* sp. SCSGAF0093 produces nine mycotoxins, four of which are new, aluminiumneoaspergillin (**640**), zirconiumneoaspergillin (**641**), aspergilliamide (**642**), and ochratoxin A *n*-butyl ester (**643**) (Figure 124). This is the first report of marine-based ochratoxins (ochratoxin and the methyl ester were also isolated), and the first discovery of a zirconium complex (**641**) in nature [223]. All nine compounds exhibit some toxicity to brine shrimp. The most toxic compounds in this assay are **643**, ochratoxin A, and ochratoxin A methyl ester, with IC_50_ 4.14, 13.74, and 2.59 µM, respectively.

The innocent-looking, but ominous cone snails (genus *Conus*) comprise about 700 species and are widely distributed in the world’s oceans [224]. It is estimated that these cone snails contain more than 50,000 distinct toxins, since the venom in each *Conus* species consists of 40–200 individual peptides with a unique biological action [225,226,227]. Many of these *Conus* sp. peptides contain 6-bromotryptophan [3], the function of which has been suggested to block proteolytic degradation since the large bromine makes the peptide a poor fit for docking in the active site of chymotrypsin [228]. Recent studies have established the binding site of α-conotoxin Vc1.1 from *Conus victoria* on the nicotinic α9α10 acetylcholine receptor, making this toxin a potential novel treatment for neuropathic pain [229]. A similar α-4/6-conotoxin TxID has been identified in *Conus textile*. It also blocks nicotinic acetylcholine receptors [230]. The conopeptide MVIIA (Ziconotide; Prialt) was approved by the U.S. FDA in 2004 for the treatment of severe pain.

## 9. Conclusions

Marine organisms possess an astonishing array of biological activities! The chemical compounds they produce proffer future medicinal developments in a multitude of human diseases. Of these compounds, organohalogen natural products frequently display the highest level of biological activity. The unceasing developments in aquatic exploration, organism collection, compound isolation and identification, and biological assays guarantee that new marine natural products are awaiting discovery, biological evaluation, and possible benefit to mankind.
